# Optically Distinguishable Electronic Spin-isomers of a Stable Organic Diradical

**DOI:** 10.1021/acscentsci.4c00284

**Published:** 2024-04-08

**Authors:** Daiki Shimizu, Hikaru Sotome, Hiroshi Miyasaka, Kenji Matsuda

**Affiliations:** † Department of Synthetic Chemistry and Biological Chemistry, Graduate School of Engineering, Kyoto University, Nishikyo-ku, Kyoto 615-8510, Japan; ‡ Division of Frontier Materials Science and Center for Promotion of Advanced Interdisciplinary Research, Graduate School of Engineering Science, Osaka University, Toyonaka, Osaka 560-8531, Japan; § Fukui Institute for Fundamental Chemistry, Kyoto University, Sakyo-ku, Kyoto 606-8103, Japan

## Abstract

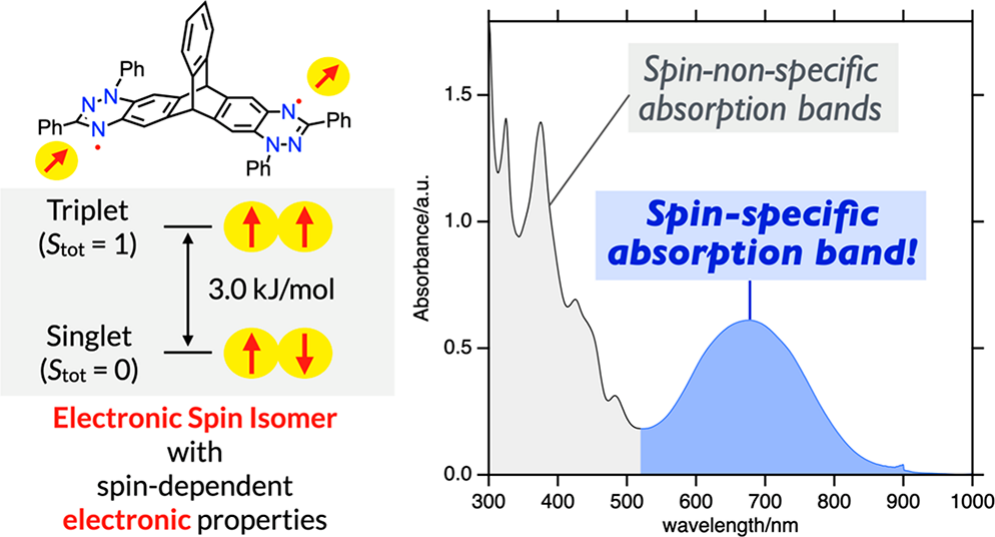

Herein, we introduce a model of electronic spin isomers, the electronic counterpart of nuclear spin isomers, by using a stable organic diradical. The diradical, composed of two benzotriazinyl radicals connected by a rigid triptycene skeleton, exhibits a small singlet–triplet energy gap of −3.0 kJ/mol, indicating ca. 1:1 coexistence of the two spin states at room temperature. The diradical shows characteristic near-IR absorption bands, which are absent in the corresponding monoradical subunit. Variable temperature measurements revealed that the absorbance of the NIR band depends on the abundance of the singlet state, allowing us to identify the NIR band as the singlet-specific absorption band. It enables photoexcitation of one of the two spin states coexisting in thermal equilibrium. Transient absorption spectroscopy disclosed that the two spin states independently follow qualitatively different excited-state dynamics. These results demonstrate a novel approach to the design and study of electronic spin isomers based on organic diradicals.

## Abstract 2

We demonstrate that an organic diradical can be regarded as its electronic counterpart of nuclear spin isomers (e.g., *ortho*/*para*-hydrogens) with spin bistability and spin-dependent properties.

## Introduction

**Figure 1:**
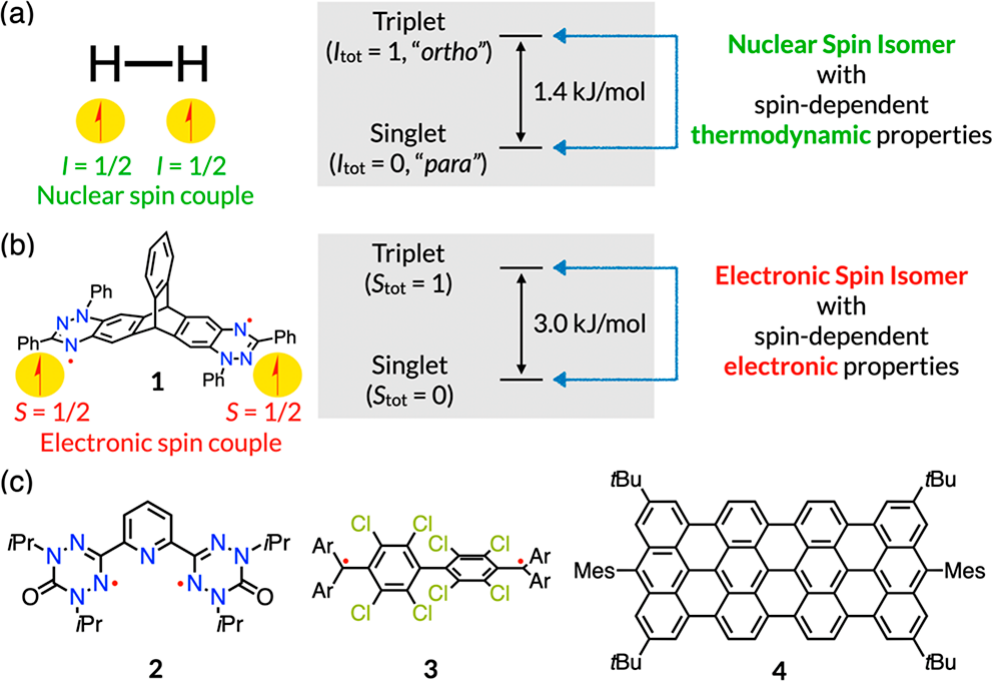
(a) Nuclear spin isomers of molecular hydrogen and (b) electronic spin isomers of **1** proposed in this work. (c) Molecules discussed herein. Ar = C_6_Cl_5_.

Spin isomers are a set of molecules that share the same chemical structure but are different in the spin state. The two forms of molecular hydrogen (*ortho*-/*para*-hydrogens) were first proposed by Heisenberg and Hund in 1927 to explain the abnormal rotational spectrum and specific heat of hydrogen (Figure 1a).^1,2^ The hydrogen (^1^H) atom has a nuclear spin of 1/2; hence the total nuclear spin quantum number of H_2_ is 0 (singlet, *para*) or 1 (triplet, *ortho*). The *ortho*- and *para*-hydrogens are energetically close to each other (|Δ*E*| = 1.4 kJ/mol for the lowest rotation modes), but their interconversion is a slow, spin-forbidden process.^3^ Therefore, the two spin states can be individually treated as (meta-)stable species. Furthermore, the *ortho*- and *para*-hydrogens are fundamentally contrasting in permutation symmetry, leading to specific rotation modes and contrasting thermodynamic properties such as vapor pressure, heat capacity, and thermal conductivity.^4^ The two features, stability and distinguishable properties, make these allotropic forms recognized as isomers, although their chemical structures are precisely the same.

Herein, we shed light on the electronic counterpart of spin isomers, which share the same chemical structure but differ in their electronic spin orientation (Figure 1b). Electronic spin isomers (ESIs) would exhibit spin-state-dependent electronic properties and chemical reactivity, which are mostly innocent for nuclear spin isomers. The features of spin bistability^5^ and distinguishable properties are fundamental criteria for ESIs, similar to those for nuclear spin isomers (NSIs). In this work, we demonstrate that an organic diradical system can meet the two criteria of ESIs. The two spin states of a diradical, singlet (*S* = 0) and triplet (*S* = 1) states, are best described as spin-flipped combinations of the two radical units, being the electronic counterparts of NSIs.

Because organic diradicals exhibit characteristic small and tunable spin-state gaps, and the spin-flip is a spin-forbidden process, many diradicals meet the criterion of bistability.^6^ On the other hand, the property criterion is not easy to achieve. For most π-electronic systems with a large Δ*E*_ST_, the lowest singlet and triplet states are energetically separated and show quite different electronic spectra.^7^ However, it is not applicable for the case of diradicals with a small Δ*E*_ST_. Many organic diradicals have been explored, but their spin states have not been distinguished by the electronic absorption spectrum, a typical electronic property. For example, 2,5-pyridilene-bridged verdazyl radical dimer **2** with *J*/*k*_B_ ∼ +30 K^8^ and perchloro-Chichibabin’s hydrocarbon **3** with *J*/*k*_B_ = −5 K^9,10^ exhibited absorption spectra identical to the twice that of the corresponding monomer. Even in the case of quarteranthene **4** with *J*/*k*_B_ = −174 K, only a small peak shift has been observed in variable temperature measurements from 183 to 303 K, where the singlet/triplet ratio changes significantly, indicating the almost identical absorption spectra of the singlet and triplet species.^11^ Specific absorption features of radical dimers that are absent in the corresponding monomers have been observed, but no relationship with the spin state has been disclosed.^12−14^

The practically spin-independent properties of weakly coupled diradicals are simply explained by the fact that they behave as two independent radicals. To achieve <10% thermal spin excitation at room temperature for a two-spin system, the singlet–triplet energy gap should be in the narrow window from −8 to +3 kJ/mol, which is on the same order of weak interactions such as the CH/π interaction (∼6 kJ/mol)^15^ and even smaller than the C–C bond rotation barrier of the ethane molecule (Δ*E*^‡^ ∼ 12 kJ/mol). It should be noted that spin-state-dependent properties of diradicals had been observed at high-energy photoexcited states such as spin-state-selective photoinduced charge transfer,^16^ excimer-like emission,^17^ and magnetoluminescence.^18,19^ However, it is still elusive to arise from contrasting properties between energetically close spin states under ambient conditions.

## Results and Discussion

### Synthesis and Characterization

**Figure 2:**
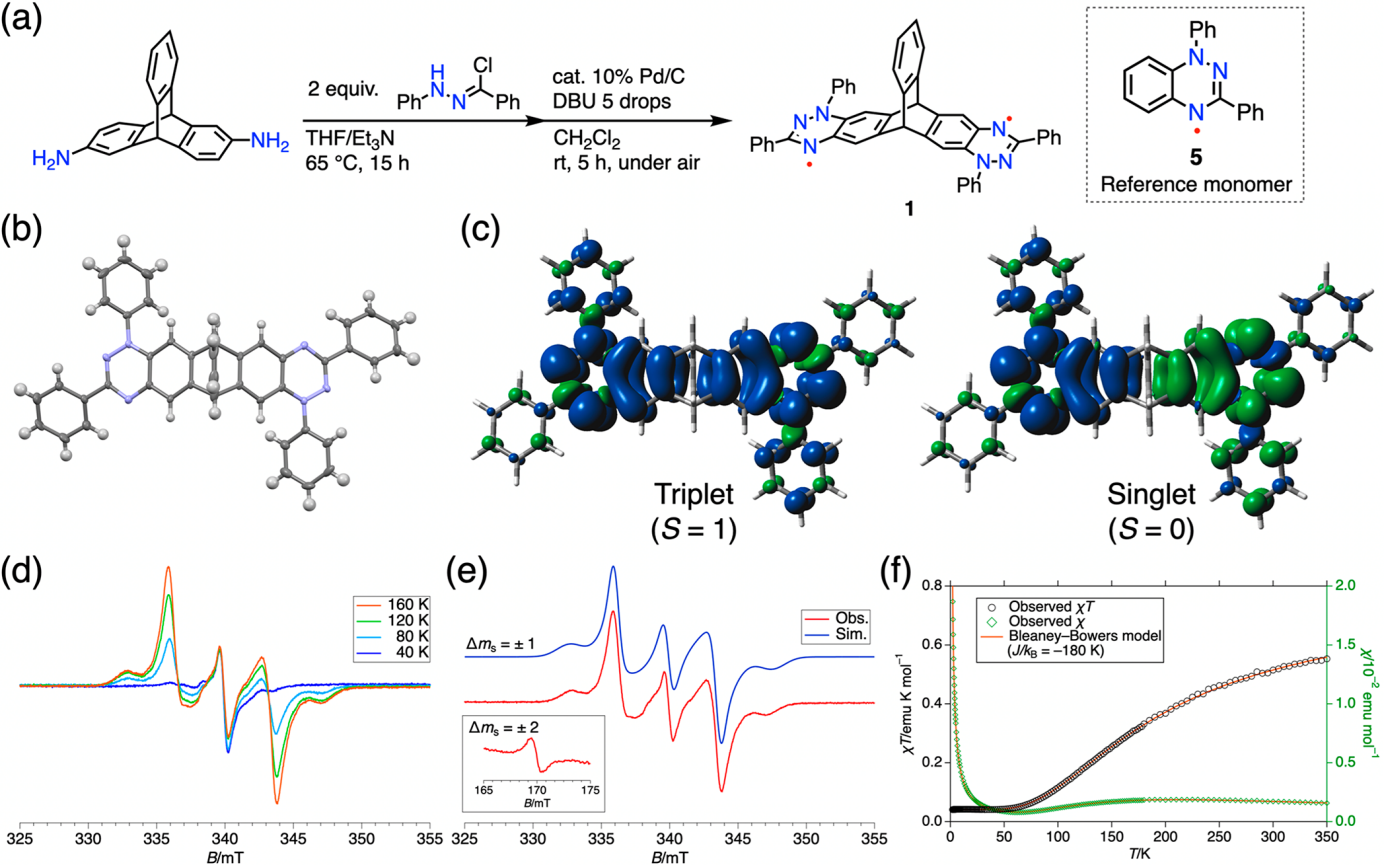
(a) Synthesis of **1** and the structure of reference monomer **5**. (b) X-ray crystal structure of **1**. Thermal ellipsoids were scaled at the 50% probability level. Solvent molecules are omitted for clarity. (c) Calculated spin density distribution plot of **1** in singlet and triplet states (isovalue: 0.001; positive and negative densities are shown in blue/green colors). (d) VT-EPR spectrum of **1** in toluene. (e) Observed (black) and simulated (red) X-band EPR spectrum of **1** in toluene recorded at 160 K. (f) Observed (circles) and fitted (line) χ–*T* and χ*T*–*T* curves of **1** observed under a magnetic field of 0.5 T.

We employed Koutentis’s method for preparing **1**, which can efficiently construct a 1,2,4-benzotriazinyl (Blatter radical) structure from aromatic amines (Figure 2a).^20^ Namely, 2,6-diaminotriptycene^21^ was reacted with *N*-phenylbenzohydrazonoyl chloride,^22^ and the following treatment with Pd/C gave Blatter radical dimer **1** in 18% yield. HRMS measurement found a molecular cation peak at *m*/*z* = 666.2519 (calcd. 666.2526 for [M]^+^, Figure S1) along with a dication peak at *m*/*z* = 333.1261 (calcd. 333.1260 for [M]^2+^, Figure S2). The ring-closure reaction occurred exclusively at the 3-position of triptycene away from the bridgehead. Diradical **1** was stable enough to be purified by conventional silica-gel column chromatography and handled under ambient conditions. No decomposition of **1** was observed in air-saturated toluene at room temperature for 48 h (Figure S3). The high stability of **1** was also confirmed by thermogravimetry, showing a 5% weight loss temperature *T*_5%_ of 338 °C (611 K), much higher than that of the Blatter radical monomer (*T*_5%_ = 221 °C (494 K), see Figures S4 and S5).

Because neutral **1** was an NMR-silent species, the structure of **1** was confirmed by the NMR spectrum of the corresponding two-electron oxidized dication **1**^2+^·2[SbF_6_]^−^ in situ generated with AgSbF_6_ (Figures S6 and S7). Single crystal X-ray diffraction analysis unambiguously determined the structure of **1** (Figure 2b, Figures S8–S12). Crystallographically the *C*_2_-symmetric structure of **1** was found in the space group of *C*_2_. The triptycene skeleton retains 3-fold symmetry as evidenced by the dihedral angle between the mean planes of each benzene ring of the triptycene moiety being 120.7°, 120.7°, and 118.7°.

### Singlet and Triplet States of **1**

The EPR spectrum of **1** in toluene showed an isotropic signal at *g* ∼ 2 below 40 K, and side bands emerged upon heating to 160 K (Figure 2d). Therefore, we assigned the central isotropic signal to the paramagnetic impurity and the side signals to the thermally excited triplet state of **1**.^23^ The EPR spectrum recorded at 160 K showed a |Δ*m*_s_| = 1 signal at *g* = (2.0047, 2.0033, 2.0041) and a |Δ*m*_s_| = 2 signal at the half-field region (Figure 2e). The |Δ*m*_s_| = 1 signal was reproduced by zero-field splitting parameters of |*D*| = 208 MHz and |*E*| = 0.0 MHz, indicating an axially symmetric spin nature. The spin–spin distance was estimated to be 6.3 Å based on the |*D*| in point dipole approximation, which is compatible with the 6.6 Å separation between the centroids of the benzotriazinyl moieties. The triplet signal diminished upon cooling, showing the ground-singlet nature.

The intramolecular spin–spin interaction of **1** was evaluated by SQUID magnetometry on a powder sample. The χ*T*–*T* curve of **1** showed a plateau close to zero at 2–50 K and increased above 50 K, indicating the ground singlet nature and thermal population to the triplet state. The experimental χ*T*–*T* curve of **1** was reproduced by the Bleaney–Bowers model^24^ with *J*/*k*_B_ = −180 K, which corresponds to the singlet–triplet energy gap (Δ*E*_ST_ = 2*J*) of −3.0 kJ/mol (Figure 2f, see Figure S13 for the model equation). The exchange interaction was also supported by the VT-EPR measurement, showing *J*/*k*_B_ = −177 K (Figure S14). The experimental Δ*E*_ST_ is consistent with theoretical calculation, predicting Δ*E*_ST_ = −1.6 kJ/mol at the RAS(2,2)-SF/def2-TZVP level.

The spin density distribution indicates that **1** has an open-shell electronic structure in which two Blatter radicals interact antiferromagnetically (Figures 2c,d). CASSCF(2,2)/6-311G* calculation showed the diradical index *y* of **1** as high as 89% in the singlet ground state (Table S6). Due to the rigid triptycene backbone, the structural difference between the two spin states is minimal, as indicated by geometrical optimization (Figure S28). Thus, the lowest singlet and triplet states of **1** are best described as electronic spin isomers with spin-flipped electronic structures.

### Steady-State Optical Properties

**Figure 3:**
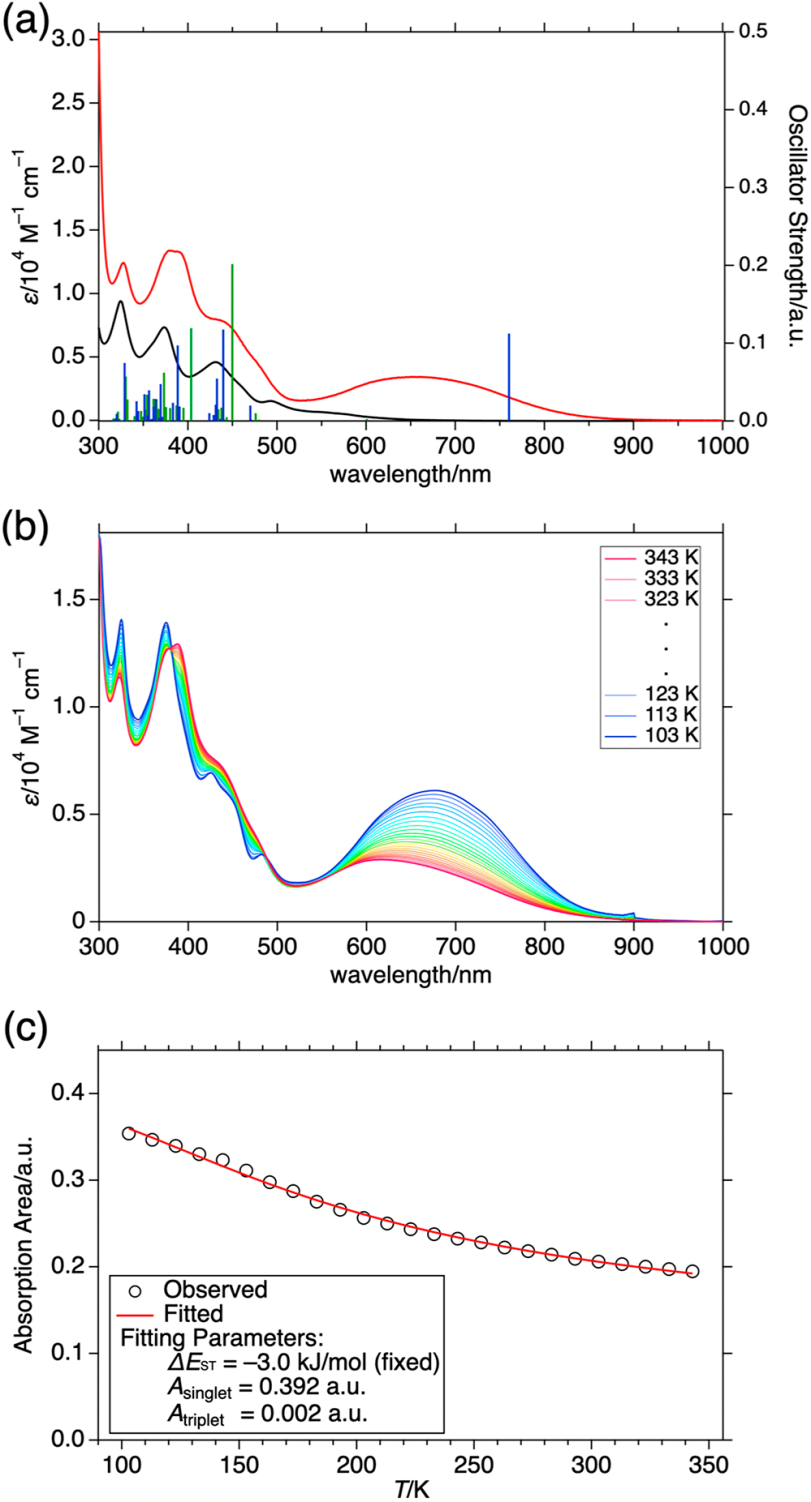
(a) Absorption spectra of monomer **5** (black) and diradical **1** (red) in toluene at room temperature. Blue and green bars represent the predicted excitation energy and oscillator strength obtained by TD-DFT calculation. (b) Temperature dependence of the absorption spectra of **1** in 2-MeTHF. (c) Temperature dependence of the absorption area of **1** below 2.2 eV (563 nm < λ).

**Figure 4:**
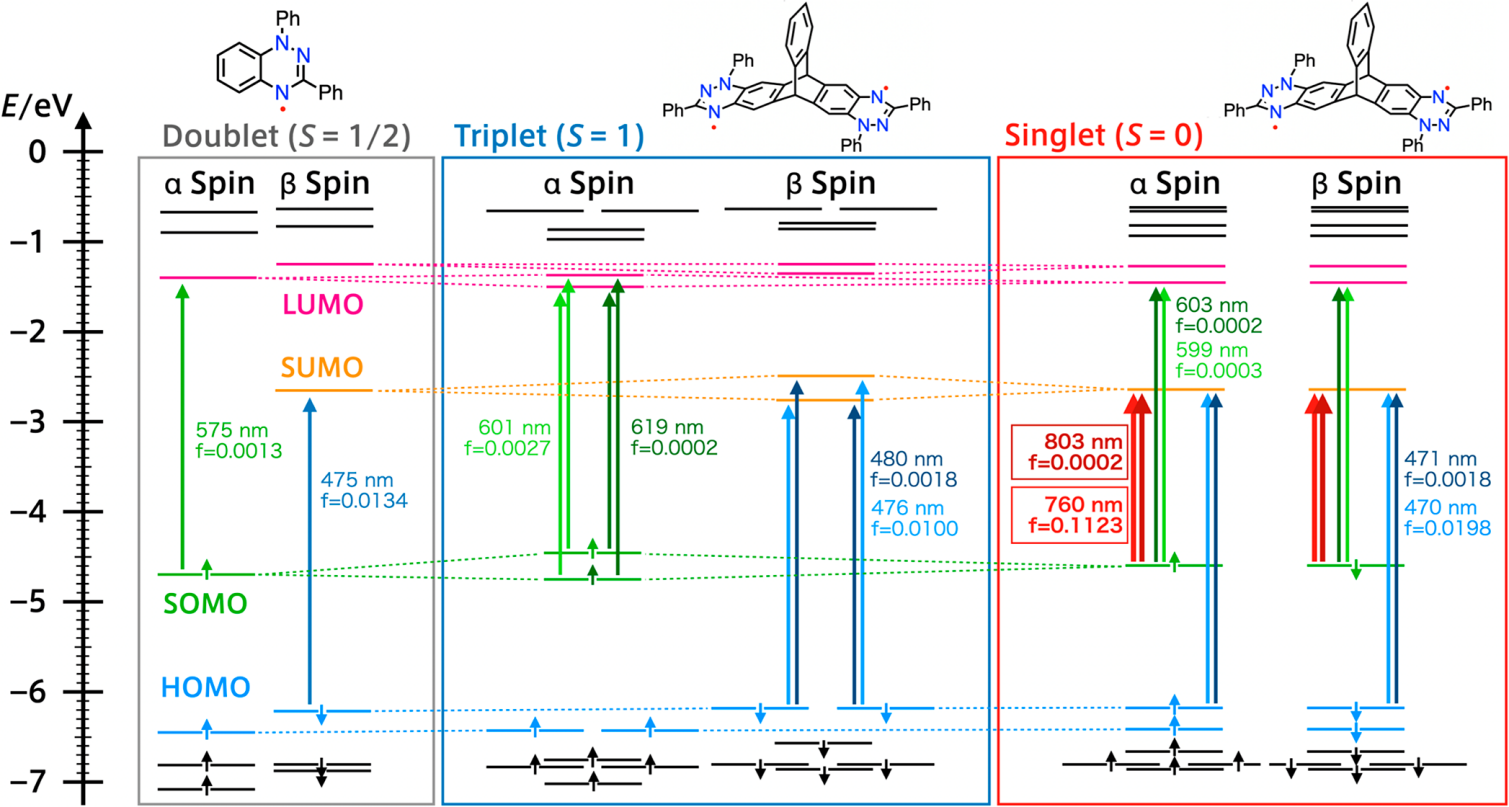
MO diagrams and calculated electronic transitions of **5** (doublet, left), **1** (triplet, middle), and **1** (singlet, right) calculated at the UB3LYP/6-311G* level. For **1**, two one-electron transitions of the same color compose each transition.

Figure 3a shows the electronic absorption spectra of **1** in toluene at 298 K. Compared to Blatter radical monomer **5**, diradical **1** exhibited a spectrum almost twice as large as that of the monomer in the region below 500 nm. On the other hand, characteristic NIR absorption bands were found at 650 nm with ε = 3.4 × 10^3^ cm^–1^ M^–1^. The thermally grown triplet band in VT-EPR spectra and SQUID magnetometry indicated that **1** has a singlet ground state with a thermally accessible triplet state of 3.0 kJ/mol higher energy (Δ*E*_ST_/*k*_B_ = −360 K). According to the Boltzmann distribution and experimental Δ*E*_ST_ (−3.0 kJ/mol), the population of triplet species was estimated to be almost half (47%) at 298 K. Therefore, we estimated excitation spectra from both the lowest singlet and triplet states by the TD-DFT method (TD-UB3LYP/6-311G*). The obtained excitation energy and oscillator strengths are summarized in Table 1 and Figure 4 (Tables S13–S18).

**Table 1:** Wavelength and Oscillator Strengths of the Predicted Lowest and Second-Lowest Energy excitations

	spin state	λ_abs_	oscillator Strength (*f*)	assignment (contribution)
**5**	doublet	575 nm	0.0013	SO(α)→LU(α) (90%)
		475 nm	0.0134	SO–1(α)→LU(α) (74%)
1	singlet	803 nm	0.0002	SO(α)→SU(α) (50%); SO(β)→SU(β) (50%)
		760 nm	0.1123	SO(α)→SU(α) (50%);^a^ SO(β)→SU(β) (50%)^a^
**1**	triplet	619 nm	0.0002	SO(α)→LU(α) (72%)
		601 nm	0.0027	SO(α)→LU+1(α) (72%); SO–1(α)→LU(α) (38%)

a The two excitations for **1** in the singlet state (803 and 760 nm) are assigned to the same sets of transitions, but they are opposite in the sign of the coefficient (see also Table S15). SO = SOMO, SU = SUMO, LU = LUMO.

Surprisingly, the calculation predicted that the characteristic NIR absorption of **1** is purely attributed to the excitation from the singlet state, which means that we can selectively photoexcite one of the two mixed states in a solution. The two calculated NIR excitations of **1** are the consequence of the configuration interaction between the SOMO(α) → SUMO(α) and SOMO(β) → SUMO(β) transitions. To confirm the theoretical conjecture, we measured the temperature dependence of absorption spectra of **1** in 2-MeTHF. While monomer **5** shows almost no temperature dependence (Figure S15), a substantial spectral change was recorded for **1** with isosbestic points around 380 and 485 nm. The absorption band at 500–900 nm clearly diminishes as temperature elevates. This behavior of **1** is consistent with a decreasing singlet population at higher temperatures. The trend continues below the melting point of the solvent (137 K), indicating the spectral change is not due to structural dynamics. This is also supported by the homogeneity of the optimized structure at each spin state (Figure S28).

The extinction coefficient (absorption area) of the absorption band of **1** below 2.2 eV (563 nm) was estimated by fitting the absorption area, which is proportional to the transition dipole moment, showing that the singlet state has an almost 200 times larger extinction coefficient (Figure 3c, see also Figures S16–S18 and associated discussion in the SI). A similar temperature-dependent spectral change was also observed in toluene (Figure S19). The solvent dependence of the lowest energy absorption band of **1** was marginal (1.74 eV in toluene and 1.69 eV in DMSO), showing the non-CT character of the Franck–Condon states (Figures S20 and S21 and Table S3). The non-CT character was also supported by the TD-DFT calculations including solvent polarity with the IEFPCM model (Table S4).

According to TD-DFT calculations, the major transitions for the singlet-specific band are SOMO(α) → SUMO(α) and SOMO(β) → SUMO(β) transitions. The SOMO(α) and SUMO(β) are localized on one of two Blatter radical units, and the SOMO(β) and SUMO(α) are localized on the other Blatter radical unit (Figures S35 and S37). Therefore, the singlet-specific band has an electron exchange nature. On the other hand, the lowest-energy excitation for the triplet state was a complex mixture of transitions. However, hole–electron (h–e) analysis clearly indicates the net transition of the sum of excitation of each radical unit, as judged from the homology of the h–e map.

### Excited-State Dynamics

**Figure 5:**
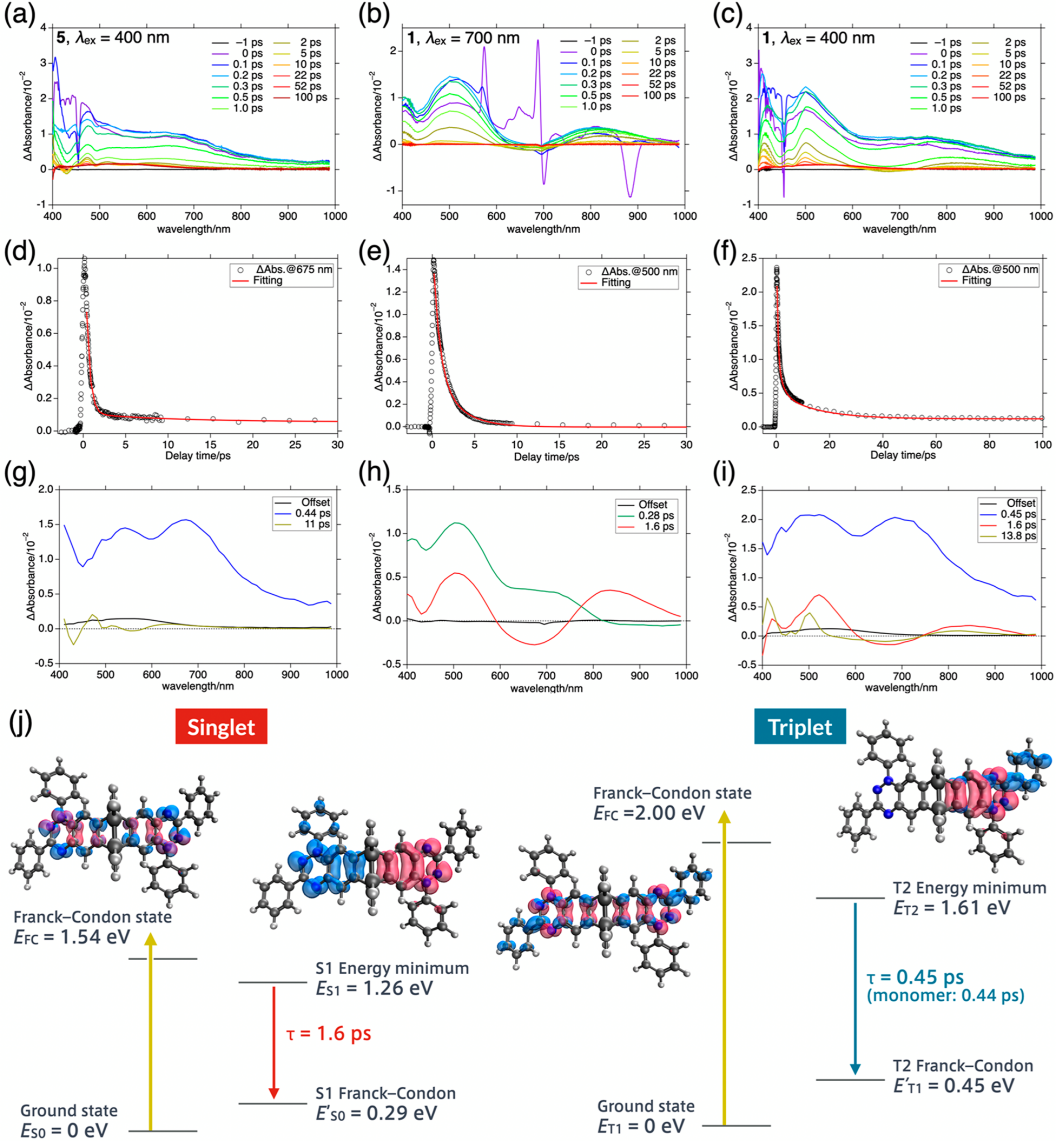
Transient absorption spectrum of (a) **5** (λ_ex_ = 400 nm), (b) **1** (λ_ex_ = 700 nm), and (c) **1** (λ_ex_ = 400 nm). TA decay profiles: (d) **5** (λ_ex_ = 400 nm), (e) **1** (λ_ex_ = 700 nm), and (f) **1** (λ_ex_ = 400 nm). Decay-associated spectra: (g) **5** (λ_ex_ = 400 nm), (h) **1** (λ_ex_ = 700 nm), and (i) **1** (λ_ex_ = 400 nm). The offset component is an artifact due to the excimer formation of the toluene solvent induced by off-resonant two-photon absorption. (j) Schematic drawing of excited state dynamics of **1** in the singlet and triplet states. Red/blue cubes represent calculated hole/electron distribution at each excited state.

We also investigated the excited-state dynamics of **1** by transient absorption (TA) spectroscopic studies. TA spectra of reference monomer **5** were measured upon excitation at 400 nm (Figure 5a). Upon excitation at 400 nm, monomer **5** showed a two-component decay process with time constants of 0.44 and 11 ps (Figure 5d), which were assigned as the lifetime of the D_1_ state and vibrational cooling after deactivation into the D_0_ state, respectively. The decay-associated spectrum (DAS) of the first time constant (0.44 ps) has two characteristic bands at around 550 and 700 nm (Figure 5g). For dimer **1**, we conducted TA measurements with pump pulses of 700 and 400 nm, which were assigned to the singlet-specific and nonspecific bands, respectively (Figure 5b,c). The TA profile of **1** upon excitation at 700 nm was characterized by two decay components of 0.28 and 1.6 ps (Figure 5e). The former was assigned to the time constant of relaxation from the initially populated Franck–Condon state, and the latter to the lifetime of the S_1_ excited state. The DAS of the second component (1.6 ps) has bands at 500 and 800 nm in addition to a ground-state bleaching band at around 650 nm (Figure 5h).

The photoirradiation of **1** at 400 nm brought more complex dynamics. Assuming that the two spin states follow individual decay pathways, we conducted global fitting by fixing the time constants of 0.28 and 1.6 ps obtained by photoexcitation at 700 nm (Figure 5f). The decay profile was fitted with additional components with time constants of 0.45 and 13.8 ps. DAS shows the spectral homology of the 1.6 ps component of excited species between the spectra obtained upon photoexcitation at 400 and 700 nm (Figure 5i). Thus, we assigned the species with 1.6 and 0.45 ps to the S_1_ and T_2_ excited states of **1**, respectively. Intersystem crossing (spin-state interconversion) was not observed in the lifetime, and singlet and triplet states follow independent decay pathways. Notably, the DAS and lifetime of the T_2_ state of **1** resemble that of the D_1_ state of monomer **5** (Table 2).^25^ It is also intriguing that the lifetime of the S_1_ state (1.6 ps) is much longer than that of the T_2_ state (0.45 ps), although the energy gap between the S_1_ and S_0_ states should be larger than that between the T_2_ and T_1_ states.

**Table 2:** Summary of Decay Profiles of **1** Measured in Toluene

	λ_ex_	lifetime (amplitude^b^)	assignment
**5**	400 nm	0.44 ps (96%)	D_1_ excited state
		11 ps (4%)	vibrational cooling
**1**	700 nm	0.28 ps (61%)	internal conversion
		1.6 ps (39%)	S_1_ excited state
**1**	400 nm	0.28 ps^a^ (15%)	internal conversion
		1.6 ps^a^ (22%)	S_1_ excited state
		0.45 ps (49%)	T_2_ excited state
		13.8 ps (13%)	vibrational cooling

a Fixed in the fitting.

b Monitored at 675 nm for **5** and 500 nm for **1**.

To explore the excited-state dynamics of **1**, we calculated the energy-minimized geometry of **1** at the first excited singlet and triplet states at the TD-UB3LYP/6-311G* level. Based on the obtained excited-state geometry, an h-e analysis of each state was conducted to visualize the electronic structure at the excited states (Figure 5j). At the S_1_ state, the hole and electron are localized in each of the two radical units, indicating the symmetry-breaking intramolecular charge transfer (SBCT) nature of the S_1_ state. Therefore, the lifetime of 0.28 ps can be attributed to the time scale of the symmetry-breaking charge separation. The SBCT mechanism was experimentally supported by the solvent polarity dependence of the excited-state lifetime. The excited-state lifetime of **1** became shorter down to 0.35ps in acetone as the polarity of the solvent increased (see also Figures S22–S24). This is consistent with the SBCT mechanism, in which the energy level of the CT state is lowered as the solvent polarity increases, leading to faster relaxation to the ground state. The S_1_-optimized structure of **1** shows significant structural relaxation from the ground state structure, while the T_2_-optimized structure shows less change from the T_1_ state (Figures S31 and S32). On the other hand, in the T_2_ state, both hole and electron are localized on one of the two radical units. The localization leads to the monomer-like excited state, which explains the homology of the DAS and the lifetime of the T_2_ state of **1** and the D_1_ state of **5**. Such singlet-specific photoinduced electron transfer was observed for the diradical dianion of perylenebisimide dimers.^16^ These results indicate that we can selectively photoexcite one of the two coexisting spin states.

### Electrochemistry

**Figure 6:**
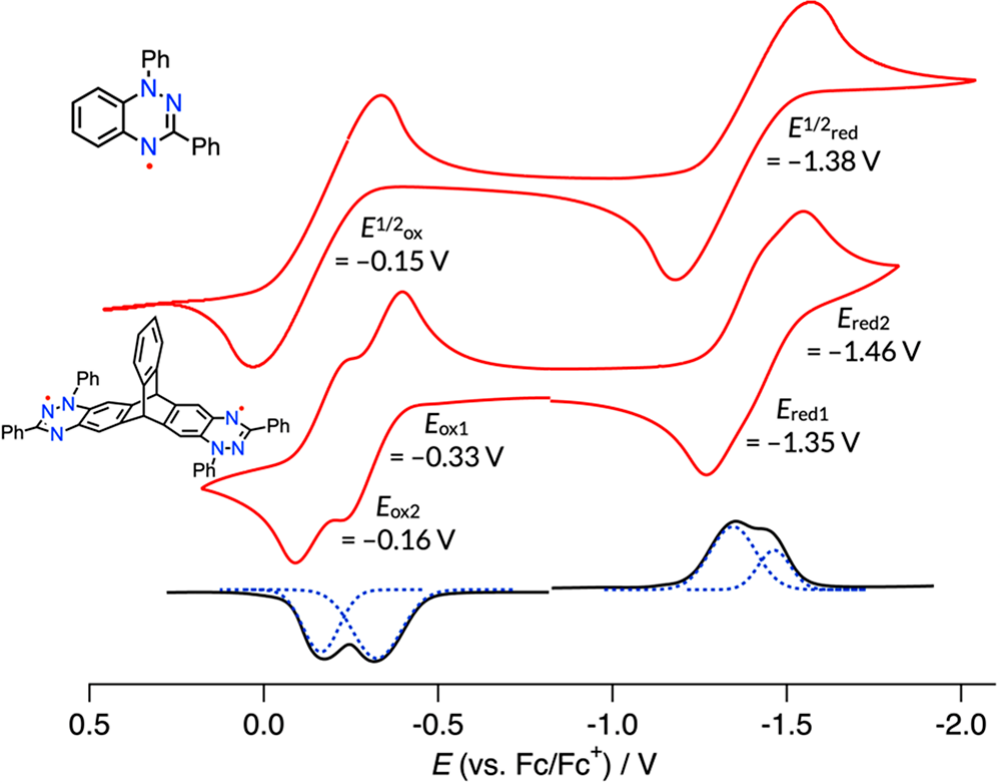
Cyclic voltammogram (red) of monomer **5** and dimer **1** and differential pulse voltammogram of **1** (black). Solvent: CH_2_Cl_2_. Supporting electrolyte: 0.1 M *n*Bu_4_NPF_6_. Reference electrode: Ag/AgNO_3_ in MeCN. Working/counter electrodes: Pt/Pt wire. Scan rate: 0.05 V/s. Redox potentials of **1** were determined by Gauss fitting of the DPV curve (dashed line).

To investigate the predicted symmetry-breaking intramolecular charge transfer, in which one electron is transferred from one radical unit to the other, electrochemical studies of diradical **1** were performed by using cyclic voltammetry (CV) and differential pulse voltammetry (DPV) in CH_2_Cl_2_ (Figure 6). Fairly reversible two 1e-oxidation waves and two 1e-reduction waves were observed. Redox potentials were determined to be *E*_ox_ = −0.33 and −0.16 V and *E*_red_ = −1.35 and −1.46 V (vs Fc/Fc^+^) by deconvolution analysis on the differential pulse voltammogram. The small split of redox waves of 0.17 V (oxidation) and 0.11 V (reduction) suggests a small interchromophore interaction in **1**.

From the redox potential, we roughly estimated the SBCT state according to the Rehm–Weller equation:
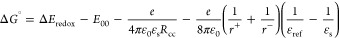
where Δ*E*_redox_ is the redox potential gap of the radical unit, *E*_00_ signifies the spectroscopic excitation energy (corresponding to the Franck–Condon state energy), and *R*_cc_ is the distance between the centers of the donor and acceptor moieties.^26^ We adopted *R*_cc_ of 6.3 Å from the experimental |*D*| in the EPR study, *E*_00_ of 1.74 eV from the absorption spectrum (Table S3), and *r*^+^ = *r*^–^ = 2.5 Å. The CT state was estimated to be only 0.66 eV above the Franck–Condon (FC) state, even in apolar toluene (ε = 2.4), and the excited-state charge separation was predicted to be energetically feasible in solvents with ε > 4.5 (Figure S22). In fact, TD-DFT optimization of the S_1_ state geometry from the FC (ground state) structure falls into the CT state even under vacuum conditions. Therefore, we consider that the SBCT occurs even in toluene with the aid of structural relaxation. This explains why the singlet excited state has a lifetime longer than the triplet state, seemingly violating the energy gap rule.

### Origin of the Spin-State Specific Photophysical Responses

TD-DFT results showed that the singlet-specific band of **1** is assigned to the SOMO–SUMO transitions which are spin forbidden for the triplet state. Therefore, it should be a general phenomenon that a diradical has a characteristic absorption band exclusively for the singlet state. However, as discussed in the Introduction, many diradical species show absorption spectra with no spin-state dependence. In principle, a transition dipole moment is expressed as the following equation:
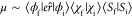
where the first term represents the integral of the electronic wave function.^27^ In the case of a diradical’s SOMO–SUMO transition, the SOMO and SUMO are localized in separate radical units. Therefore, overlapping of SOMOs is essential to making the transition allowed. On the other hand, the overlap integral of the two SOMOs appears in the expression of Δ*E*_ST_:
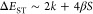
where *k* is the exchange integral, β is the resonance integral, and *S* is the overlap integral.^28^ These formulations indicate that the overlap of the two SOMOs is crucial for the transition probability, but a larger overlap increases the energy gap between the singlet and triplet spin states. To keep Δ*E*_ST_ as small as the thermal energy, orbital overlap (conjugative interaction) between the SOMOs must be minimized. Consequently, on most diradicals with small Δ*E*_ST_, interunit charge transfer transitions become forbidden by small overlap, leaving only intraunit (monomer-like) transitions. Thus, the absorption spectrum looks like the sum of the spectrum of each radical unit with no spin-state dependence. For example, the Δ*E*_ST_ of quateranthene **4** (−3.0 kJ/mol) is consistent with that of **1** (−3.0 kJ/mol), but the spin-specific absorption band was observed only for **1**. In molecule **1**, π-conjugation is disconnected by the sp^3^ carbons of the triptycene skeleton, so the interunit interaction is mainly attributed to through-space interaction. This through-space conjugation was very effective in not only decreasing Δ*E*_ST_ but also achieving the interunit charge transfer transitions. This work demonstrates that through-space interaction is a way to overcome the drawback of meeting both a thermally accessible spin-state gap and a practically allowed spin-specific SOMO–SUMO transition.

## Conclusion

In summary, we synthesized and characterized through-space-conjugated diradical **1**, demonstrating a novel model of electronic spin isomers (ESIs). SQUID magnetometry and VT-EPR studies on **1** showed a small singlet/triplet spin-state energy gap of −3.0 kJ/mol, leading to ca. 1:1 coexistence of the two spin states at room temperature. Diradical **1** shows a characteristic electronic absorption band in the NIR region, which was solely attributed to **1** in the singlet state. The spin-specific absorption band allows us to distinguish two spin states by steady-state absorption and selectively photoexcite the singlet state of **1**. Excited-state dynamics of each spin state were monitored by ultrafast transient absorption spectroscopy, which revealed qualitatively different excited dynamics and supported spin-specific excitation at the NIR band. The origin of the spin-specific absorption band was the SOMO–SUMO electron exchange transition between the radical units. This work demonstrates that the through-space approach is crucial to balancing the overlap integral of the two radical units to both meet a small spin-state energy gap and allow SOMO–SUMO electronic transition. We first demonstrate and rationalize the idea of diradical-based ESIs, but we consider that this is not the first case. It means many potential ESIs and spin-dependent properties of diradicals have been overlooked. Therefore, there is plenty of room to revisit previously investigated multiradical systems, including supramolecular radical assemblies, from the viewpoint of ESIs. The drawback of the present system is that only one of the two spin states can be selectively photoexcited, and the excitation lifetime is relatively short. Further investigation on multiradical systems, such as establishing molecular design guidelines, controlling spin-state interconversion, and dual/selective emission from multiple spin states, are ongoing in our laboratories.

## Experimental Section

### Synthesis of Diradical **1**

2,6-Diaminotriptycene (142 mg, 0.5 mmol)^29^ and *N*-phenylbenzohydrazonoyl chloride (230 mg, 1.0 mmol, 2 equiv)^30^ were placed in a round-bottom flask under an atmosphere of N_2_. To the mixture were added THF (3 mL) and Et_3_N (0.2 mL), and the resulting mixture was stirred at 65 °C. After 15 h, the reaction mixture was concentrated under reduced pressure. The residue was dissolved in CH_2_Cl_2_ (3 mL), and 10% Pd/C (60 mg) and DBU (0.25 mL, 1.6 mmol) were added to the solution. The mixture was stirred under open air at room temperature. After 5 h, the suspension was filtered through a short Celite pad, and the filtrate was collected and concentrated under reduced pressure. The crude product was purified by column chromatography (eluent: *n*-hexane/CH_2_Cl_2_/EtOAc = 5/5/0 → 0/10/0 → 0/9/1). Recrystallization from CH_2_Cl_2_/MeOH afforded **1** as a green solid (59.5 mg, 0.89 mmol, 18%). No unexpected or unusually high safety hazards were encountered throughout the synthesis and handling for measurements.

^1^H NMR (500 MHz, CD_2_Cl_2_ containing 3 equiv AgSbF_6_, 298 K): δ 8.67 (s, 2H), 8.58 (dd, *J* = 6.5, 1.5 Hz, 4H), 8.29 (s, 2H), 7.97 (tt, *J* = 7.3, 1.3 Hz, 2H), 7.89 (t, *J* = 8 Hz, 4H), 7.86–7.82 (m, 6H), 7.77 (tt, 7.3, 1.3 Hz, 2H), 7.69 (t, *J* = 8 Hz), 7.47 (dd, *J* = 5.5, 3 Hz, 2H), 6.36 (s, 2H). ^13^C NMR (126 MHz, CD_2_Cl_2_ containing 3 equiv AgSbF_6_, 298 K): δ 163.0, 154.1, 153.0, 152.4, 141.2, 138.4, 136.3, 134.3, 134.2, 132.7, 131.2, 129.8, 129.3, 126.7, 126.6, 126.5, 126.4, 116.8, 52.9. HR-ESI-orbitrap-MS calculated: *m*/*z* 666.2526 for [C_46_H_30_N_6_]^+^, [M]^+^, and 333.1260 for [M]^2+^. Observed: *m*/*z* 666.2519, 333.1261.

## Supporting Information Available

NMR, HRMS, thermogravimetry, magnetometry, optical, electrochemical data, and calculation results (PDF)
Transparent Peer Review report available (PDF)
