# Supplementary material for: Optically Distinguishable Electronic Spin-isomers of a Stable Organic Diradical
Source: ACS Cent Sci. 2024 Apr 8;10(4):890–8. doi: 10.1021/acscentsci.4c00284 (PMC11046471; doi:10.1021/acscentsci.4c00284)
Supplement: Supplementary file 1 — oc4c00284_si_001.pdf [file oc4c00284_si_001.pdf]

Supporting Information

Optically Distinguishable Electronic Spin-isomers of a Stable Organic Diradical

Daiki Shimizu,[1,*] Hikaru Sotome,[2] Hiroshi Miyasaka,[2] and Kenji Matsuda[1,3,*]

[1]*Department of Synthetic Chemistry and Biological Chemistry, Graduate School of Engineering, Kyoto University, Nishikyo-ku, Kyoto 615-8510, Japan*

[2]*Division of Frontier Materials Science and Center for Promotion of Advanced Interdisciplinary Research, Graduate School of Engineering Science, Osaka University, Toyonaka, Osaka 560-8531, Japan*

[3]*Fukui Institute for Fundamental Chemistry, Kyoto University, Sakyo-ku, Kyoto 606-8103, Japan.*

**Email: dshimizu@sbchem.kyoto-u.ac.jp.*

Contents

1. Instrumentation and Materials
2. HRMS Data
3. Stability Studies
4. NMR Spectra
5. X-Ray Crystallographic Data
6. Magnetometry Data
7. EPR Spectra
8. Optical Spectra
9. Electrochemistry
10. Quantum Chemical Calculation Results
11. Supporting References

1. Instrumentation and Materials

A. Materials. All reagents and solvents were of commercial reagent grade and were used without further purification unless where noted. Preparative separations were performed by silica gel column chromatography (Biotage Isolera One with Rening cartridges). ^1H and ^{13}C NMR spectra of **1** in CD_2Cl_2 were recorded on a JEOL ECZ500 spectrometer, and chemical shifts were reported relative to residual solvents as an internal standard (δ = 5.32 ppm for ^1H and 5.32 ppm 53.8 ppm for ^{13}C). CD_2Cl_2 for NMR spectroscopy was purified by passing through a short aluminum oxide pad before use.

B. Steady-State Studies. The X-band EPR spectrum of **1** was recorded on a Bruker EMX plus spectrometer equipped with an Oxford EPR900 cryostat. The spectral simulation was carried out using the EasySpin toolbox (5.2.35) with the MATLAB program.[31] Magnetometry was performed with a Quantum Design MPMS instrument. UV-Vis-NIR absorption spectra were recorded on a JASCO V-670 spectrometer equipped with a UNISOKU CoolSpeK USP-203 cryostat. Spectroscopic grade solvents were used for all spectroscopic studies without further purification. Spectral fitting was performed using the Fityk program.[32] HR-ESI-orbitrap mass spectra were recorded on a Thermo Fisher Scientific EXACTIVE Plus mass analyzer. X-Ray crystallographic data were recorded using a Rigaku MicroMax 007-HF diffractometer equipped with a graphite monochromatic MoKα radiation source (λ = 0.71075 Å) and a Rigaku Saturn724+ CCD detector. The structures were solved using direct methods (SHELXT-2014/5),[33] and structural refinements were carried out using SHELXL-2018/3.[34] Cyclic voltammogram and difference pulse voltammogram were measured on a BAE ALS612E electrochemical analyzer. Electrochemical potentials are determined by using the ferrocene/ferrocenium ion couple as an external standard. Thermogravimetric analyses were performed with a Rigaku TG-DTA8122. The aluminum sample pan was used for the TG-DTA study, and the scan was performed under an atmosphere of N_2 at a scan rate of 5 °C/min.

C. Time-Resolved Spectroscopy. Transient absorption spectra were measured with a home-built setup based on a Ti:sapphire regenerative amplifier (Spectra-Physics, Spitfire). The output was divided into two portions, and one was converted into a pulse at 1180 nm using an optical parametric amplifier (OPA, Light Conversion, TOPAS-Prime). This near-infrared pulse was focused on a CaF_2 plate for the generation of the white light continuum as the probe pulse. The probe pulse was further split into the signal and reference pulses, which were detected with a pair of multichannel photodiode arrays (Hamamatsu, PMA-10). The other part was converted into the excitation pulse at 700 nm with other OPA (Light Conversion TOPAS-Prime) or frequency-doubled with a beta-barium borate crystal for the photoexcitation at 400 nm. After the probe pulse passed through an optical delay stage, both the excitation and probe pulses were guided into the sample cell in the almost collinear configuration. The polarization of the excitation pulse was set to the magic angle with respect to that of the probe pulse. The sample solution was filled into a rotation cell with an optical length of 2 mm and circulated during the measurements. The chirping of the probe pulse was corrected based on an optical Kerr effect. The time resolution was ca. 150 fs.

Transient absorption measurements for solvent polarity dependence of the excited state lifetime were performed using a newly developed setup based on a Yb:KGW regenerative amplifier (Light Conversion,

Pharos). The fundamental output was split into two, and a major portion was frequency-doubled in a beta-barium borate crystal. The resultant second harmonics at 514 nm was used for photoexcitation of the sample. The other small portion was focused into a 4 mm-thick YAG window and converted into a white light continuum as the probe pulse. The detection system and other experimental conditions were the same as the setup based on the Ti:sapphire regenerative amplifier.

D. Computational Methods. (TD-)DFT and CASSCF calculations were conducted using the Gaussian 16 program (revision C.01).[35] Hole–electron analysis was done using the Multiwfn software[36] based on the Gaussian output files and visualized with the IQmol. RAS-SF calculation was performed on the Q-Chem 5.4 package[37] based on the energy-minimized molecular geometry at the UB3LYP/6-311G* level.

2. HRMS Data

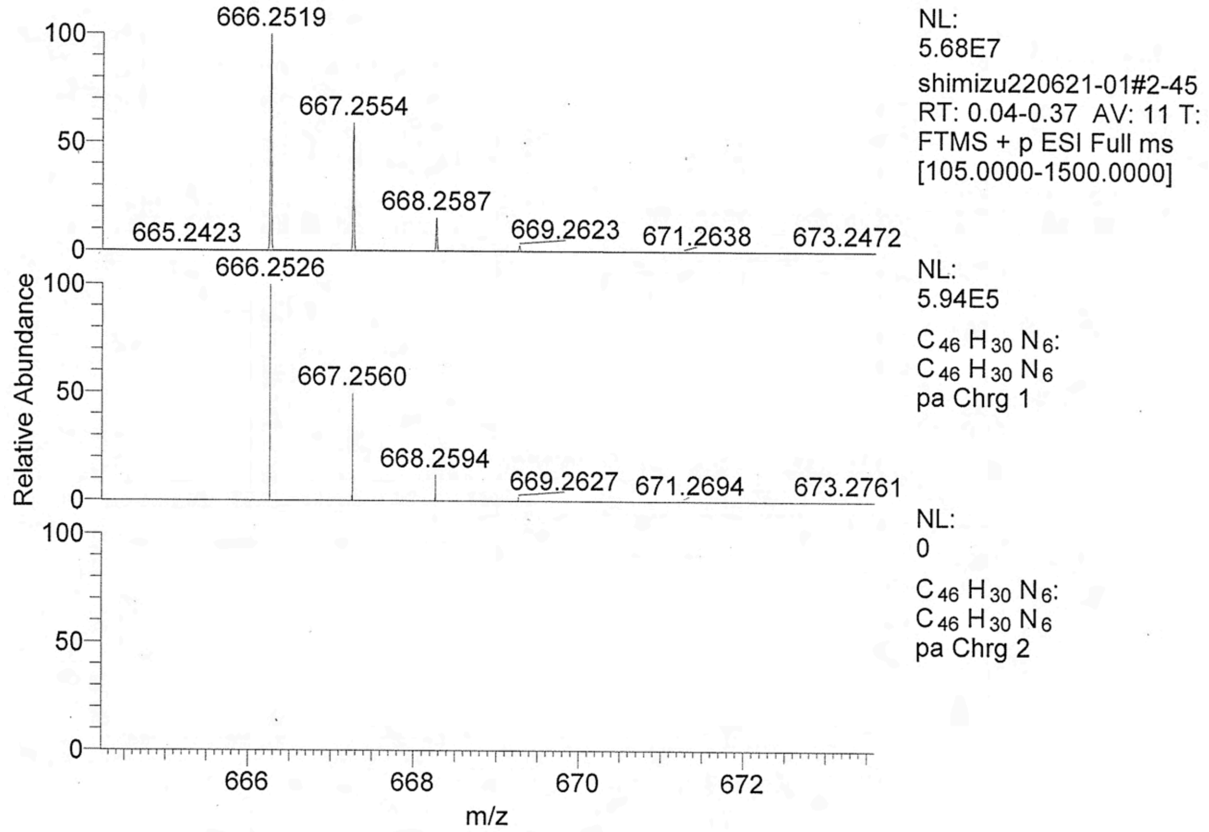

Figure S1. High-resolution mass spectra of **1** (ESI-orbitrap, positive mode).

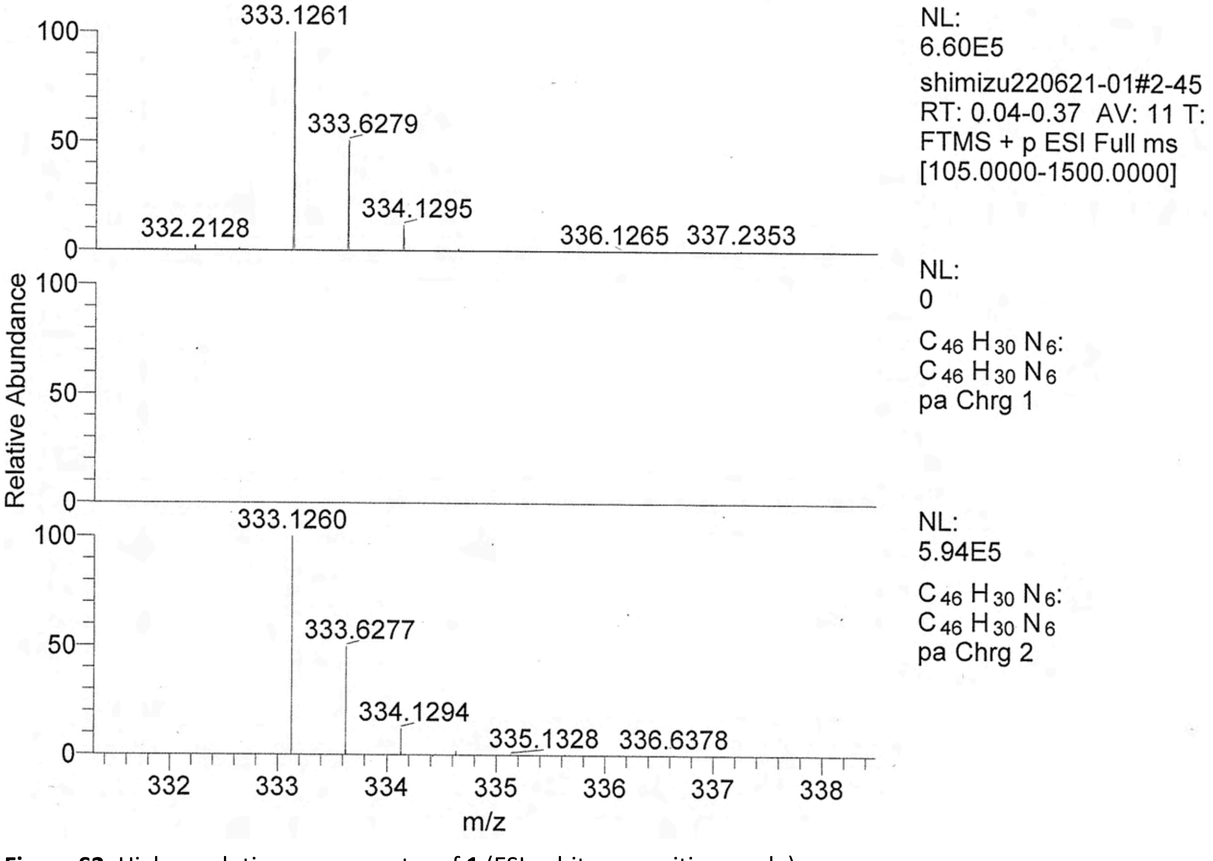

Figure S2. High-resolution mass spectra of **1** (ESI-orbitrap, positive mode).

3. Stability studies

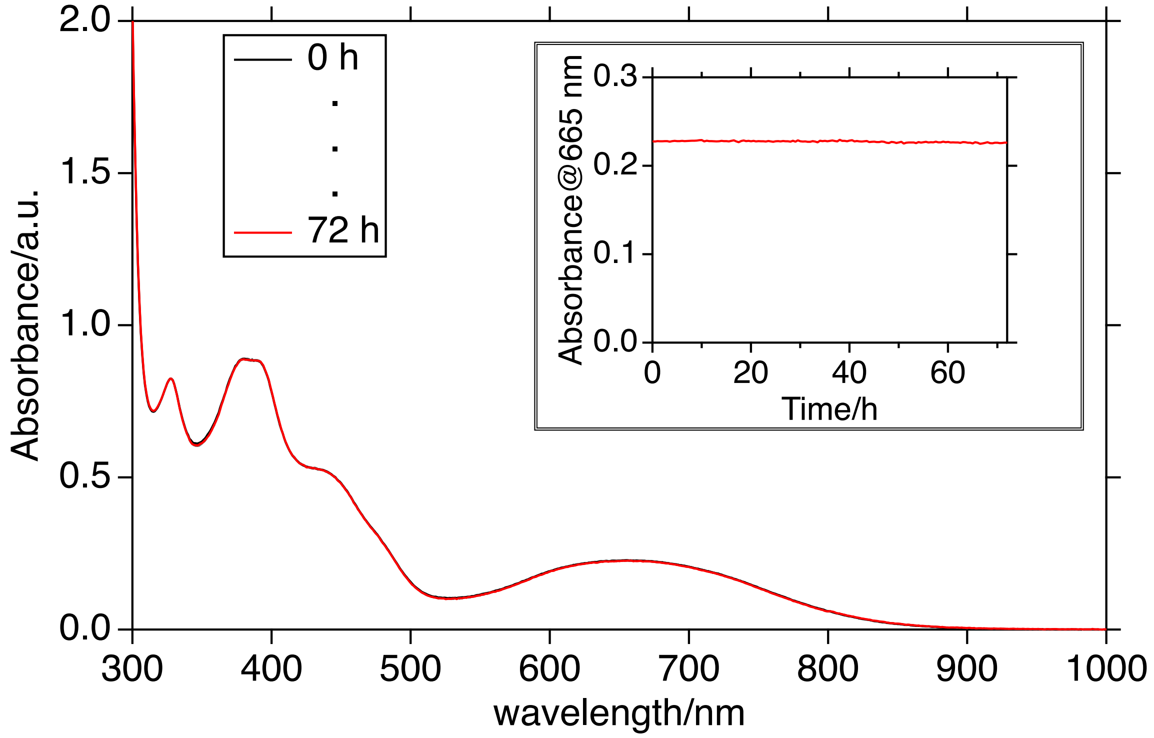

Figure S3. Spectral change of a solution of **1** in air-saturated toluene at room temperature.

Thermogravimetry. Stability of Blatter radical dimer **1** and reference monomer **6** was assessed by TG-DTA. The temperatures for the 5% ($T_{5\%}$) and 10% ($T_{10\%}$) mass loss of **6** were 221 and 238 °C, respectively. The DTA of **A** shows negative peak at 109 °C without mass loss, which corresponds to melting point. On the other hand, the $T_{5\%}$ (338 °C) and $T_{10\%}$ (350 °C) of **1** were much higher than **M**. Similarly, DTG and DTA of **1** show peaks at 351 and 353 °C, respectively. The DTA peak has a negative spike at low-temperature shoulder, which corresponds to melting of **1**. The exothermic decomposition concomitantly occurs upon melting, which facilitates intermolecular reaction.

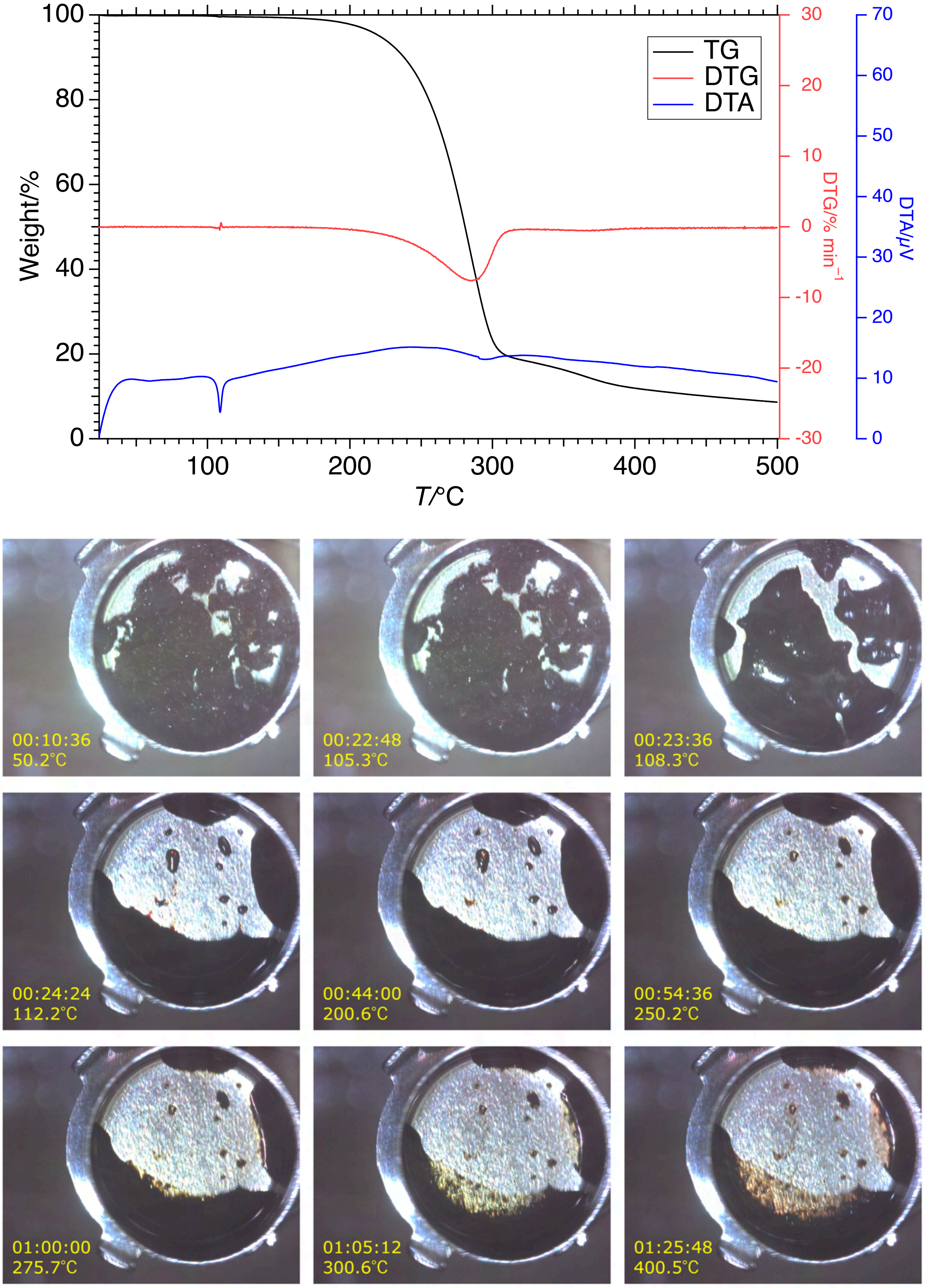

Figure S4. TG-DTA plot of **6** under and atmosphere of N_2. Scan rate: 5 °C/min.

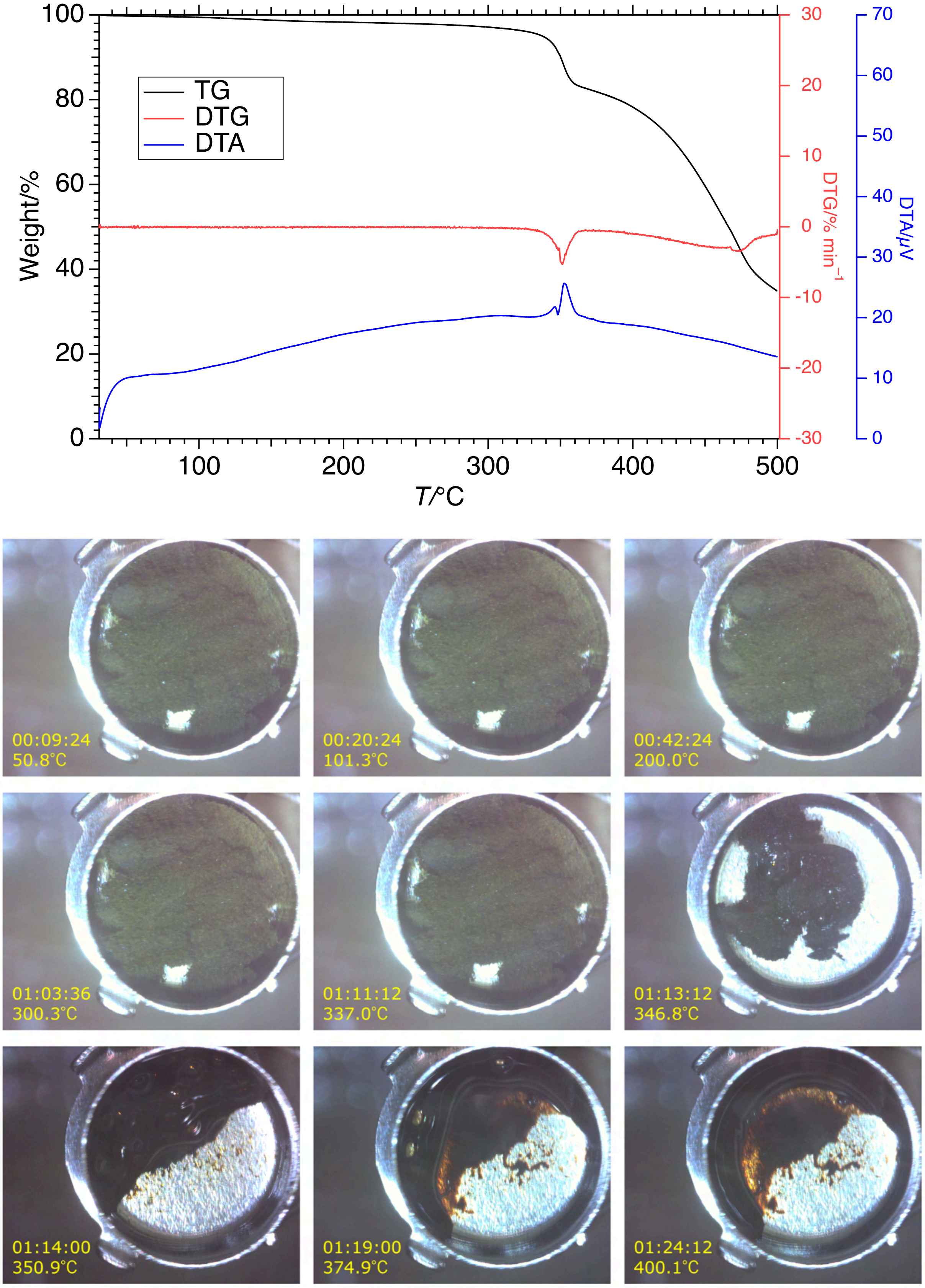

Figure S5. TG-DTA plot of **1** under and atmosphere of N_2. Scan rate: 5 °C/min.

4. NMR Spectra

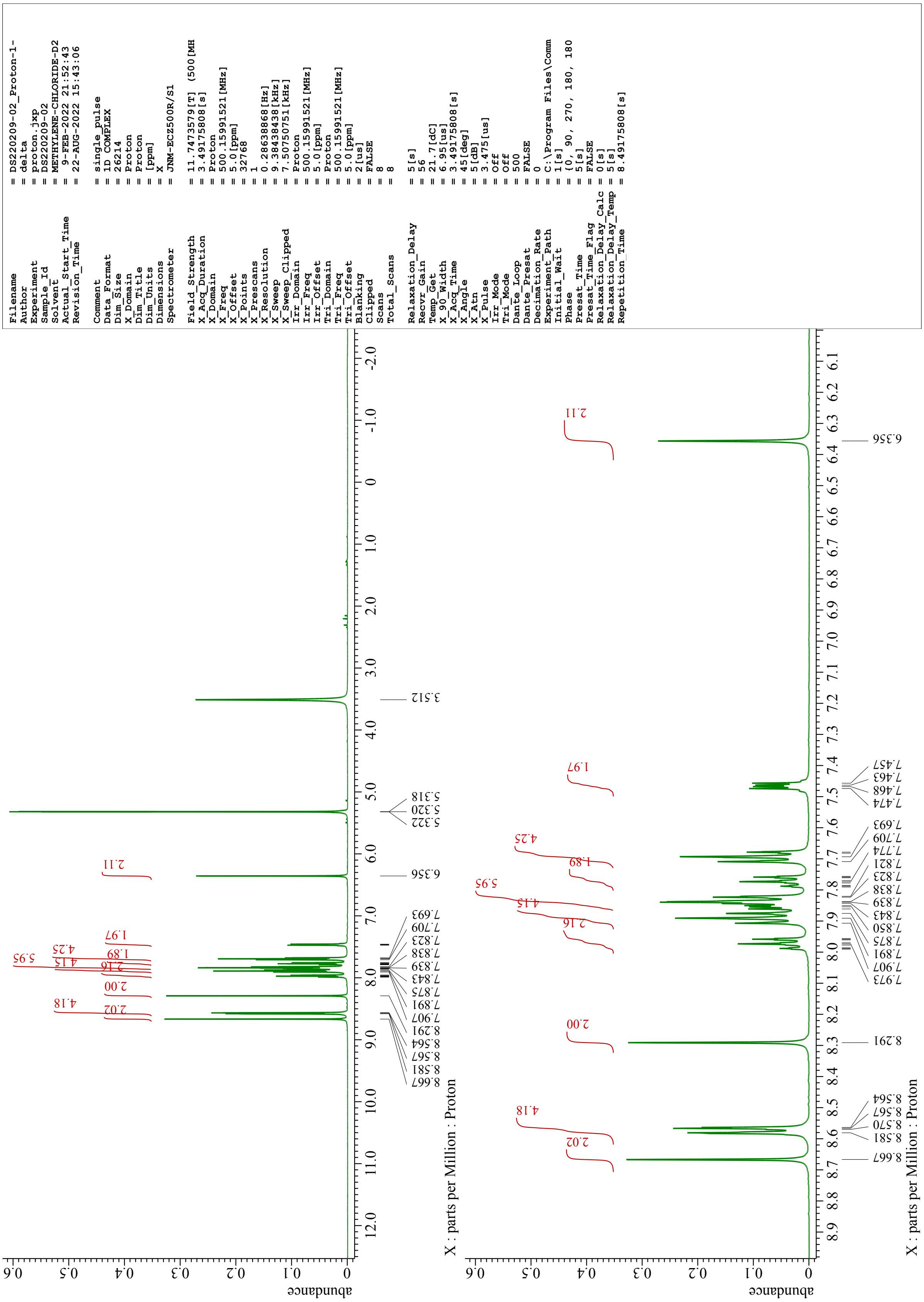

Figure S6. ^{1}H NMR spectrum of **1** in CD_2Cl_2 at room temperature.

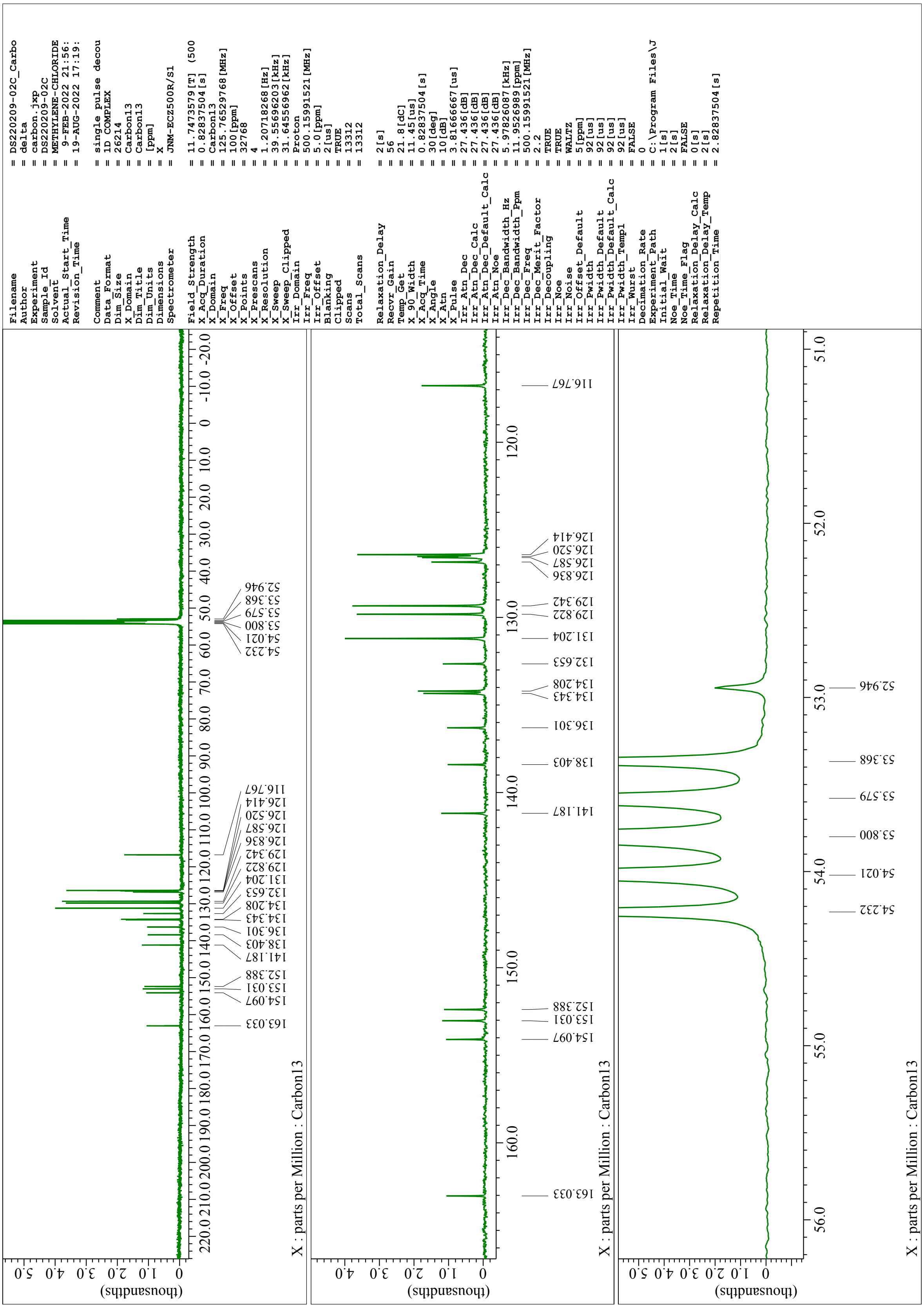

Figure S7. ^{13}C NMR spectrum of **1** in CD_2Cl_2 at room temperature.

5. X-Ray Crystallography Data

Table S1. Crystal data for **1**.

	**1**
**Formula**	$C_{46}H_{30}N_6$, 2 CH_2Cl_2
**FW**	836.61
**Crystal system**	Monoclinic
**Space group**	*C*2
a **/ Å**	20.5022(11)
b **/ Å**	8.0092(5)
c **/ Å**	12.2941(8)
α	90°
β	100.556(6)°
γ	90°
V / Å^3	1984.6(2)
Z	2
T **/ K**	143
$\rho_{calcd.}$ / g cm^{-3}	1.400
R_1 [I>2σ (I)]	0.0880
R_w (all data)	0.2547
**GOF**	1.026
**CCDC**	2214656

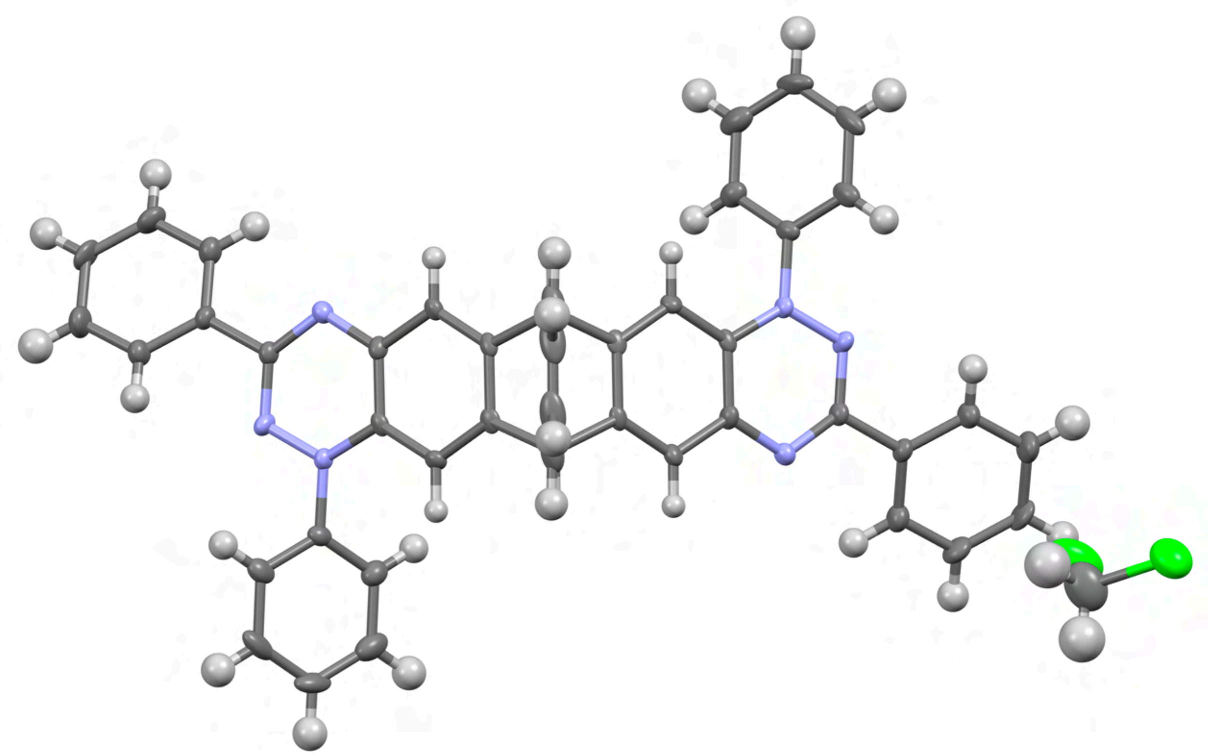

Figure S8. X-Ray crystal structure of **1**. Thermal ellipsoids are scaled at the 50% probability level.

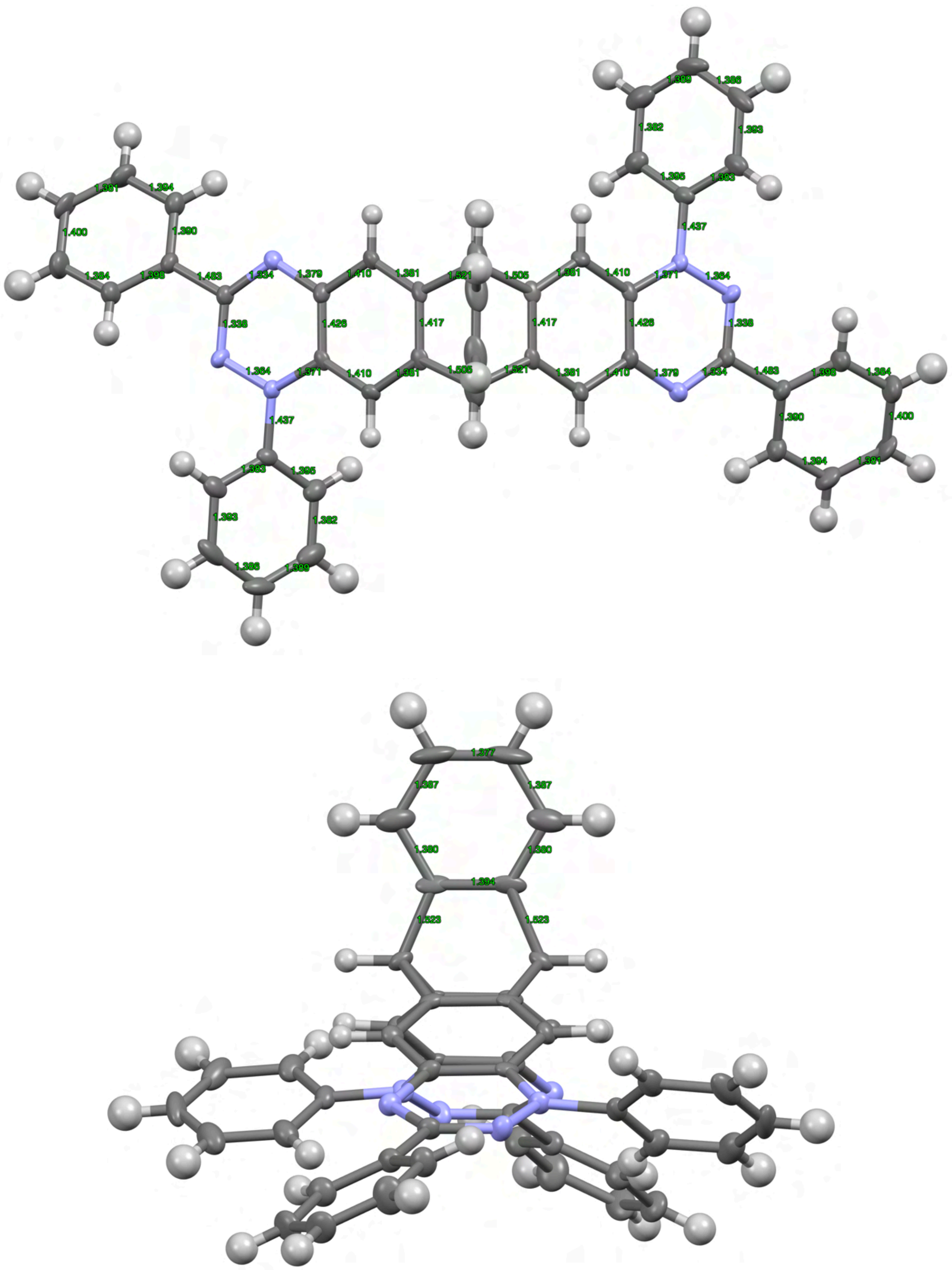

Figure S9. Bond lengths of **1**.

(from the *c*-axis)

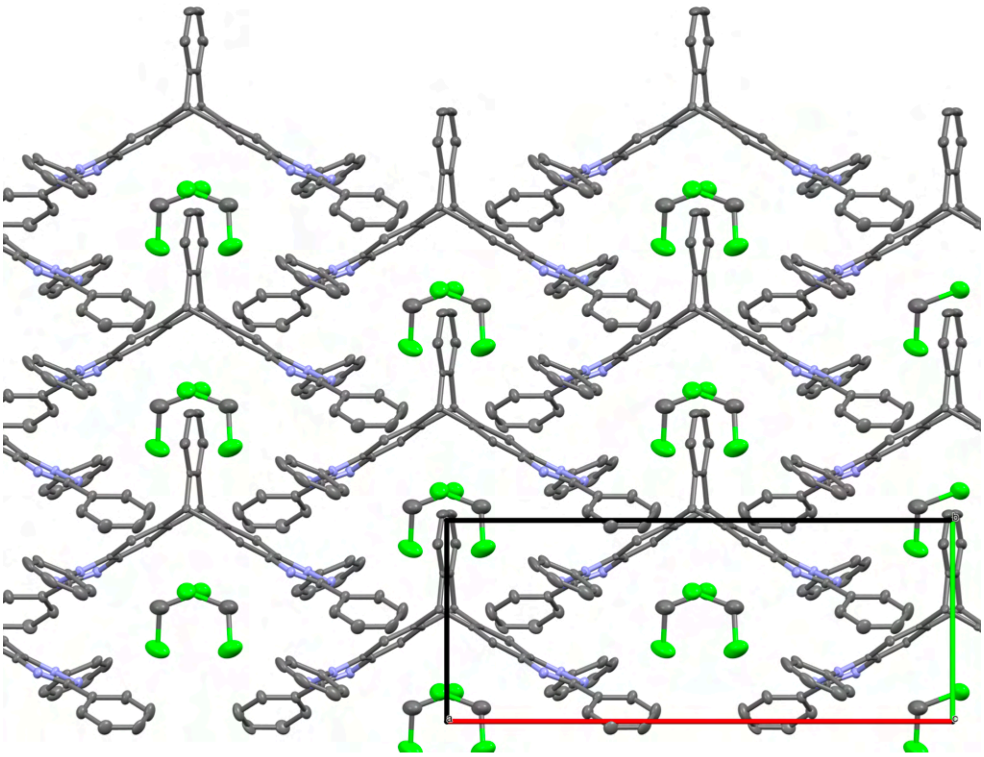

(from the *b*-axis)

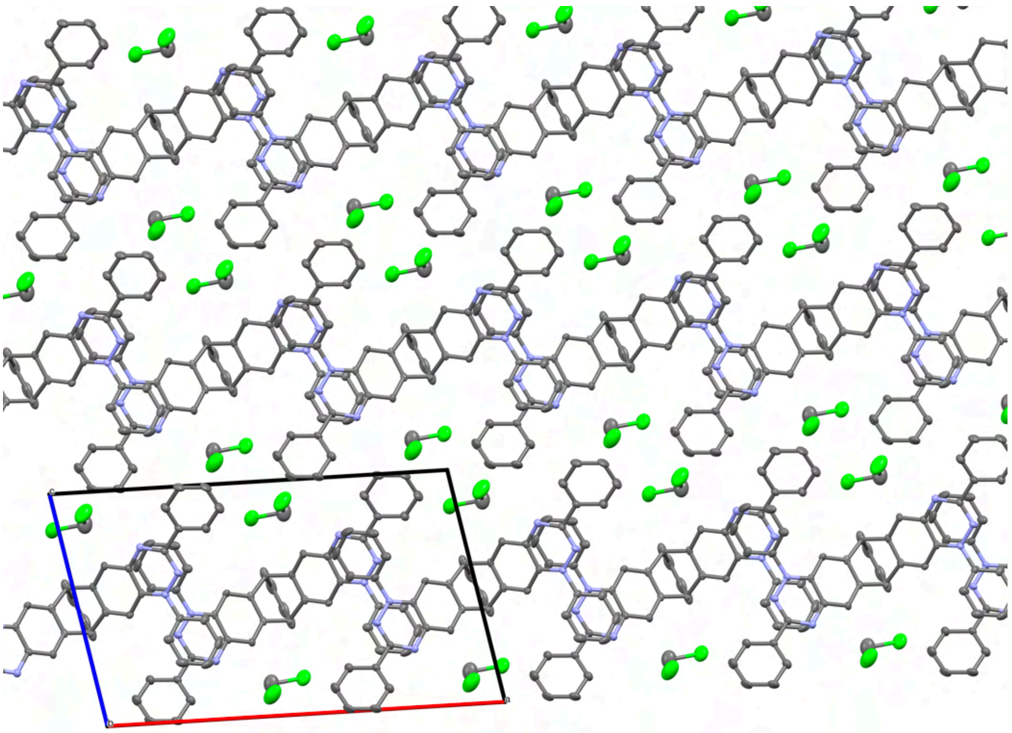

Figure S10. Packing structure of **1**.

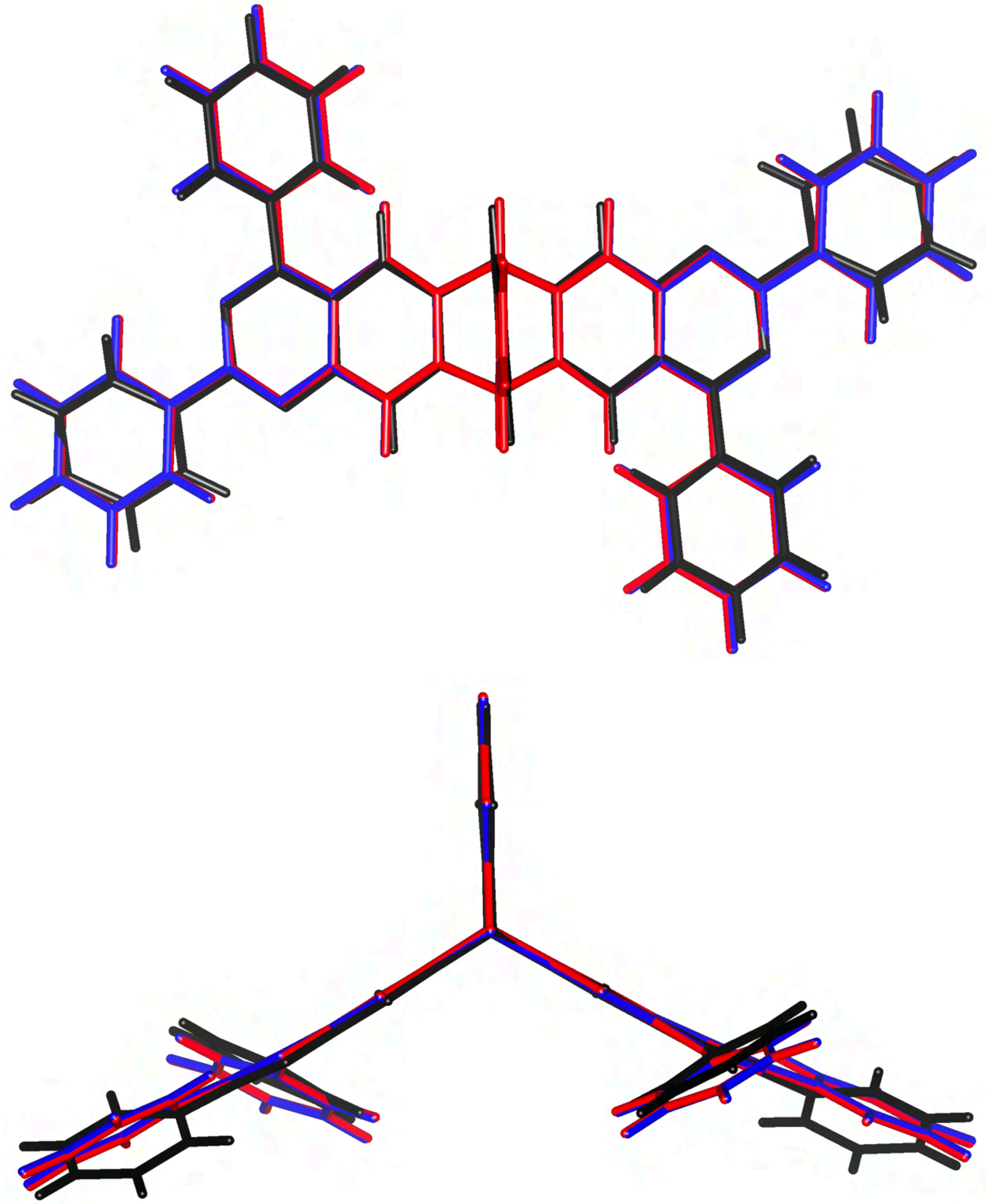

Figure S11. Overlay of X-ray (black) and DFT-optimized (singlet: blue, triplet: red) structures of **1**. The structures were consistent except for the dihedral angles around phenyl groups. The drawing was generated with the VESTA program.[S3,S4]

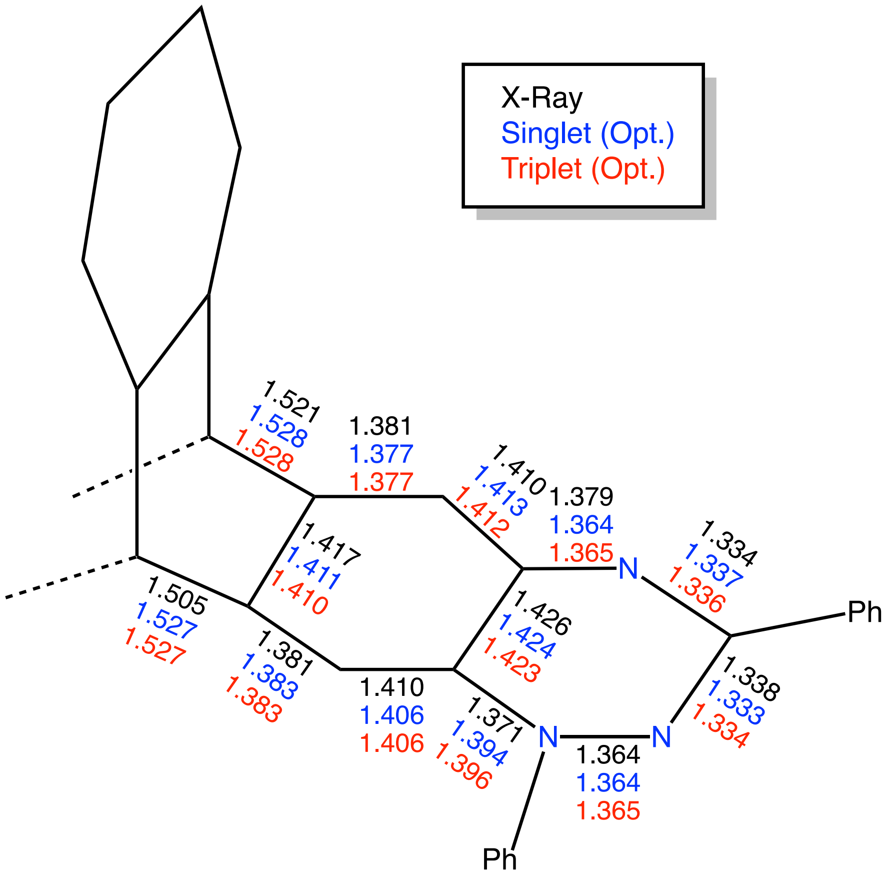

Figure S12. Comparison of bond lengths of X-ray (black) and DFT-optimized (singlet: blue, triplet: red) structures of **1**.

6. Magnetometry Data

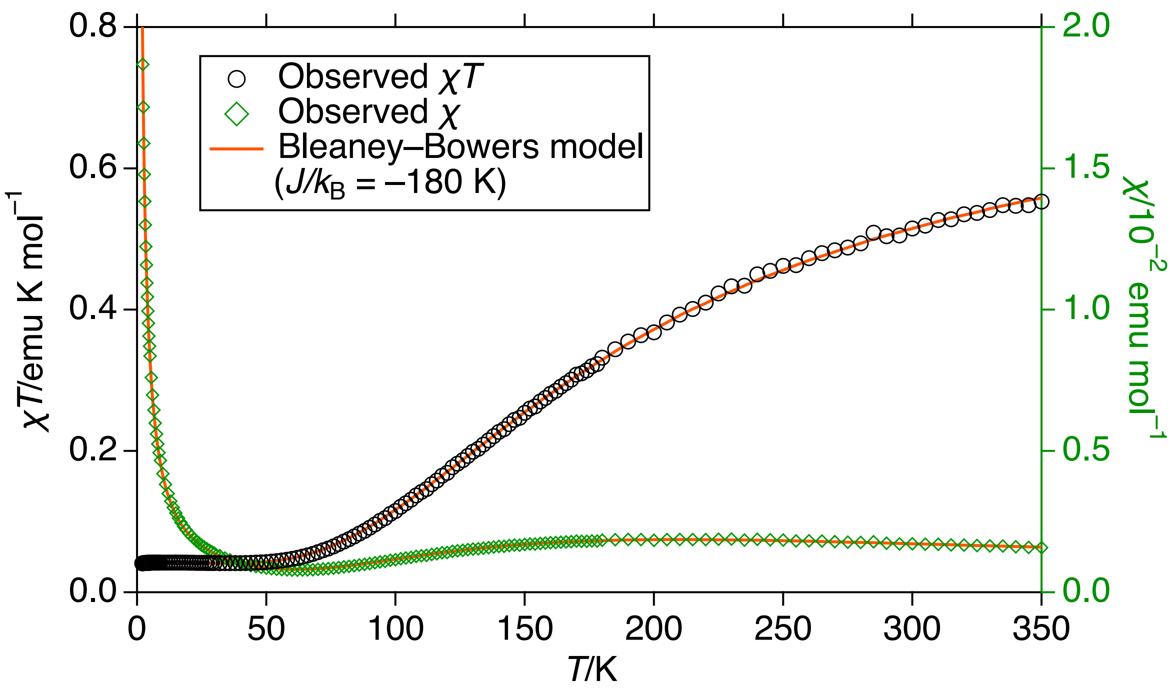

Figure S13. Observed (circles) and fitted (line) χ–T and χT–T curves of **1** observed under 0.5 T. The simulation curved was obtained by least square fitting of the observed data using the equation below.

$$\chi T = \frac{N_A \mu_B^2 g^2}{k_B[3 + \exp(-\frac{2J}{k_B T})]} + f\frac{N_A \mu_B^2 g^2}{4k_B}$$

where N_A = Avogadro constant, μ_B = Bohr magneton, g = g-factor (fixed to 2.0), k_B = Boltzmann constant, J = exchange interaction, f = coefficients for paramagnetic impurity.

The fitted parameters were J/k_B = –180 K and f = 0.05.

7. EPR Spectra

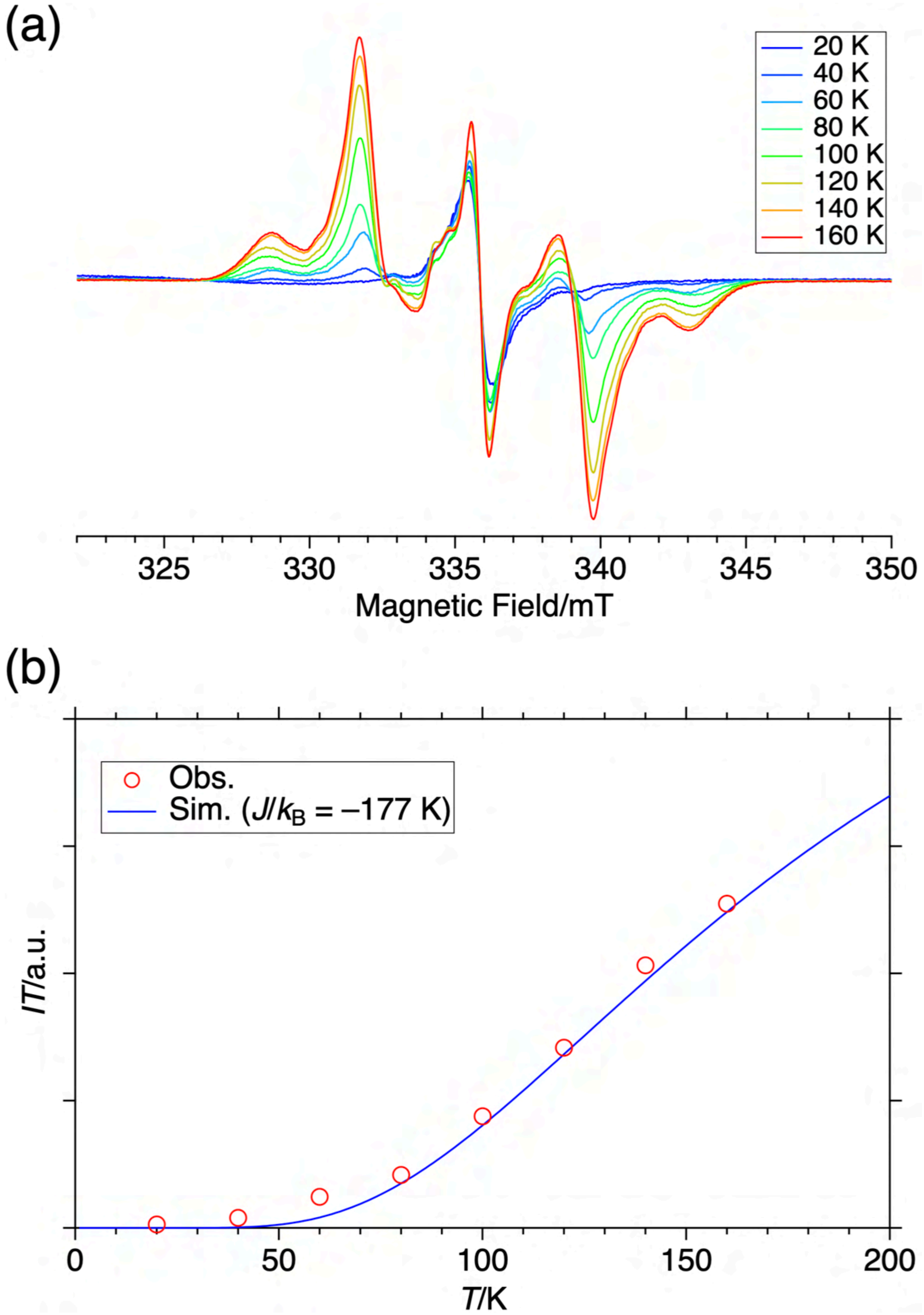

Figure S14. (a) VT-EPR spectra from 20 to 160 K in toluene and (b) derived IT–T curve of **1**.

8. Optical Spectra

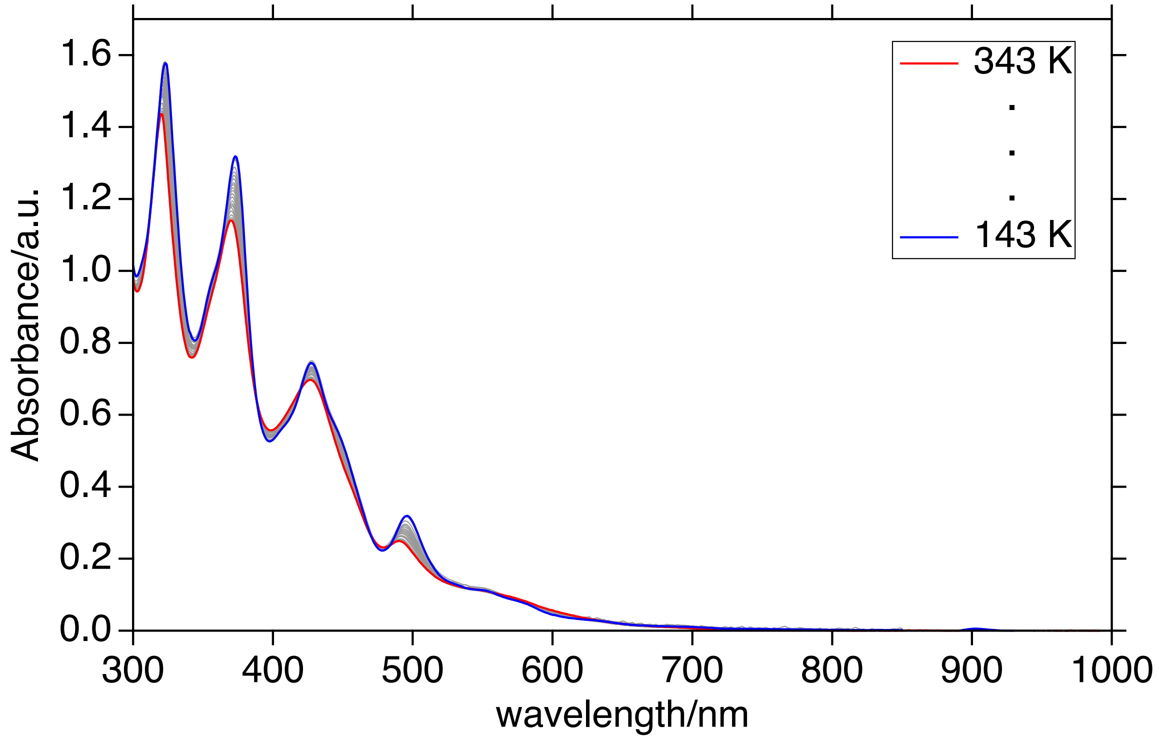

Figure S15. Temperature-dependent absorption spectra of monomer **6** in 2-MeTHF. At temperatures below 143 K, we could not measure the spectra due to turbidity.

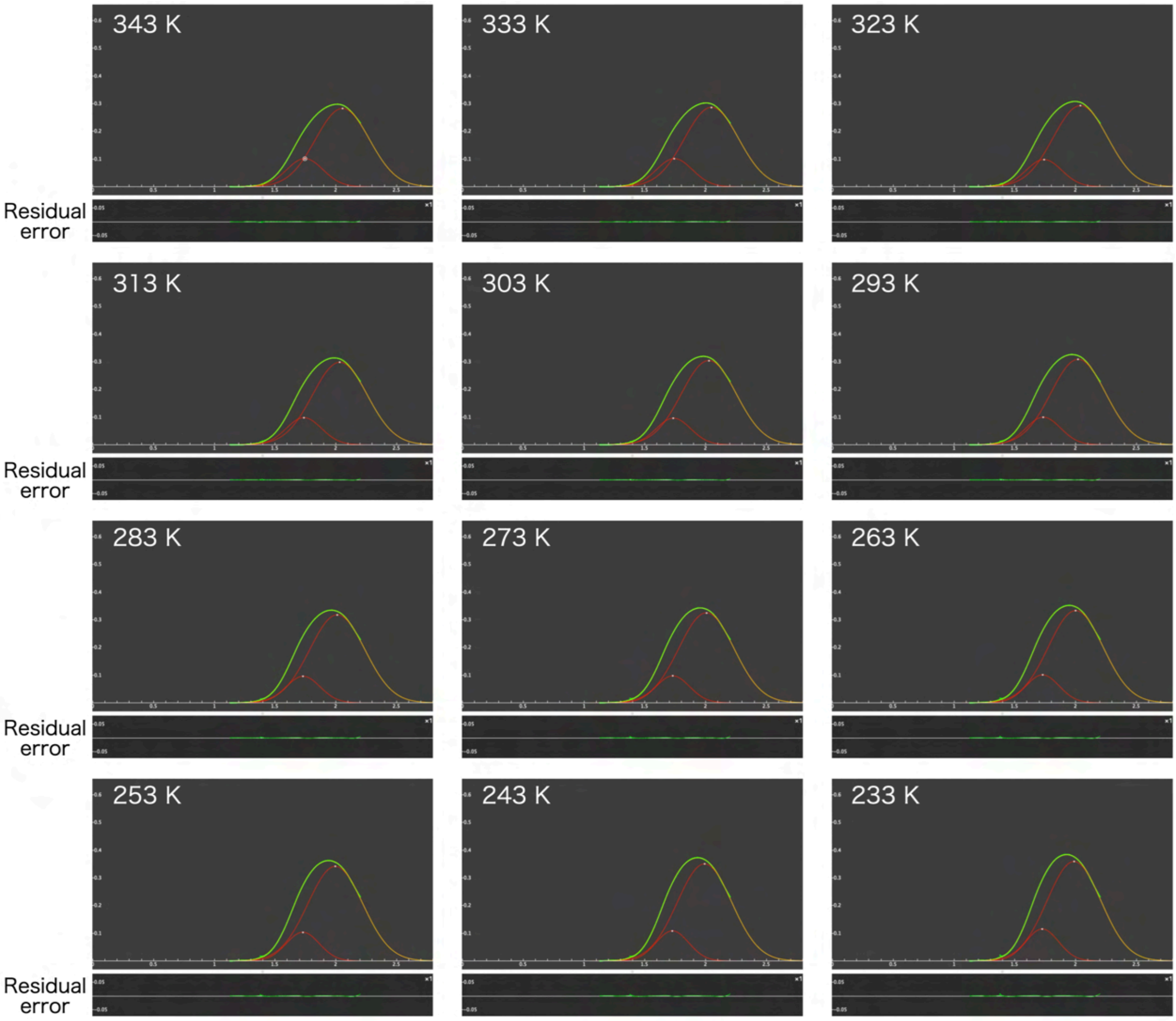

Figure S16. Two-component Gaussian fitting of the absorption spectra of **1** in 2-MeTHF below 2.2 eV (343–233 K). Green: Experimental spectra; Red: Deconvoluted Gaussian curves; Yellow: Simulated spectra.

Note: The NIR band was perfectly reproduced with 2 Gaussian curves. The two components might be assigned as the two transitions split by configuration interaction, which is also predicted by TD-DFT calculations, or vibration structure.

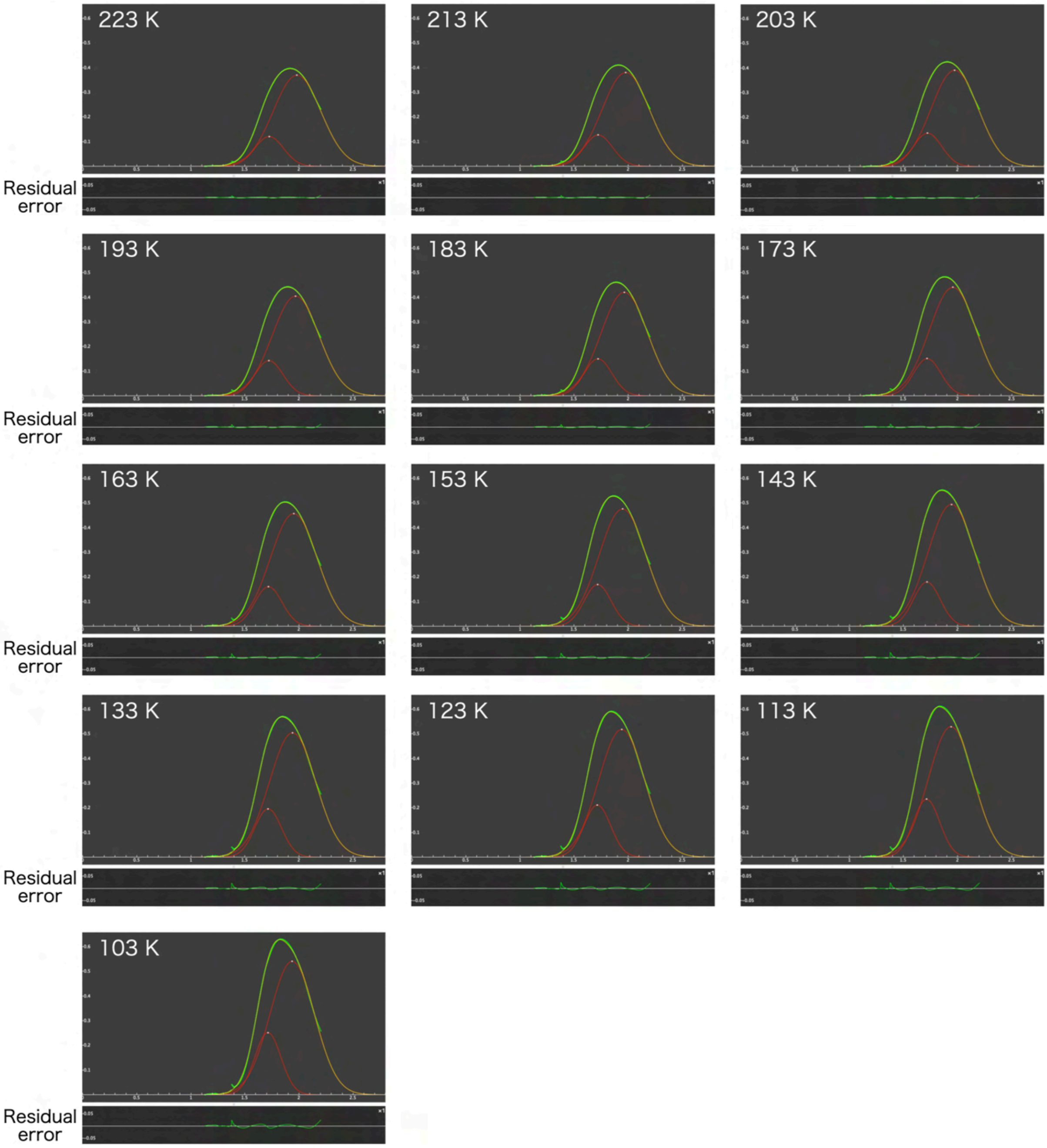

Figure S17. Two-component Gaussian fitting of the absorption spectra of **1** in 2-MeTHF below 2.2 eV (223–103 K). Green: Experimental spectra; Red: Deconvoluted Gaussian curves; Yellow: Simulated spectra.

Table S2. Obtained parameters

	Component 1		Component 2	
Temperature	Center/eV	Area/a.u.	Center/eV	Area/a.u.
343 K	2.055	0.156842	1.745	0.0379688
333 K	2.048	0.159724	1.743	0.0376019
323 K	2.040	0.164302	1.740	0.0358670
313 K	2.033	0.167629	1.737	0.0355065
303 K	2.026	0.171845	1.735	0.0341282
293 K	2.022	0.173770	1.735	0.0354982
283 K	2.012	0.180554	1.731	0.0334594
273 K	2.006	0.184446	1.730	0.0336164
263 K	2.001	0.187769	1.728	0.0345160
253 K	1.996	0.192843	1.728	0.0352869
243 K	1.991	0.195919	1.727	0.0365955
233 K	1.987	0.198800	1.726	0.0388143
223 K	1.982	0.203246	1.725	0.0401996
213 K	1.977	0.208012	1.724	0.0419777
203 K	1.973	0.211899	1.723	0.0445091
193 K	1.969	0.219196	1.722	0.0466282
183 K	1.964	0.226542	1.721	0.0485978
173 K	1.956	0.239448	1.719	0.0479111
163 K	1.952	0.247198	1.718	0.0505002
153 K	1.947	0.258071	1.717	0.0530396
143 K	1.942	0.267501	1.716	0.0556484
133 K	1.941	0.269505	1.715	0.0605250
123 K	1.939	0.274377	1.714	0.0651487
113 K	1.939	0.274895	1.713	0.0716915
103 K	1.937	0.277669	1.712	0.0761653

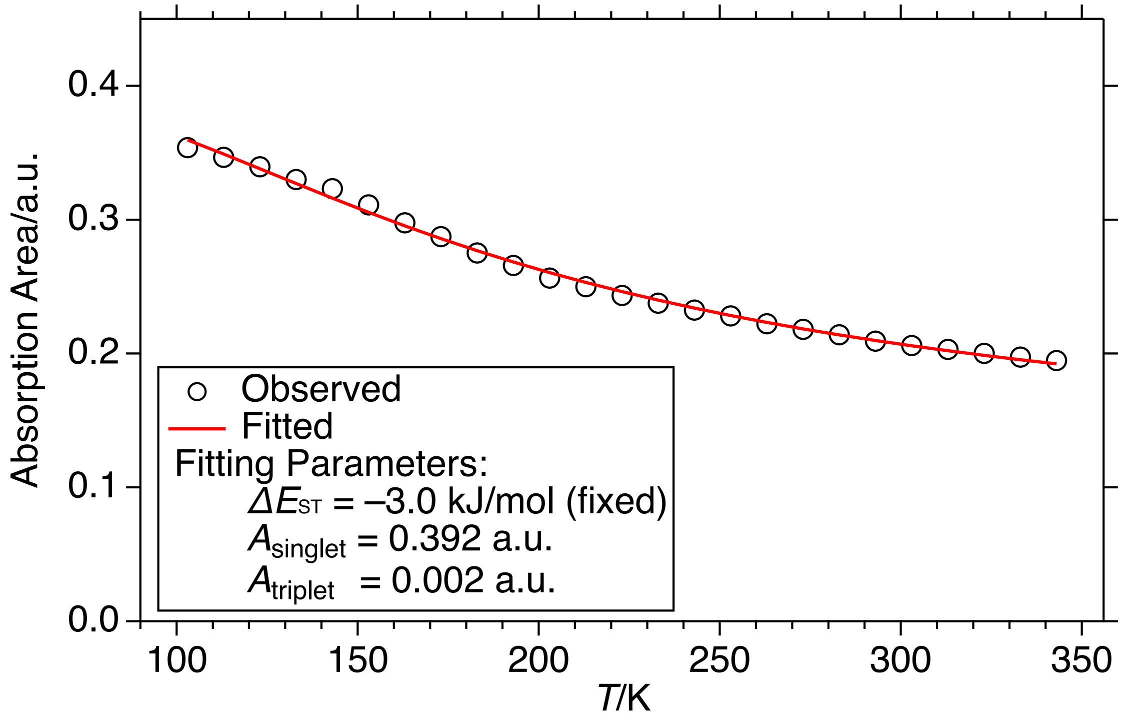

Figure S18. Temperature dependence of absorption area and simulated curve. Simulation was done according to the discussion below.

Net absorption area can be expressed as the following equation.

$$[\text{Net Absorption area}] = [\text{Abundance}]_{\text{singlet}} \times [\text{Area}]_{\text{singlet}} + [\text{Abundance}]_{\text{triplet}} \times [\text{Area}]_{\text{triplet}}$$

The abundance of singlet and triplet is derived from the Boltzmann distribution.

$$\frac{3[\text{Abundance}]_{\text{singlet}}}{[\text{Abundance}]_{\text{triplet}}} = \exp\left(-\frac{\Delta E_{\text{ST}}}{k_{\text{B}}T}\right)$$

$$[\text{Abundance}]_{\text{singlet}} = \frac{\exp\left(-\frac{\Delta E_{\text{ST}}}{k_{\text{B}}T}\right)}{3 + \exp\left(-\frac{\Delta E_{\text{ST}}}{k_{\text{B}}T}\right)}, \qquad [\text{Abundance}]_{\text{triplet}} = \frac{3}{3 + \exp\left(-\frac{\Delta E_{\text{ST}}}{k_{\text{B}}T}\right)}$$

Using the experimental $\Delta E_{\text{ST}}/k_{\text{B}}$ of –360 K (ΔE_{ST} = –3.0 kJ/mol), the net absorption area is estimated as

$$[\text{Net Absorption Area}] = \frac{\exp\left(\frac{360}{T}\right)}{3 + \exp\left(\frac{360}{T}\right)} \times [\text{Area}]_{\text{singlet}} + \frac{3}{3 + \exp\left(\frac{360}{T}\right)} \times [\text{Area}]_{\text{triplet}}$$

Least-squares fitting of the experimentally obtained absorption area with $[\text{Area}]_{\text{singlet}}$ and $[\text{Area}]_{\text{triplet}}$ as variables afforded $[\text{Area}]_{\text{singlet}}$ = 0.392 a.u. and $[\text{Area}]_{\text{triplet}}$ = 0.002 a.u (Figure S18).

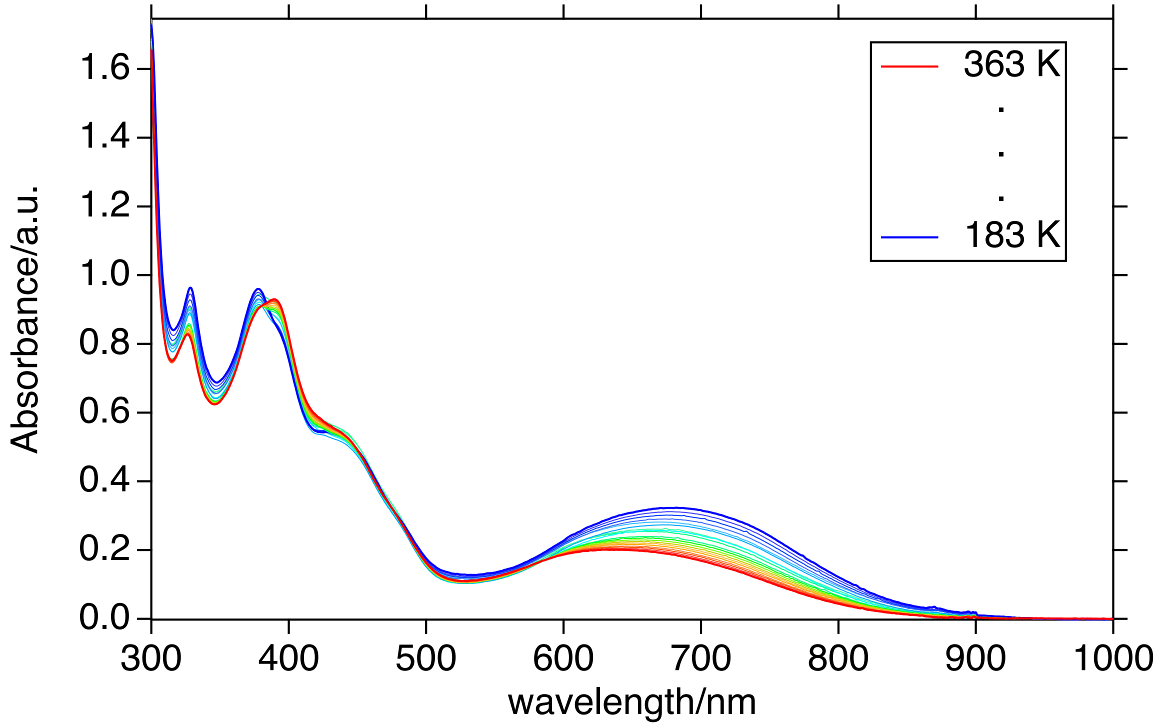

Figure S19. Temperature dependence of the absorption spectra of **1** in toluene.

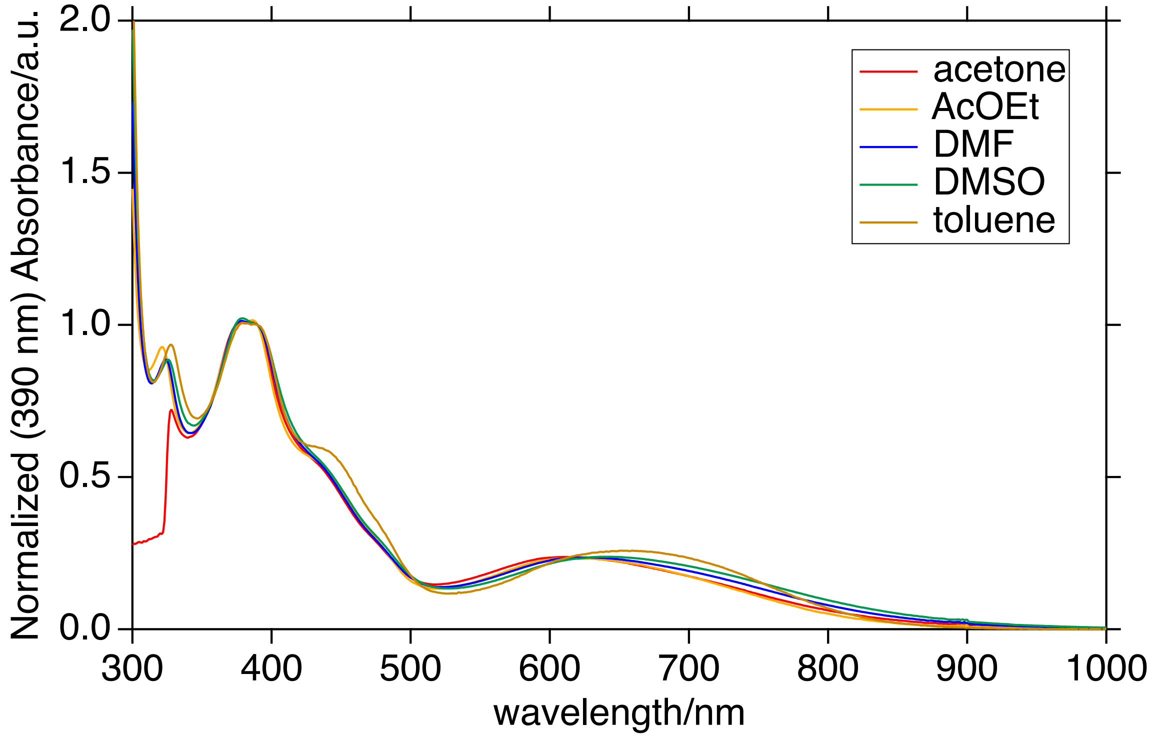

Figure S20. Solvent dependence of the absorption spectra of **1** at 20 °C. **Note**: It seems that the NIR band shows negative solvatochromism, which is indicative of the ground-state broken symmetry polar state with a partial charge separation. However, as shown in Table S3, Gaussian deconvolution indicates that the absorption bands slightly shift from blue to red upon increasing solvent polarity. We could not find clear evidence for the ground-state broken symmetry character of the diradical at this point.

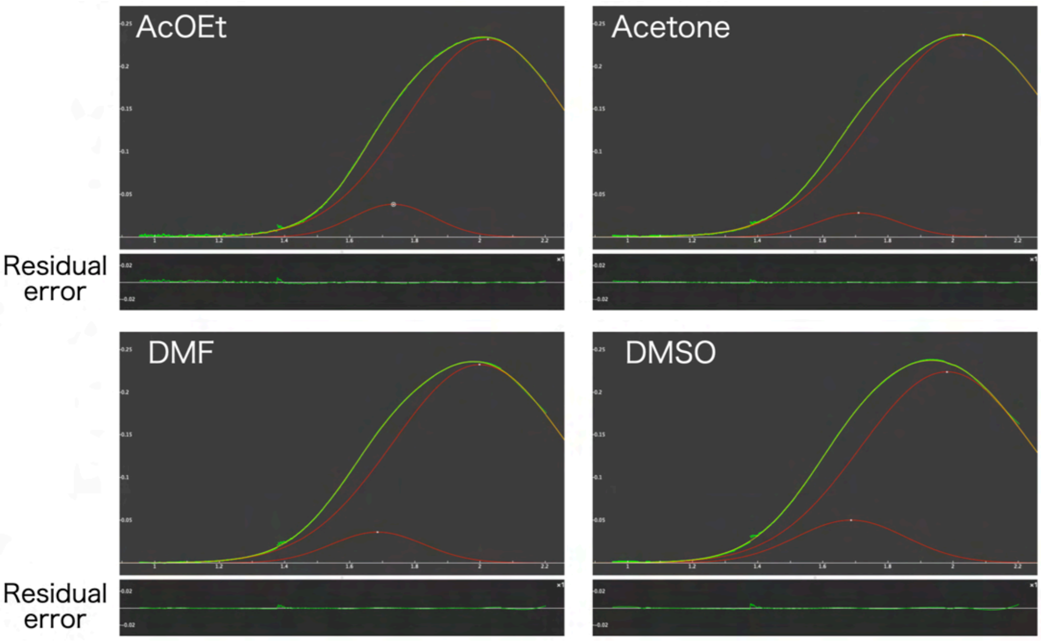

Figure S21. Two-component Gaussian fitting of the absorption spectra of **1** in various solvents below 2.2 eV at 20 °C. Green: Experimental spectra; Red: Deconvoluted Gaussian curves; Yellow: Simulated spectra.

Table S3. Obtained fitting parameters.

Solvent	Dielectric Constant	**Component 1**		**Component 2**	
		Center/eV	Area/a.u.	Center/eV	Area/a.u.
toluene	2.4	2.022	0.173770	1.735	0.0354982
AcOEt	6.0	2.024	0.144554	1.733	0.0120404
Acetone	20.7	2.032	0.161118	1.710	0.0090289
DMF	36.7	2.000	0.155251	1.685	0.0128240
DMSO	46.7	1.981	0.148805	1.686	0.0211162

Table S4. TD-DFT (UB3LYP/6-311G*) calculation results for **1** in the singlet and triplet states, including solvent effect with the IEFPCM model. The calculations were conducted based on the energy-minimized geometries calculated at the same levels (see Tables S19–30 for detailed results).

	Dielectric	**1** (singlet)		**1** (triplet)	
Solvent	Constant	$S_0 \rightarrow S_1$	$S_0 \rightarrow S_2$	$T_1 \rightarrow T_2$	$T_1 \rightarrow T_3$
(Vacuum)	1	803 nm	760 nm	619 nm	601 nm
		(*f* = 0.0002)	(*f* = 0.1123)	(*f* = 0.0002)	(*f* = 0.0027)
toluene	2.4	802 nm	766 nm	627 nm	609 nm
		(*f* = 0.0003)	(*f* = 0.1397)	(*f* = 0.0002)	(*f* = 0.0027)
Acetone	20.7	801 nm	763 nm	634 nm	616 nm
		(*f* = 0.0003)	(*f* = 0.1355)	(*f* = 0.0002)	(*f* =0.0025)
DMSO	46.7	800 nm	763 nm	635 nm	617 nm
		(*f* = 0.0003)	(*f* = 0.1378)	(*f* = 0.0002)	(*f* = 0.0025)

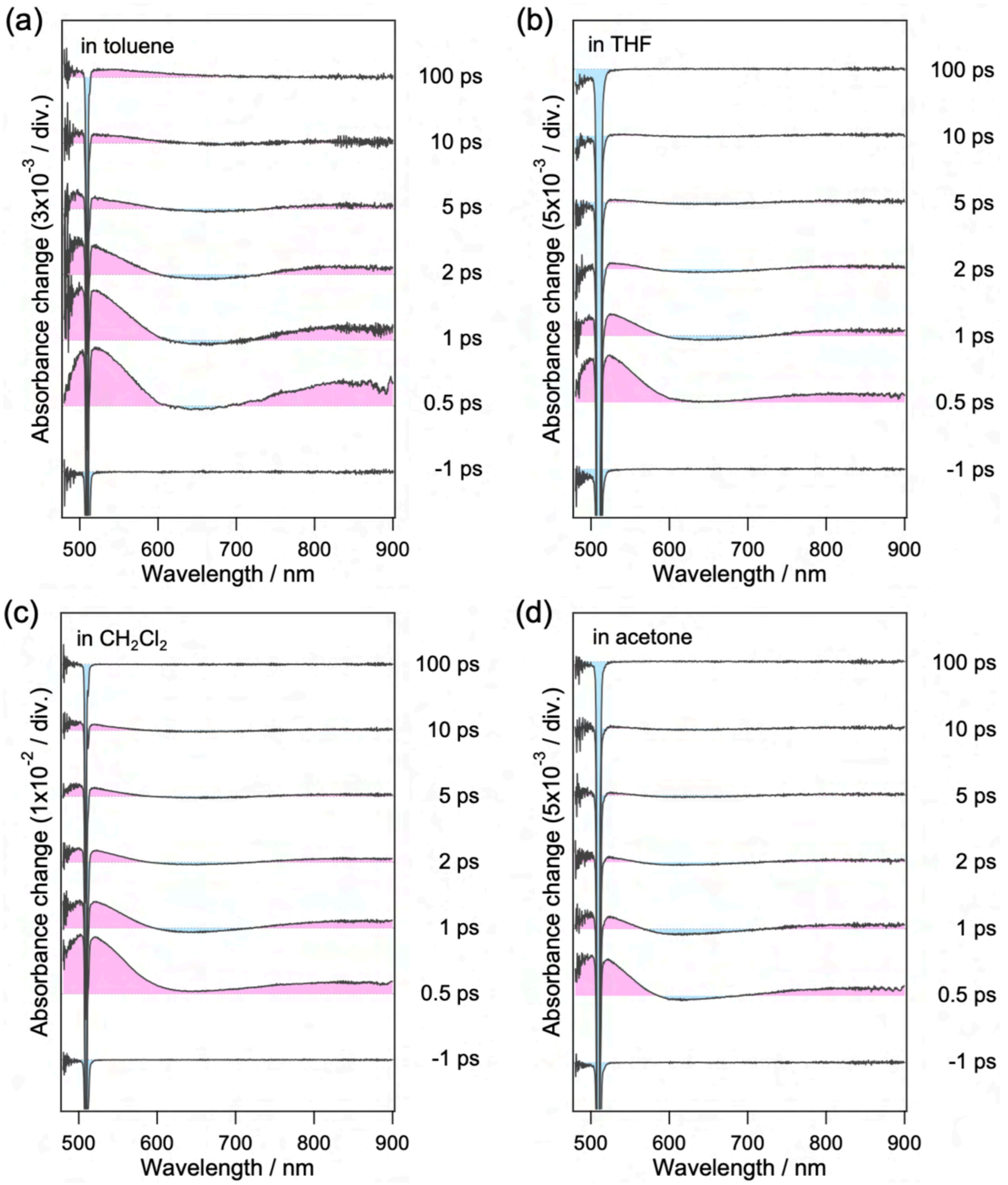

Figure S22. Transient absorption spectra of **1** in (a) toluene, (b) THF, (c) CH_2Cl_2, and (d) acetone excited with a femtosecond laser pulse at 514 nm. A positive band remaining at 100 ps in spectrum (a) is due to the excimer of toluene produced by simultaneous two-photon absorption.

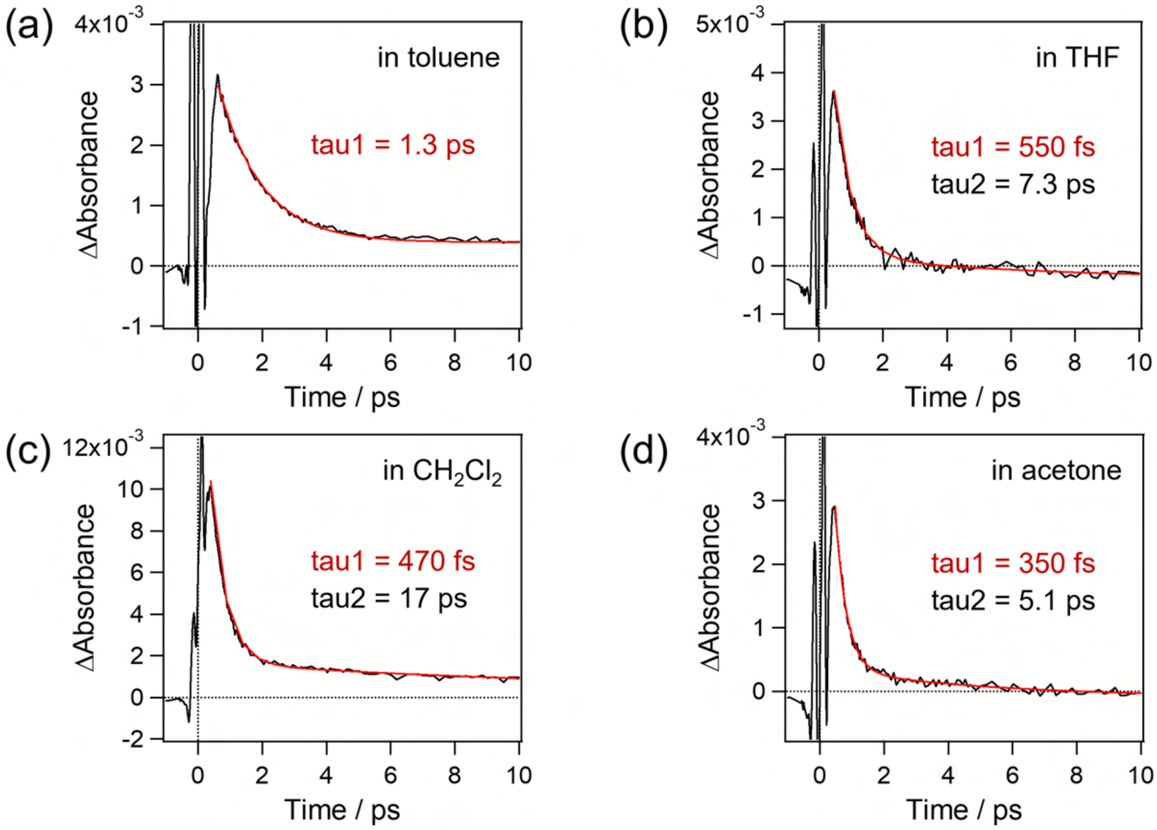

Figure S23. Time profiles of transient absorbance of **1** in (a) toluene, (b) THF, (c) CH_2Cl_2 and (d) acetone. The monitoring wavelength was set to 520 nm. The first time constant corresponds to the lifetime of the S_1 state with the symmetry-breaking charge transfer nature, and the second time constant in (b-d) is due to the vibrational cooling in the ground state.

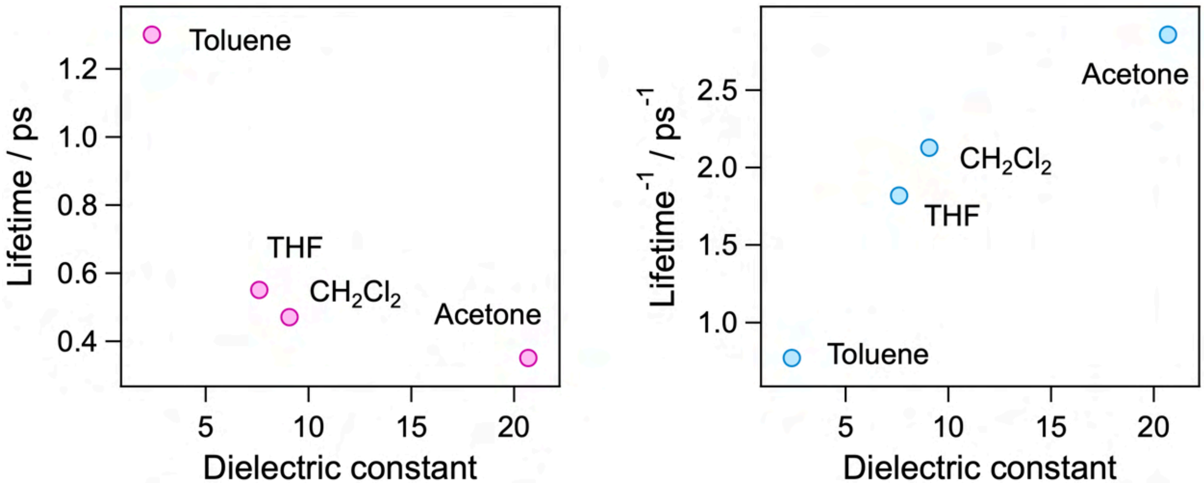

Figure S24. Relationship between the excited state lifetime of **1** and dielectric constants of the solvent.

9. Electrochemistry

According to the Rehm–Weller equation,

$$\Delta G^0 = \Delta E_{\mathrm{redox}} - E_{00} - \frac{e}{4\pi\varepsilon_0\varepsilon_s R_{\mathrm{cc}}} - \frac{e}{8\pi\varepsilon_0}\left(\frac{1}{r^+} + \frac{1}{r^-}\right)\left(\frac{1}{\varepsilon_{ref}} - \frac{1}{\varepsilon_s}\right)$$

We adopted $\Delta E_{\mathrm{redox}}$ = 1.23 eV from electrochemistry, E_{00} = 1.74 eV from absorption spectrum in 2-MeTHF, $r^+ = r^-$ = 2.5 Å from the diameter of benzotriazinyl unit.

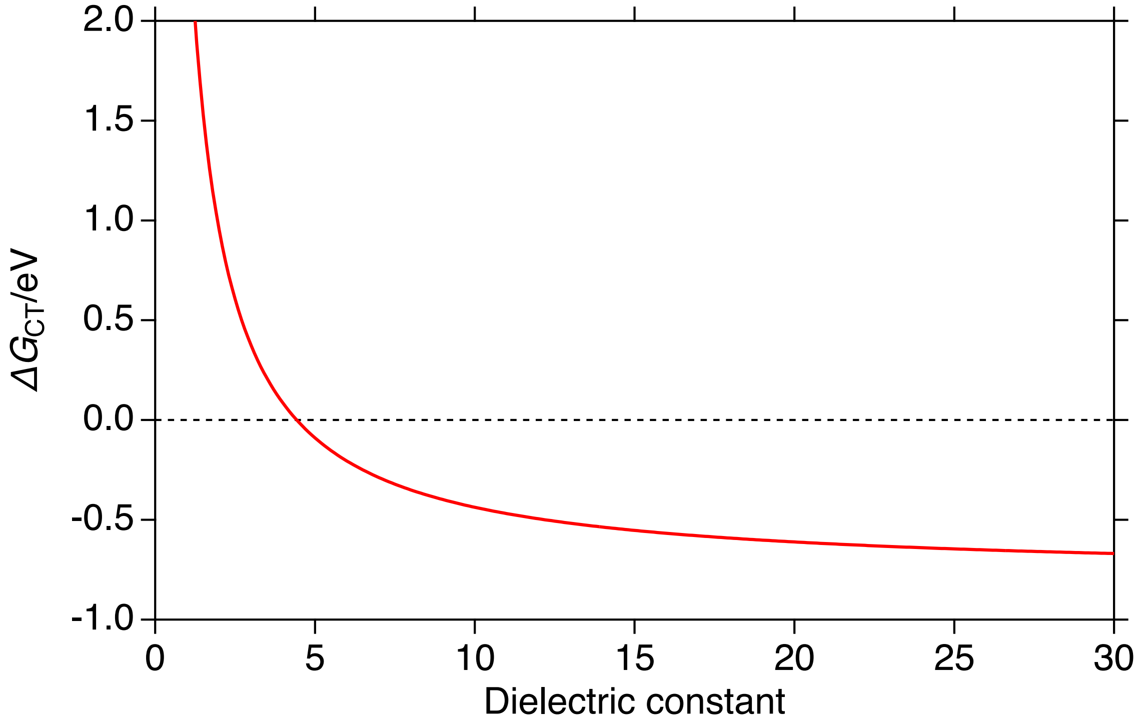

Figure S25. Estimated relationship between the CT state energy of **1** and dielectric constant of media.

10. Quantum Chemical Calculation Results

Table S5. Result of RAS(2,2)-SF/def2-TZVP calculation of **1** based on the X-ray geometry.

```
**************************************************
 RAS-CI total energy for state   1:  -2086.727360963383
  Excitation energy (eV) =  0.000000000000
  Multiplicity: Singlet
  Dipole Moment:    0.0012 X    -0.0003 Y    -0.5358 Z
  Amplitudes :

 | HOLE  | ALPHA | BETA  | PART  |    AMPLITUDE
--------------------------------------------------
 |       | 10    | 10    |       |    7.4674058181391E-01
 |       | 01    | 01    |       |   -6.6475922602283E-01
--------------------------------------------------
*** Contributions RASCI wfn    Active:  99.95
                            Hole:   0.02
                            Part:   0.03

**************************************************
 RAS-CI total energy for state   2:  -2086.726758056166
  Excitation energy (eV) =  0.016405940097
  Multiplicity: Triplet
  Dipole Moment:    0.0012 X    -0.0003 Y    -0.5275 Z
  Trans. Moment:    0.0000 X     0.0000 Y     0.0000 Z
  Strength   :  0.000000
  Amplitudes :

 | HOLE  | ALPHA | BETA  | PART  |    AMPLITUDE
--------------------------------------------------
 |       | 10    | 01    |       |    7.0699652634101E-01
 |       | 01    | 10    |       |   -7.0699652307177E-01
--------------------------------------------------
*** Contributions RASCI wfn    Active:  99.97
                            Hole:   0.02
                            Part:   0.01
```

Table S6. Result of CASSCF(2,2)/6-311G* calculation of **1** based on the X-ray geometry.

```
(1) EIGENVALUE -2086.395945
(1) 0.7456463 (3)-0.6663420 (2)-0.0000025 (
 Final one electron symbolic density matrix:
              1           2
     1  0.111198D+01
     2 -0.570620D-06   0.888023D+00
```

Table S7. Optimized molecular geometry of the lowest doublet state of **6** (UB3LYP/6-311G*).

Center Number	Atomic Number	Atomic Type	Coordinates (Angstroms) X	Y	Z
1	6	0	-1.325314	0.322049	0.048344
2	6	0	0.065908	2.147810	0.035261
3	6	0	1.200781	1.295732	-0.069792
4	1	0	-0.606656	4.171774	0.106835
5	6	0	0.269666	3.540558	0.014378
6	6	0	2.478131	1.840214	-0.257886
7	6	0	2.640422	3.219268	-0.286877
8	6	0	1.538690	4.071124	-0.137687
9	1	0	3.334207	1.191960	-0.389822
10	1	0	1.679027	5.146677	-0.154987
11	6	0	1.954655	-1.078048	0.009852
12	6	0	2.991278	-1.019532	0.944022
13	6	0	1.874588	-2.146661	-0.885321
14	6	0	3.958041	-2.020997	0.963622
15	1	0	3.026882	-0.208877	1.662323
16	6	0	2.843441	-3.143999	-0.854861
17	1	0	1.050937	-2.187128	-1.586984
18	6	0	3.890274	-3.083022	0.064028
19	1	0	4.757895	-1.976218	1.695339
20	1	0	2.780654	-3.971081	-1.554282
21	6	0	-2.699002	-0.250911	0.053801
22	6	0	-2.909156	-1.636542	0.062100
23	6	0	-3.807760	0.604629	0.051316
24	6	0	-4.200867	-2.151867	0.064693
25	1	0	-2.053636	-2.300077	0.072044
26	6	0	-5.098305	0.085128	0.053818
27	1	0	-3.638520	1.673882	0.048553
28	6	0	-5.300144	-1.293951	0.059761
29	1	0	-4.350543	-3.226988	0.073126
30	1	0	-5.949110	0.759230	0.050961
31	1	0	-6.307595	-1.698325	0.062293
32	7	0	-1.203160	1.648682	0.126044
33	7	0	-0.332134	-0.567576	-0.023140
34	7	0	0.936455	-0.074111	-0.007690
35	1	0	3.632459	3.633390	-0.431865
36	1	0	4.643914	-3.863074	0.084531

Table S8. Optimized molecular geometry of the first excited doublet state of **6** (UB3LYP/6-311G*).

Center Number	Atomic Number	Atomic Type	Coordinates (Angstroms) X	Y	Z
1	6	0	-1.376489	0.324907	0.054304
2	6	0	0.032583	2.144434	0.023147
3	6	0	1.194484	1.257028	-0.089475
4	1	0	-0.577923	4.210287	0.140917
5	6	0	0.282179	3.559655	0.032764
6	6	0	2.498719	1.792849	-0.274437
7	6	0	2.665842	3.148595	-0.288820
8	6	0	1.544516	4.042064	-0.114901
9	1	0	3.344752	1.132624	-0.415154
10	1	0	1.722259	5.112070	-0.119105
11	6	0	1.956131	-1.069389	0.015016
12	6	0	2.878082	-1.081981	1.056749
13	6	0	1.984238	-2.025937	-0.994268
14	6	0	3.857488	-2.072054	1.077901
15	1	0	2.817680	-0.341251	1.845507
16	6	0	2.973408	-3.004557	-0.967694
17	1	0	1.236861	-2.000020	-1.777785
18	6	0	3.909341	-3.028268	0.065387
19	1	0	4.574122	-2.098636	1.891437
20	1	0	3.008905	-3.751413	-1.753172
21	6	0	-2.697237	-0.236687	0.053709
22	6	0	-2.910157	-1.640038	0.044311
23	6	0	-3.842015	0.602333	0.057373
24	6	0	-4.195308	-2.161765	0.035266
25	1	0	-2.056540	-2.306011	0.050237
26	6	0	-5.116189	0.064167	0.049477
27	1	0	-3.701210	1.676104	0.066481
28	6	0	-5.312151	-1.323260	0.037166
29	1	0	-4.329763	-3.239904	0.029038
30	1	0	-5.974309	0.730366	0.052636
31	1	0	-6.314439	-1.738543	0.030179
32	7	0	-1.185960	1.663240	0.090632
33	7	0	-0.295479	-0.582444	0.068966
34	7	0	0.903535	-0.067432	-0.005935
35	1	0	3.655440	3.564872	-0.439994
36	1	0	4.674703	-3.796672	0.085292

Table S9. Optimized molecular geometry of the lowest singlet state of **1** (UB3LYP/6-311G*).

Center Number	Atomic Number	Atomic Type	Coordinates (Angstroms) X	Y	Z
1	6	0	-0.035075	1.298378	2.064521
2	6	0	1.195805	0.739216	1.354021
3	6	0	1.230198	-0.671738	1.349918
4	6	0	0.035096	-1.297236	2.065236
5	6	0	-0.019772	0.702142	3.471181
6	6	0	0.019863	-0.700226	3.471577
7	6	0	-1.195807	-0.738466	1.354471
8	6	0	-1.230203	0.672483	1.349589
9	6	0	-2.263021	1.355192	0.734779
10	6	0	-3.284815	0.625374	0.103020
11	6	0	-3.289044	-0.797807	0.142417
12	6	0	-2.210467	-1.461320	0.767585
13	6	0	2.262992	-1.354787	0.735453
14	6	0	3.284767	-0.625324	0.103251
15	6	0	3.289021	0.797875	0.141899
16	6	0	2.210457	1.461740	0.766721
17	6	0	-0.039307	1.402870	4.668475
18	6	0	-0.019317	0.697389	5.875791
19	6	0	0.019675	-0.694034	5.876175
20	6	0	0.039534	-1.400242	4.669279
21	7	0	-4.360159	1.213601	-0.561479
22	7	0	-5.435945	0.489713	-0.983968
23	6	0	-5.348001	-0.835040	-0.871848
24	7	0	-4.316131	-1.528076	-0.379853
25	7	0	4.360077	-1.213922	-0.560989
26	7	0	5.435968	-0.490284	-0.983647
27	6	0	5.348042	0.834531	-0.872236
28	7	0	4.316123	1.527854	-0.380754
29	6	0	4.470400	-2.618996	-0.801276
30	6	0	3.429835	-3.315697	-1.420120
31	6	0	3.566165	-4.678369	-1.670222
32	6	0	4.736972	-5.345740	-1.315690
33	6	0	5.778497	-4.640735	-0.713826
34	6	0	5.650969	-3.280016	-0.455942
35	6	0	6.526099	1.600158	-1.363785
36	6	0	6.560181	2.992385	-1.216847
37	6	0	7.655368	3.722002	-1.668440
38	6	0	8.730289	3.073088	-2.273294
39	6	0	8.702162	1.687164	-2.425281
40	6	0	7.609394	0.954329	-1.975053
41	6	0	-4.470594	2.618586	-0.802300
42	6	0	-5.651062	3.279710	-0.456846
43	6	0	-5.778706	4.640325	-0.715233
44	6	0	-4.737386	5.345105	-1.317713
45	6	0	-3.566673	4.677623	-1.672350
46	6	0	-3.430238	3.315051	-1.421747
47	6	0	-6.525974	-1.600962	-1.363143
48	6	0	-6.559707	-2.993191	-1.216143
49	6	0	-7.654803	-3.723082	-1.667515
50	6	0	-8.729990	-3.074443	-2.272189
51	6	0	-8.702219	-1.688516	-2.424224
52	6	0	-7.609537	-0.955411	-1.974231
53	1	0	-0.063137	2.388612	2.064835
54	1	0	0.063155	-2.387471	2.066142
55	1	0	-2.288659	2.437323	0.747475
56	1	0	-2.229702	-2.545749	0.777030
57	1	0	2.288629	-2.436910	0.748750
58	1	0	2.229704	2.546174	0.775575
59	1	0	-0.069154	2.488576	4.670148
60	1	0	0.069370	-2.485942	4.671678
61	1	0	2.531615	-2.790132	-1.722586
62	1	0	2.758824	-5.214554	-2.157935
63	1	0	4.840738	-6.406966	-1.515432
64	1	0	6.695602	-5.152278	-0.440940
65	1	0	6.455985	-2.718603	0.001339
66	1	0	5.718715	3.485453	-0.747014
67	1	0	7.669673	4.800662	-1.547375
68	1	0	9.584172	3.643243	-2.625860
69	1	0	9.534137	1.175409	-2.898876
70	1	0	7.581854	-0.121173	-2.096964
71	1	0	-6.455921	2.718464	0.000921
72	1	0	-6.695734	5.151961	-0.442263
73	1	0	-4.841238	6.406249	-1.517848
74	1	0	-2.759498	5.213637	-2.160527
75	1	0	-2.532100	2.789374	-1.724264
76	1	0	-5.718054	-3.486040	-0.746417
77	1	0	-7.668825	-4.801743	-1.546420
78	1	0	-9.583806	-3.644811	-2.624576
79	1	0	-9.534412	-1.176971	-2.897660
80	1	0	-7.582270	0.120093	-2.096197
81	1	0	0.034639	-1.235046	6.816791
82	1	0	-0.034179	1.238913	6.816111

Table S10. Optimized molecular geometry of the lowest triplet state of **1** (UB3LYP/6-311G*).

Center Number	Atomic Number	Atomic Type	Coordinates (Angstroms) X	Y	Z
1	6	0	-0.035666	1.298319	2.028254
2	6	0	1.198534	0.739330	1.322654
3	6	0	1.234110	-0.671175	1.319047
4	6	0	0.035719	-1.296944	2.029122
5	6	0	-0.019822	0.702409	3.435943
6	6	0	0.019987	-0.700107	3.436429
7	6	0	-1.198524	-0.738431	1.323220
8	6	0	-1.234110	0.672067	1.318680
9	6	0	-2.273627	1.353954	0.714857
10	6	0	-3.301968	0.623196	0.094565
11	6	0	-3.304783	-0.798681	0.134256
12	6	0	-2.218991	-1.461814	0.747436
13	6	0	2.273589	-1.353466	0.715625
14	6	0	3.301899	-0.623129	0.094787
15	6	0	3.304746	0.798770	0.133567
16	6	0	2.218987	1.462321	0.746355
17	6	0	-0.039234	1.403171	4.632917
18	6	0	-0.019084	0.697631	5.840533
19	6	0	0.019704	-0.693558	5.840996
20	6	0	0.039605	-1.399992	4.633899
21	7	0	-4.385538	1.211655	-0.559154
22	7	0	-5.463337	0.486130	-0.971898
23	6	0	-5.372941	-0.839344	-0.860676
24	7	0	-4.336907	-1.531214	-0.377782
25	7	0	4.385411	-1.212037	-0.558636
26	7	0	5.463328	-0.486812	-0.971612
27	6	0	5.372943	0.838738	-0.861283
28	7	0	4.336873	1.530954	-0.378971
29	6	0	4.499762	-2.616873	-0.797020
30	6	0	3.465491	-3.315821	-1.424005
31	6	0	3.606068	-4.678455	-1.671946
32	6	0	4.774981	-5.343722	-1.307366
33	6	0	5.810398	-4.636550	-0.697529
34	6	0	5.678542	-3.275900	-0.441601
35	6	0	6.554963	1.604762	-1.342658
36	6	0	6.587377	2.996986	-1.195130
37	6	0	7.686124	3.727017	-1.637203
38	6	0	8.766371	3.078541	-2.233038
39	6	0	8.739970	1.692673	-2.385559
40	6	0	7.643616	0.959388	-1.944789
41	6	0	-4.500034	2.616378	-0.798213
42	6	0	-5.678676	3.275540	-0.442614
43	6	0	-5.810696	4.636055	-0.699184
44	6	0	-4.775566	5.342941	-1.309840
45	6	0	-3.606788	4.677529	-1.674592
46	6	0	-3.466061	3.315027	-1.426011
47	6	0	-6.554822	-1.605728	-1.341822
48	6	0	-6.586748	-2.997972	-1.194373
49	6	0	-7.685354	-3.728337	-1.636247
50	6	0	-8.765957	-3.080179	-2.231782
51	6	0	-8.740051	-1.694291	-2.384207
52	6	0	-7.643831	-0.960675	-1.943656
53	1	0	-0.063804	2.388561	2.028554
54	1	0	0.063859	-2.387188	2.030142
55	1	0	-2.299712	2.436081	0.727478
56	1	0	-2.237252	-2.546291	0.756291
57	1	0	2.299672	-2.435587	0.728965
58	1	0	2.237261	2.546804	0.754493
59	1	0	-0.069119	2.488858	4.634464
60	1	0	0.069421	-2.485673	4.636426
61	1	0	2.569092	-2.791930	-1.734637
62	1	0	2.803640	-5.216263	-2.165951
63	1	0	4.882088	-6.404920	-1.505478
64	1	0	6.726069	-5.146397	-0.416756
65	1	0	6.478853	-2.712947	0.022007
66	1	0	5.741794	3.489719	-0.732376
67	1	0	7.699085	4.805652	-1.515779
68	1	0	9.623050	3.649041	-2.578187
69	1	0	9.576094	1.181272	-2.852171
70	1	0	7.617468	-0.116088	-2.067153
71	1	0	-6.478767	2.712800	0.021640
72	1	0	-6.726259	5.146019	-0.418276
73	1	0	-4.882795	6.404033	-1.508455
74	1	0	-2.804597	5.215116	-2.169222
75	1	0	-2.569781	2.790991	-1.736748
76	1	0	-5.740916	-3.490450	-0.731804
77	1	0	-7.697920	-4.806986	-1.514907
78	1	0	-9.622529	-3.650938	-2.576766
79	1	0	-9.576468	-1.183129	-2.850558
80	1	0	-7.618064	0.114815	-2.065971
81	1	0	0.034677	-1.234613	6.781555
82	1	0	-0.033873	1.239294	6.780750

Table S11. Optimized molecular geometry of the first excited singlet state of **1** (TD-UB3LYP/6-311G*).

Center Number	Atomic Number	Atomic Type	Coordinates (Angstroms) X	Y	Z
1	6	0	-0.004168	1.722628	1.642183
2	6	0	1.210838	0.977036	1.084320
3	6	0	1.231727	-0.369119	1.427929
4	6	0	0.036066	-0.794910	2.281160
5	6	0	-0.020569	1.489864	3.154285
6	6	0	-0.000571	0.129741	3.500350
7	6	0	-1.212698	-0.417148	1.494665
8	6	0	-1.226521	0.977653	1.129885
9	6	0	-2.253595	1.514257	0.402347
10	6	0	-3.315573	0.666452	0.007792
11	6	0	-3.350583	-0.709972	0.417337
12	6	0	-2.255491	-1.230614	1.163516
13	6	0	2.269300	-1.192346	0.984061
14	6	0	3.270989	-0.658870	0.172656
15	6	0	3.307364	0.739717	-0.117687
16	6	0	2.232703	1.534855	0.326217
17	6	0	-0.046845	2.464692	4.140678
18	6	0	-0.038847	2.078068	5.484933
19	6	0	-0.020025	0.729396	5.828375
20	6	0	-0.008815	-0.253700	4.833431
21	7	0	-4.372771	1.072387	-0.766182
22	7	0	-5.411539	0.301048	-1.049512
23	6	0	-5.397387	-0.957351	-0.583486
24	7	0	-4.390314	-1.496201	0.111981
25	7	0	4.320267	-1.411444	-0.392092
26	7	0	5.543823	-0.766634	-0.668036
27	6	0	5.443404	0.524442	-0.895476
28	7	0	4.359317	1.315284	-0.771334
29	6	0	4.328284	-2.797192	-0.552268
30	6	0	3.147988	-3.520098	-0.822919
31	6	0	3.187901	-4.895226	-1.014315
32	6	0	4.392729	-5.595734	-0.948281
33	6	0	5.565604	-4.882738	-0.700701
34	6	0	5.545084	-3.507386	-0.508190
35	6	0	6.711210	1.194185	-1.321657
36	6	0	6.753565	2.587008	-1.450100
37	6	0	7.924681	3.227742	-1.847171
38	6	0	9.072767	2.487137	-2.120482
39	6	0	9.038287	1.097819	-1.998161
40	6	0	7.868094	0.456534	-1.605153
41	6	0	-4.446780	2.398256	-1.345696
42	6	0	-5.549697	3.195438	-1.051996
43	6	0	-5.638323	4.458711	-1.627602
44	6	0	-4.641166	4.907117	-2.492737
45	6	0	-3.550962	4.090792	-2.788463
46	6	0	-3.445821	2.826977	-2.214549
47	6	0	-6.580250	-1.789531	-0.894214
48	6	0	-6.648569	-3.102848	-0.410279
49	6	0	-7.755929	-3.894624	-0.691743
50	6	0	-8.803917	-3.387541	-1.458260
51	6	0	-8.740552	-2.082194	-1.944383
52	6	0	-7.636876	-1.284906	-1.665849
53	1	0	-0.018504	2.780006	1.375654
54	1	0	0.049382	-1.851275	2.551156
55	1	0	-2.255856	2.561973	0.134127
56	1	0	-2.292444	-2.276601	1.443937
57	1	0	2.288390	-2.241554	1.253007
58	1	0	2.252933	2.589663	0.071467
59	1	0	-0.058131	3.517560	3.874471
60	1	0	0.008609	-1.305317	5.103827
61	1	0	2.207125	-2.993662	-0.922883
62	1	0	2.264494	-5.423213	-1.235868
63	1	0	4.417614	-6.669948	-1.098678
64	1	0	6.516768	-5.405409	-0.653290
65	1	0	6.455848	-2.956393	-0.323347
66	1	0	5.851913	3.145694	-1.233375
67	1	0	7.941038	4.309860	-1.941514
68	1	0	9.986640	2.986461	-2.429195
69	1	0	9.926794	0.511463	-2.214031
70	1	0	7.831803	-0.622186	-1.518856
71	1	0	-6.319704	2.828002	-0.384524
72	1	0	-6.488152	5.092040	-1.399497
73	1	0	-4.716403	5.891426	-2.941239
74	1	0	-2.782001	4.431269	-3.472328
75	1	0	-2.610212	2.179551	-2.453340
76	1	0	-5.829899	-3.491015	0.181951
77	1	0	-7.800335	-4.910058	-0.313092
78	1	0	-9.666500	-4.007851	-1.678003
79	1	0	-9.552462	-1.685224	-2.544279
80	1	0	-7.585749	-0.273168	-2.047874
81	1	0	-0.009749	0.437163	6.873390
82	1	0	-0.043056	2.834831	6.262640

Table S12. Optimized molecular geometry of the first excited triplet state of **1** (TD-UB3LYP/6-311G*).

Center Number	Atomic Number	Atomic Type	Coordinates (Angstroms) X	Y	Z
1	6	0	-0.045756	1.308343	2.097264
2	6	0	1.181519	0.741494	1.390413
3	6	0	1.216531	-0.670208	1.394540
4	6	0	0.025983	-1.295805	2.113173
5	6	0	-0.061517	0.715874	3.503507
6	6	0	-0.021314	-0.687168	3.511911
7	6	0	-1.203213	-0.762411	1.384905
8	6	0	-1.237511	0.697502	1.369901
9	6	0	-2.242805	1.375823	0.750127
10	6	0	-3.278193	0.634493	0.112897
11	6	0	-3.296804	-0.835304	0.169606
12	6	0	-2.190629	-1.492340	0.811281
13	6	0	2.242379	-1.356362	0.769783
14	6	0	3.258886	-0.630786	0.127107
15	6	0	3.262572	0.793712	0.156516
16	6	0	2.189019	1.460504	0.784115
17	6	0	-0.122313	1.421847	4.697545
18	6	0	-0.136702	0.722821	5.907384
19	6	0	-0.097375	-0.669543	5.915678
20	6	0	-0.042791	-1.381091	4.714306
21	7	0	-4.310800	1.182761	-0.574680
22	7	0	-5.323872	0.528987	-1.090012
23	6	0	-5.329243	-0.862990	-0.911123
24	7	0	-4.297449	-1.518266	-0.331423
25	7	0	4.329925	-1.222378	-0.540544
26	7	0	5.397750	-0.501601	-0.982689
27	6	0	5.311272	0.825093	-0.879042
28	7	0	4.285177	1.520220	-0.379362
29	6	0	4.440591	-2.630715	-0.767706
30	6	0	3.401659	-3.331513	-1.383954
31	6	0	3.537881	-4.696609	-1.620867
32	6	0	4.706853	-5.361143	-1.255174
33	6	0	5.746630	-4.651737	-0.655515
34	6	0	5.619157	-3.288485	-0.410935
35	6	0	6.482546	1.587070	-1.390634
36	6	0	6.517628	2.980542	-1.255333
37	6	0	7.606936	3.706792	-1.725883
38	6	0	8.674818	3.053278	-2.338307
39	6	0	8.645679	1.666178	-2.478650
40	6	0	7.558818	0.936538	-2.009421
41	6	0	-4.380652	2.614403	-0.811258
42	6	0	-5.462788	3.322487	-0.299373
43	6	0	-5.550137	4.689959	-0.543615
44	6	0	-4.571516	5.331294	-1.301741
45	6	0	-3.501432	4.604601	-1.820586
46	6	0	-3.399007	3.237269	-1.575377
47	6	0	-6.467324	-1.582768	-1.417235
48	6	0	-6.548362	-2.992778	-1.290429
49	6	0	-7.644821	-3.687071	-1.770808
50	6	0	-8.704215	-3.016227	-2.394895
51	6	0	-8.637702	-1.627701	-2.528289
52	6	0	-7.545668	-0.916237	-2.053107
53	1	0	-0.066579	2.398876	2.095525
54	1	0	0.061968	-2.385724	2.125235
55	1	0	-2.263323	2.458680	0.748834
56	1	0	-2.202366	-2.576876	0.829005
57	1	0	2.266040	-2.438495	0.785352
58	1	0	2.205880	2.545035	0.782890
59	1	0	-0.155416	2.507469	4.692998
60	1	0	-0.016309	-2.466799	4.722685
61	1	0	2.504962	-2.807924	-1.694426
62	1	0	2.732192	-5.236931	-2.106595
63	1	0	4.810531	-6.424241	-1.444537
64	1	0	6.662052	-5.161599	-0.374178
65	1	0	6.422749	-2.723470	0.044573
66	1	0	5.681860	3.477389	-0.779345
67	1	0	7.622313	4.786366	-1.613714
68	1	0	9.524127	3.620856	-2.705654
69	1	0	9.472207	1.150963	-2.957888
70	1	0	7.530724	-0.139934	-2.122136
71	1	0	-6.221503	2.801605	0.271791
72	1	0	-6.386724	5.252868	-0.144351
73	1	0	-4.646962	6.396094	-1.494654
74	1	0	-2.747825	5.097885	-2.424683
75	1	0	-2.579728	2.657908	-1.984948
76	1	0	-5.734226	-3.520164	-0.808810
77	1	0	-7.680402	-4.767216	-1.659858
78	1	0	-9.561435	-3.566335	-2.768771
79	1	0	-9.449758	-1.092232	-3.012350
80	1	0	-7.507851	0.159419	-2.170717
81	1	0	-0.109288	-1.205669	6.859075
82	1	0	-0.178440	1.268581	6.844414

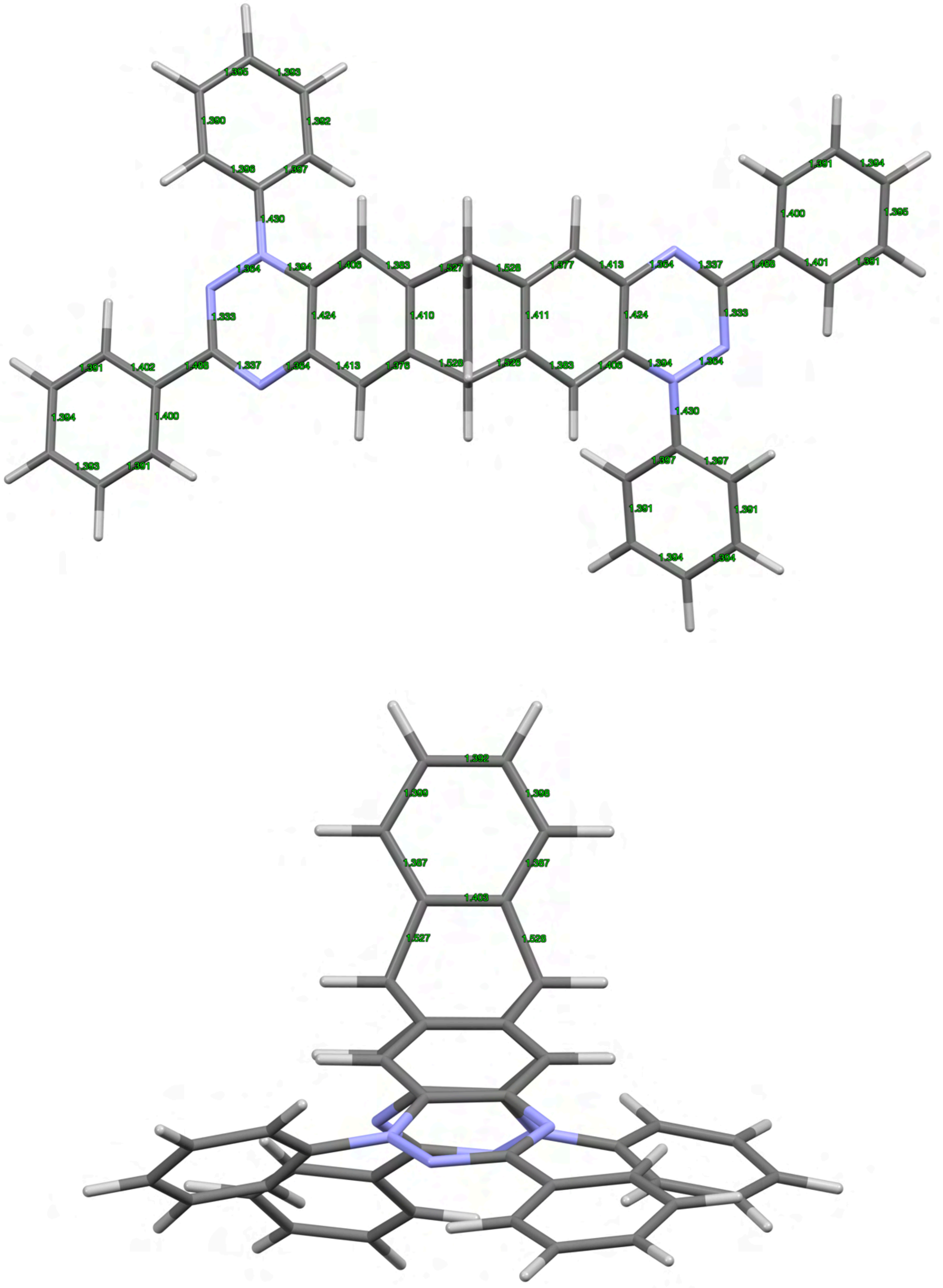

Figure S26. Bond lengths of the optimized molecular geometry of the lowest singlet state of **1** (UB3LYP/6-311G*).

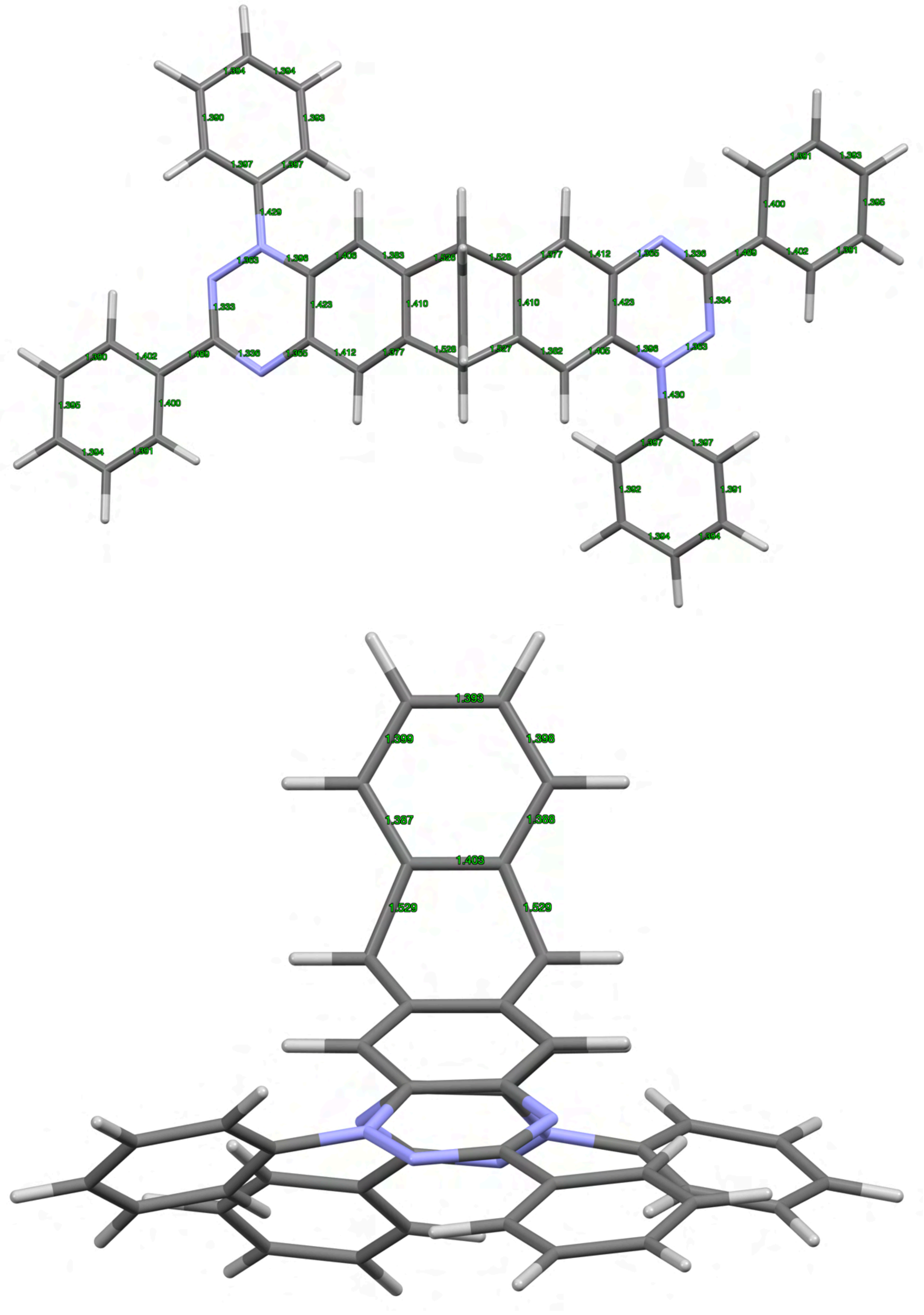

Figure S27. Bond lengths of the optimized molecular geometry of the lowest triplet state of **1** (UB3LYP/6-311G*).

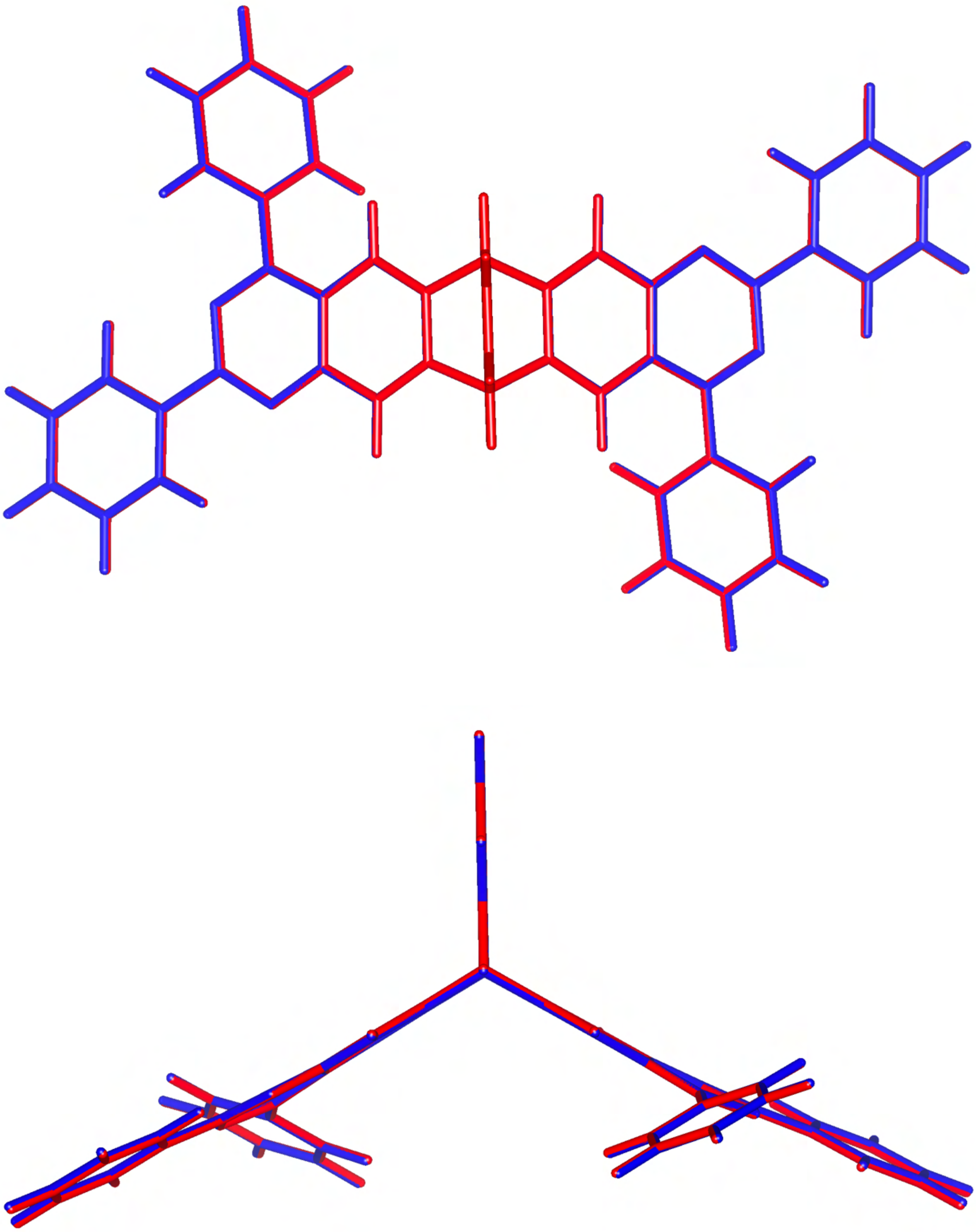

Figure S28. Overlay of the energy-minimized structures of **1** optimized at the lowest singlet (blue) and triplet (red) states (UB3LYP/6-311G*). The drawing was generated with the VESTA program.[S3]

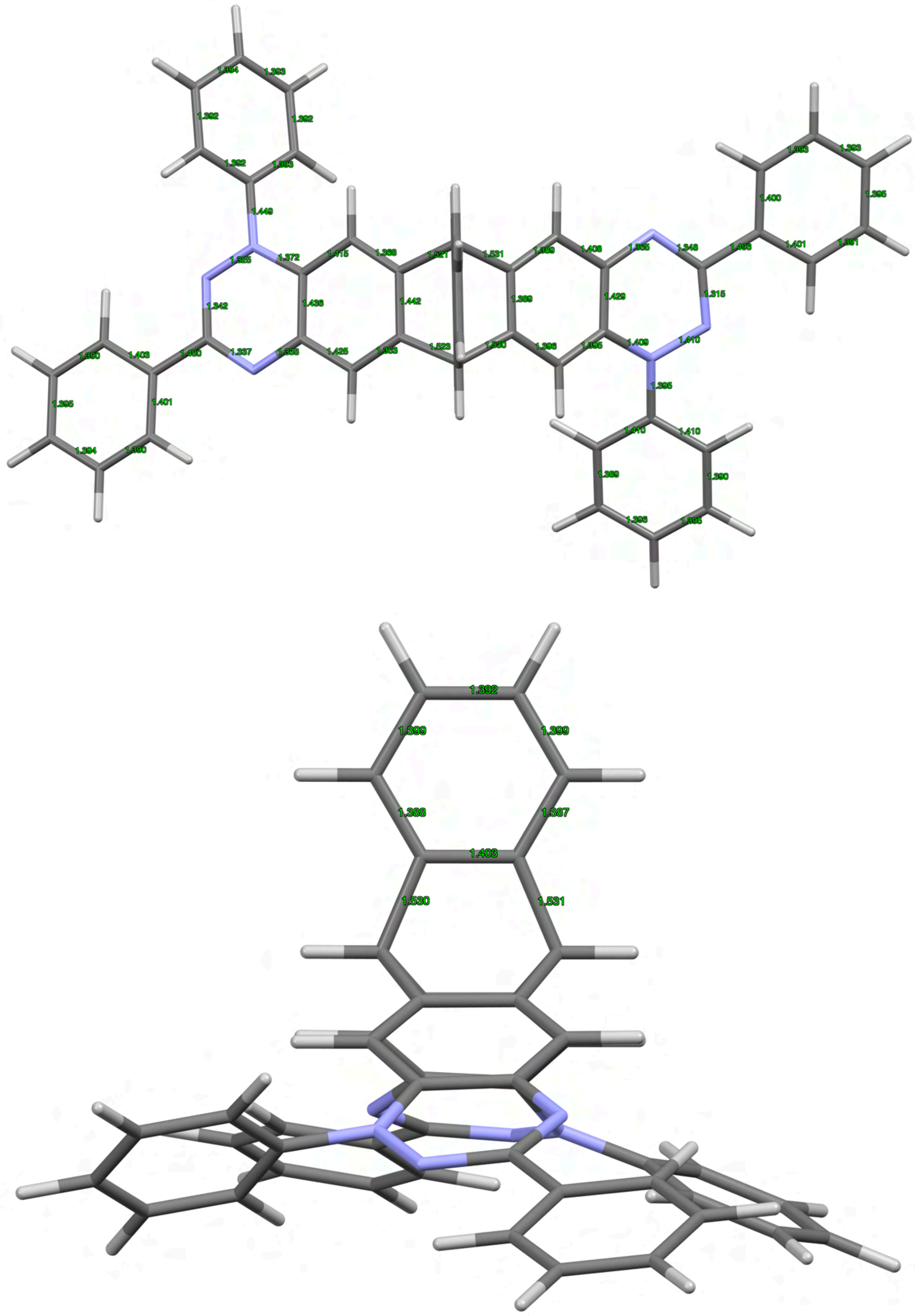

Figure S29. Bond lengths of the optimized molecular geometry of the first excited singlet state of **1** (UB3LYP/6-311G*).

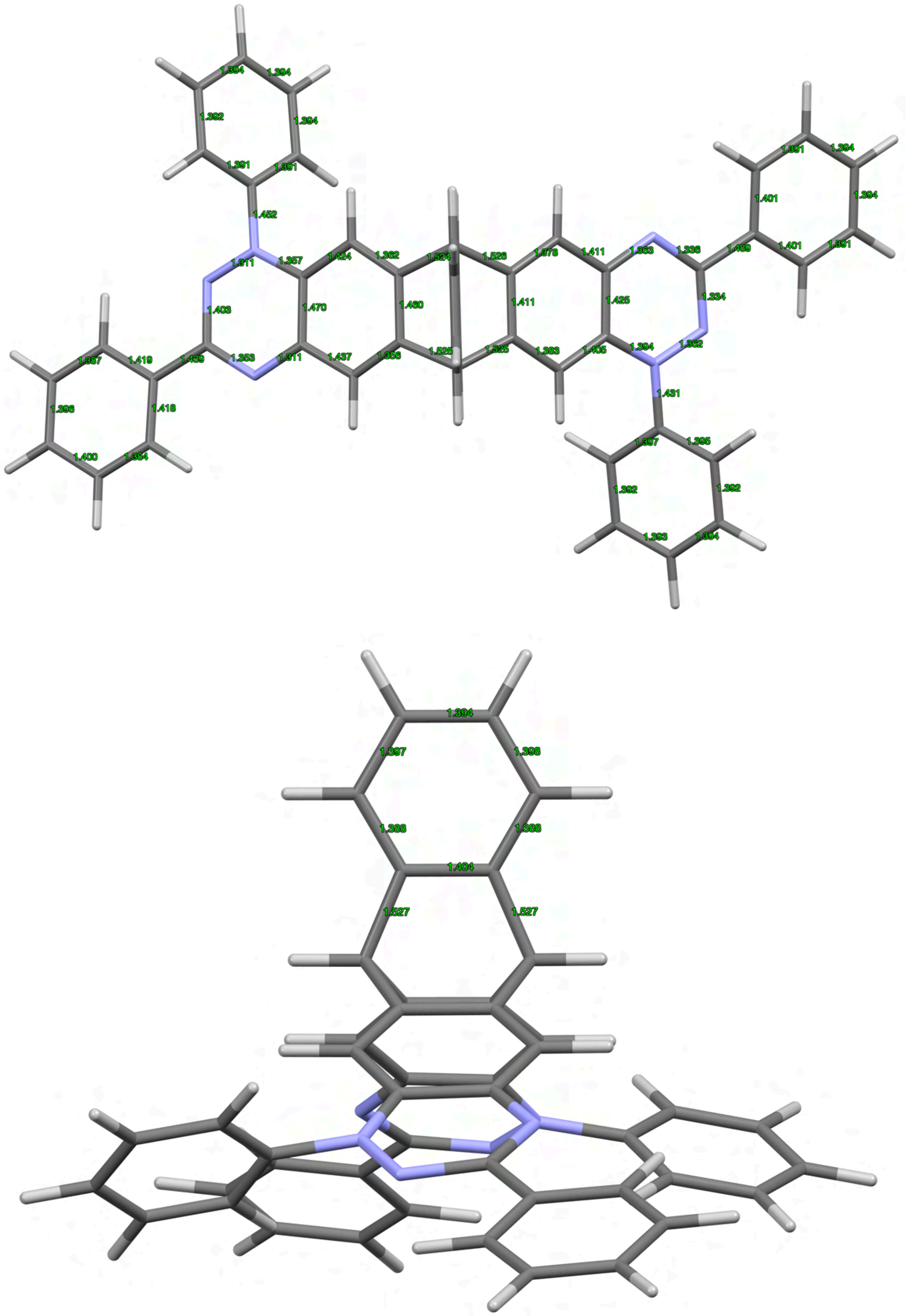

Figure S30. Bond lengths of the optimized molecular geometry of the first excited triplet state of **1** (UB3LYP/6-311G*).

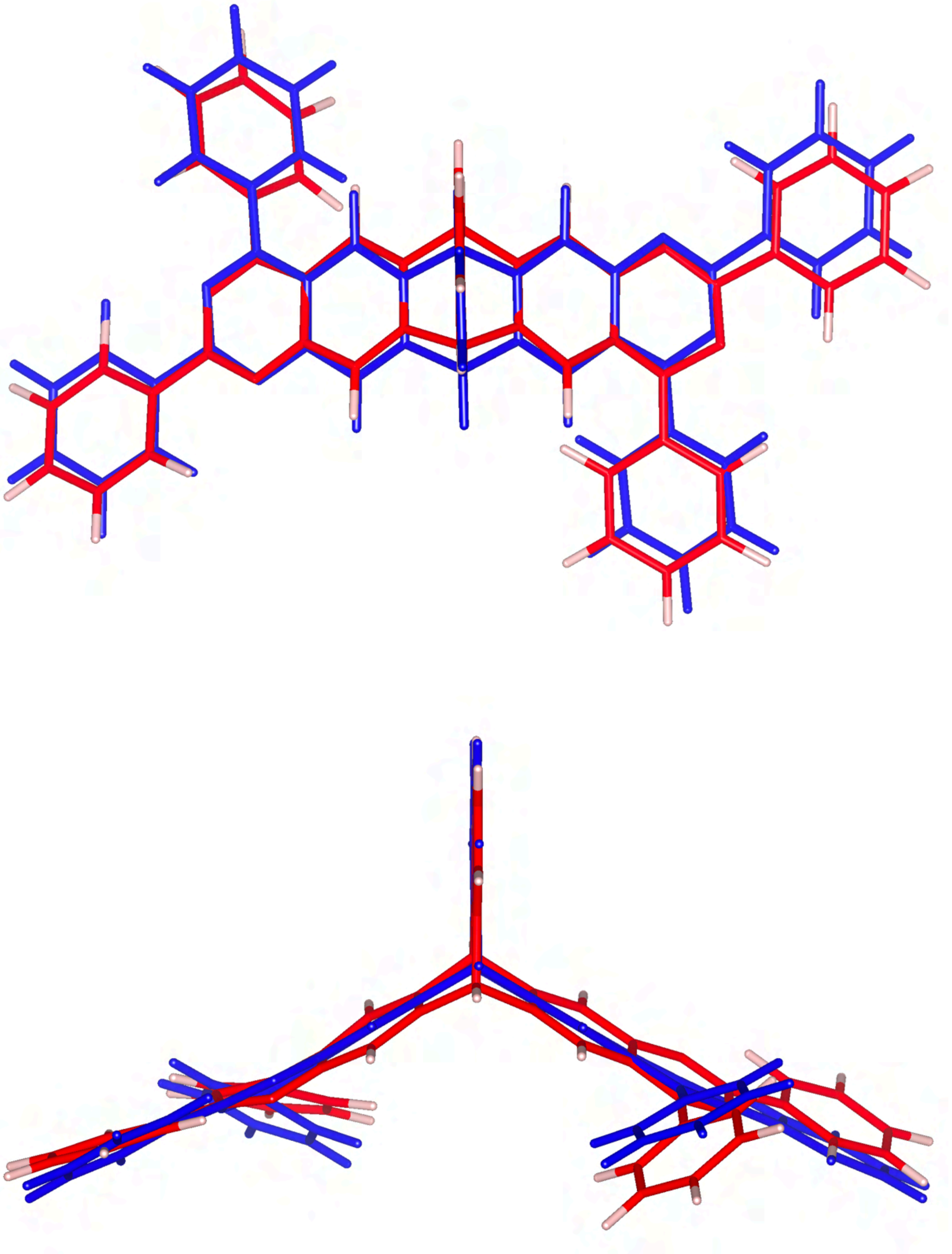

Figure S31. Overlay of the energy-minimized structures of **1** optimized at the ground singlet (blue) and the first excited singlet (red) states (UB3LYP/6-311G*). The drawing was generated with the VESTA program.[S3]

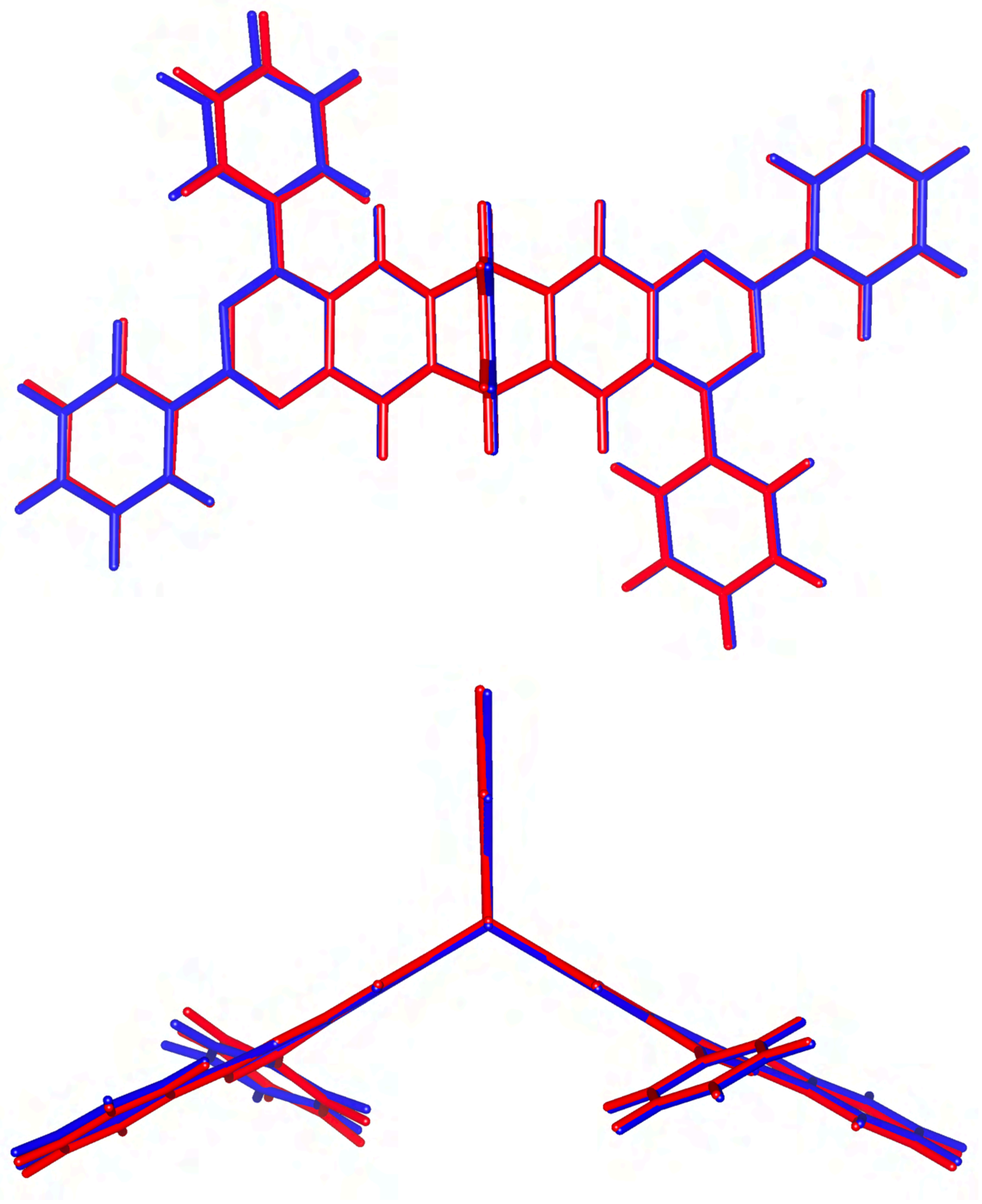

Figure S32. Overlay of the energy-minimized structures of **1** optimized at the lowest triplet (blue) and the first excited triplet (red) states (UB3LYP/6-311G*). The drawing was generated with the VESTA program.[S3]

Table S13. Calculated excitations of monomer **6** at the lowest doublet state geometry (UB3LYP/6-311G*).

```
Excited State   1:  2.049-A     2.1564 eV  574.95 nm  f=0.0013  <S**2>=0.799
    75A -> 76A       0.94701
    74B -> 75B       0.24641

 Excited State   2:  2.120-A     2.6083 eV  475.34 nm  f=0.0134  <S**2>=0.874
    75A -> 76A      -0.20556
    75A -> 77A       0.24075
    75A -> 81A       0.11590
    72B -> 75B       0.25526
    73B -> 75B       0.16911
    74B -> 75B       0.85914

 Excited State   3:  2.051-A     2.7873 eV  444.82 nm  f=0.0012  <S**2>=0.802
    75A -> 77A      -0.15356
    70B -> 75B       0.13411
    71B -> 75B       0.77389
    72B -> 75B       0.46864
    73B -> 75B       0.24843
    74B -> 75B      -0.20570

 Excited State   4:  2.155-A     2.9381 eV  421.99 nm  f=0.0676  <S**2>=0.911
    75A -> 77A       0.91603
    69B -> 75B       0.11492
    71B -> 75B       0.12511
    74B -> 75B      -0.22087

 Excited State   5:  2.124-A     3.2436 eV  382.24 nm  f=0.0072  <S**2>=0.877
    75A -> 78A       0.96048
    70B -> 75B      -0.11191
```

Table S14. Calculated excitations of monomer **6** at the first excited doublet state geometry (UB3LYP/6-311G*).

```
Excited State   1:  2.015-A     1.2546 eV  988.25 nm  f=0.0010  <S**2>=0.765
    75A -> 76A       0.98435

 Excited State   2:  2.062-A     2.5258 eV  490.88 nm  f=0.0310  <S**2>=0.813
    75A -> 77A       0.96900
    74B -> 75B      -0.16404

 Excited State   3:  2.061-A     2.7327 eV  453.70 nm  f=0.0056  <S**2>=0.812
    75A -> 78A       0.98719

 Excited State   4:  3.054-A     2.7825 eV  445.59 nm  f=0.0181  <S**2>=2.081
    74A -> 76A      -0.48621
    75A -> 77A       0.11407
    68B -> 80B      -0.10773
    71B -> 79B      -0.11552
    72B -> 76B       0.16052
    74B -> 75B       0.68070
    74B -> 76B       0.38310

 Excited State   5:  2.107-A     2.9788 eV  416.22 nm  f=0.0011  <S**2>=0.860
    73B -> 75B       0.96680
    73B -> 76B      -0.11649
    73B -> 81B       0.10812
```

Table S15. Calculated excitations of **1** (singlet) at the lowest singlet state geometry (UB3LYP/6-311G*).

```
Excited State   1:  1.235-A      1.5437 eV  803.17 nm  f=0.0002  <S**2>=0.131
    174A -> 175A       0.70530
    174B -> 175B       0.70385

 Excited State   2:  1.133-A      1.6307 eV  760.30 nm  f=0.1123  <S**2>=0.071
    174A -> 175A      -0.70361
    174B -> 175B       0.70505

 Excited State   3:  2.316-A      2.0555 eV  603.17 nm  f=0.0002  <S**2>=1.091
    173A -> 175A      -0.11479
    174A -> 176A       0.65691
    174A -> 177A       0.19582
    173B -> 175B      -0.11587
    174B -> 176B       0.65155
    174B -> 177B       0.19413

 Excited State   4:  2.266-A      2.0671 eV  599.80 nm  f=0.0003  <S**2>=1.033
    173A -> 175A      -0.13279
    174A -> 176A      -0.64092
    174A -> 177A      -0.21280
    173B -> 175B       0.13182
    174B -> 176B       0.64632
    174B -> 177B       0.21449

 Excited State   5:  2.358-A      2.6324 eV  471.00 nm  f=0.0018  <S**2>=1.140
    168A -> 175A       0.14941
    173A -> 175A       0.60173
    174A -> 176A       0.10870
    174A -> 178A       0.22460
    168B -> 175B      -0.14491
    173B -> 175B       0.58322
    174B -> 176B       0.11234
    174B -> 178B       0.23223

Excited State   6:  2.339-A      2.6374 eV  470.10 nm  f=0.0198  <S**2>=1.117
    168A -> 175A      -0.14090
    173A -> 175A      -0.58643
    174A -> 176A       0.11836
    174A -> 178A       0.25493
    168B -> 175B      -0.14540
    173B -> 175B       0.60480
    174B -> 176B      -0.11494
    174B -> 178B      -0.24772
```

Table S16. Calculated excitations of **1** (triplet) at the lowest triplet state geometry (UB3LYP/6-311G*).

```
Excited State   1:  3.045-A     2.0014 eV  619.49 nm  f=0.0002  <S**2>=2.068
    174A -> 177A        0.47095
    175A -> 176A        0.84660
    172B -> 174B       -0.10999
    173B -> 175B        0.10620

 Excited State   2:  3.041-A     2.0634 eV  600.88 nm  f=0.0027  <S**2>=2.062
    174A -> 176A        0.61412
    175A -> 177A        0.73582
    172B -> 175B       -0.11251
    173B -> 174B        0.16561

 Excited State   3:  3.080-A     2.5842 eV  479.78 nm  f=0.0018  <S**2>=2.121
    174A -> 178A       -0.19758
    175A -> 176A        0.18031
    175A -> 179A       -0.24587
    167B -> 175B        0.13012
    168B -> 174B       -0.13837
    170B -> 174B       -0.11799
    171B -> 175B       -0.11585
    172B -> 174B        0.74997
    173B -> 175B       -0.41585

 Excited State   4:  3.079-A     2.6046 eV  476.02 nm  f=0.0100  <S**2>=2.120
    174A -> 176A       -0.16159
    174A -> 179A        0.21237
    175A -> 178A        0.42038
    167B -> 174B       -0.14853
    171B -> 174B        0.12253
    172B -> 175B       -0.44546
    173B -> 174B        0.66997

 Excited State   5:  3.074-A     2.7556 eV  449.93 nm  f=0.2017  <S**2>=2.113
    174A -> 176A       -0.13257
    174A -> 179A        0.30116
    175A -> 177A        0.26563
    175A -> 178A        0.72808
    165B -> 174B        0.10320
    167B -> 174B        0.15761
    171B -> 174B       -0.14163
    172B -> 175B        0.23244
    173B -> 174B       -0.30510
```

Table S17. Calculated excitations of **1** (singlet) at the first singlet excited-state geometry (UB3LYP/6-311G*).

```
Excited State   1:  1.160-A      0.9727 eV 1274.63 nm  f=0.0661  <S**2>=0.086
   174A -> 175A       0.99759

 Excited State   2:  2.277-A      1.7041 eV  727.56 nm  f=0.0008  <S**2>=1.046
   174A -> 176A       0.97433
   173B -> 175B       0.10063

 Excited State   3:  1.262-A      2.0797 eV  596.17 nm  f=0.0445  <S**2>=0.148
   173A -> 175A       0.13669
   174B -> 175B       0.98214

 Excited State   4:  2.298-A      2.1602 eV  573.95 nm  f=0.0552  <S**2>=1.070
   165A -> 175A      -0.11194
   173A -> 175A       0.92448
   174B -> 175B      -0.14228
   174B -> 177B      -0.19146
   174B -> 178B      -0.10096

 Excited State   5:  2.313-A      2.4380 eV  508.55 nm  f=0.0038  <S**2>=1.088
   170A -> 175A      -0.18937
   171A -> 175A       0.31189
   172A -> 175A       0.14936
   173A -> 175A       0.11971
   174A -> 177A      -0.10117
   174B -> 176B       0.47569
   174B -> 177B       0.71465
```

Table S18. Calculated excitations of **1** (triplet) at the first triplet excited-state geometry (UB3LYP/6-311G*).

```
Excited State   1:  3.022-A     1.1587 eV 1070.06 nm  f=0.0006  <S**2>=2.034
    174A -> 176A      0.16770
    175A -> 176A      0.96945

 Excited State   2:  3.044-A     1.9666 eV  630.46 nm  f=0.0008  <S**2>=2.066
    174A -> 176A      0.26338
    174A -> 177A      0.67882
    175A -> 177A     -0.63966
    172B -> 174B     -0.12989

 Excited State   3:  3.036-A     2.3408 eV  529.66 nm  f=0.0065  <S**2>=2.055
    174A -> 176A      0.69418
    174A -> 177A      0.28201
    175A -> 177A      0.61033
    175A -> 178A     -0.12236
    175A -> 179A     -0.15415

 Excited State   4:  3.049-A     2.4043 eV  515.68 nm  f=0.0347  <S**2>=2.074
    174A -> 176A      0.53646
    174A -> 177A     -0.37461
    175A -> 177A     -0.20510
    175A -> 178A      0.42105
    175A -> 179A      0.51774
    172B -> 174B      0.15969

 Excited State   5:  3.043-A     2.4249 eV  511.30 nm  f=0.0229  <S**2>=2.065
    174A -> 176A     -0.31800
    174A -> 177A      0.45550
    174A -> 179A      0.11191
    175A -> 176A      0.11744
    175A -> 177A      0.38358
    175A -> 178A      0.48793
    175A -> 179A      0.46315
    172B -> 174B     -0.17423
```

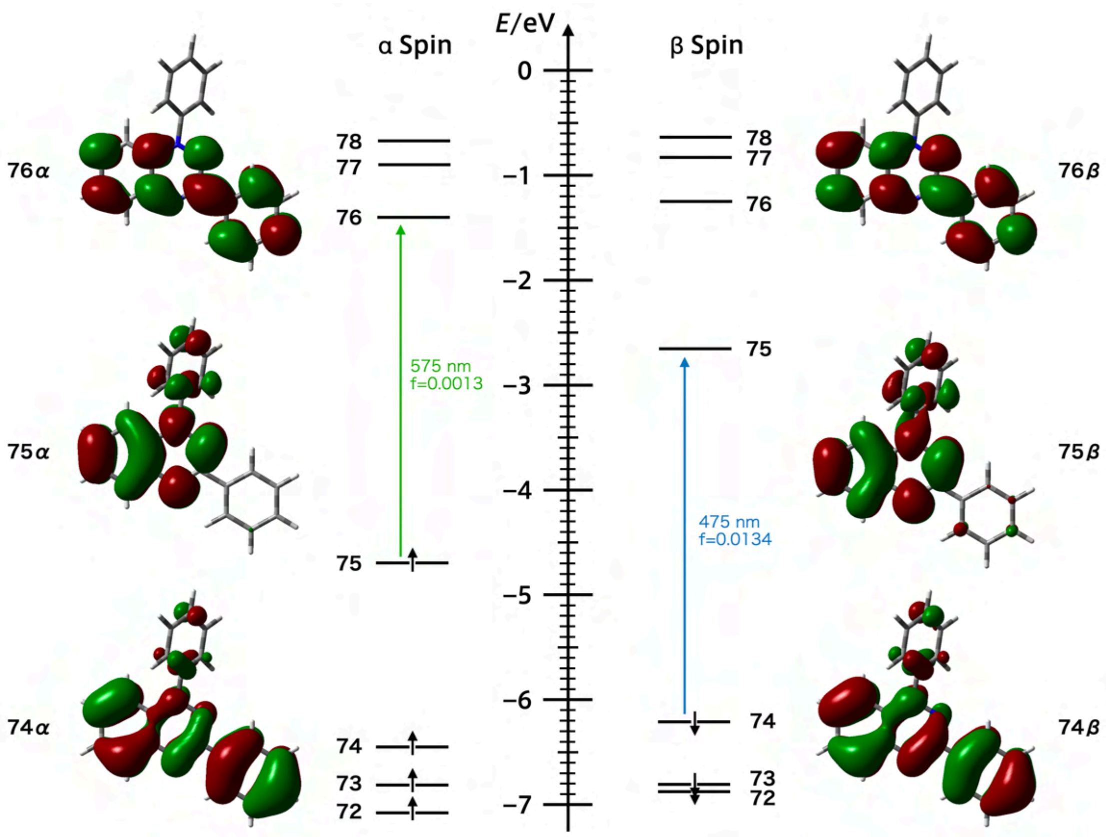

Figure S33. MO diagram of Blatter radical **6** at the lowest doublet state geometry (UB3LYP/6-311G*).

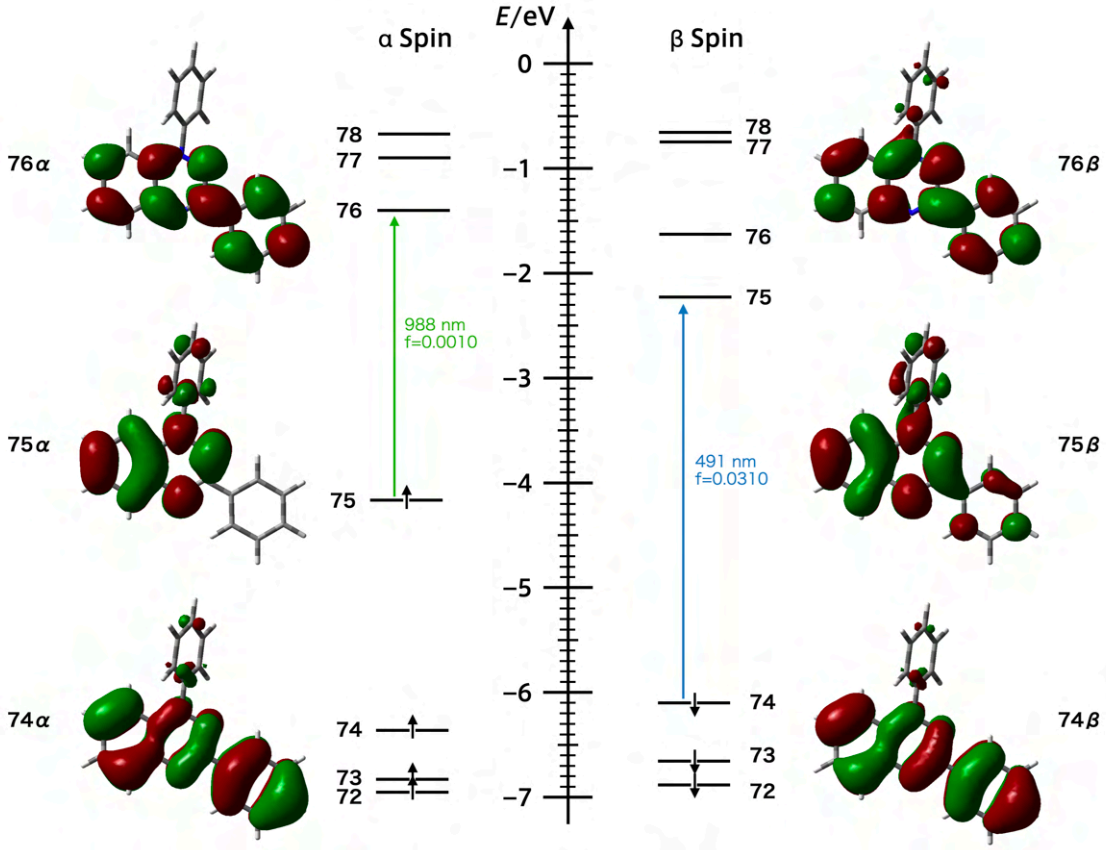

Figure S34. MO diagram of Blatter radical **6** at the lowest doublet excited-state geometry (UB3LYP/6-311G*).

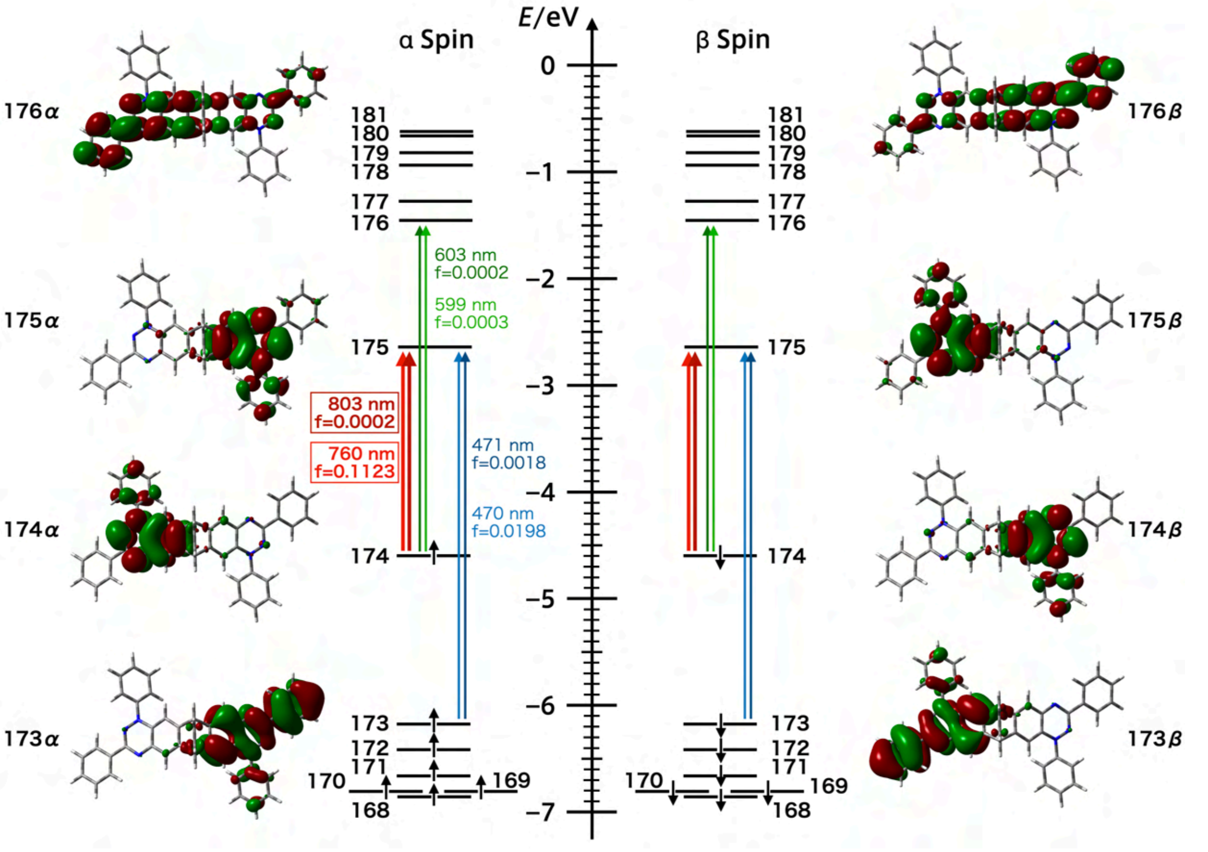

Figure S35. MO diagram of **1** at the lowest singlet state geometry (UB3LYP/6-311G*).

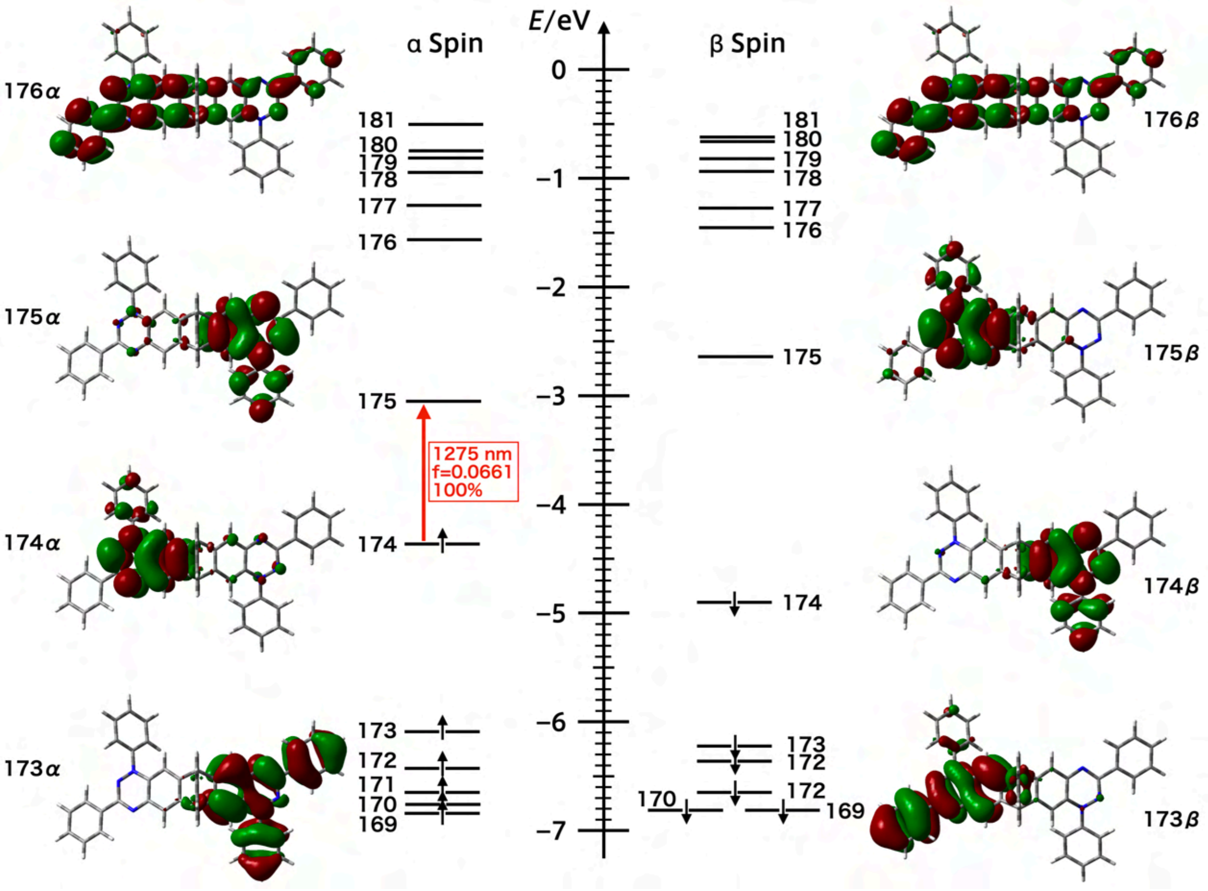

Figure S36. MO diagram of **1** at the lowest singlet excited-state geometry (UB3LYP/6-311G*).

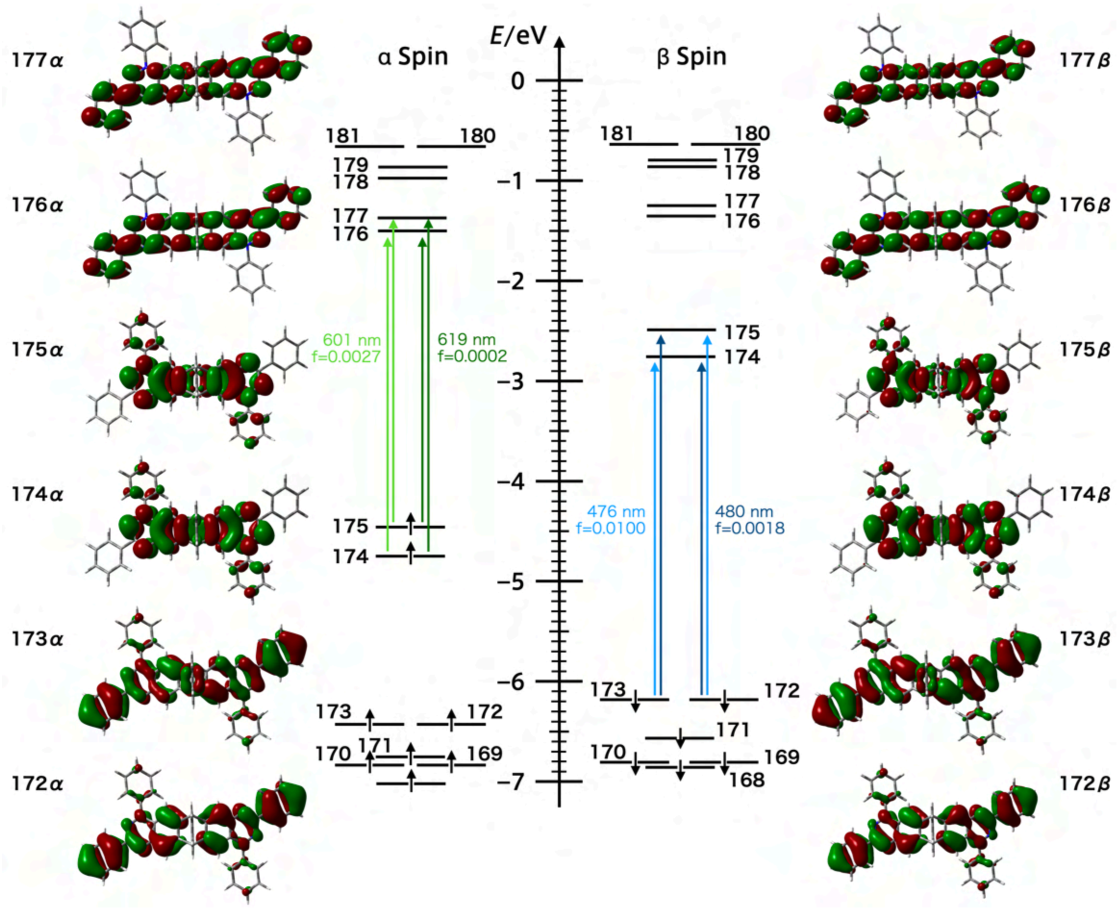

Figure S37. MO diagram of **1** at the lowest triplet state geometry (UB3LYP/6-311G*).

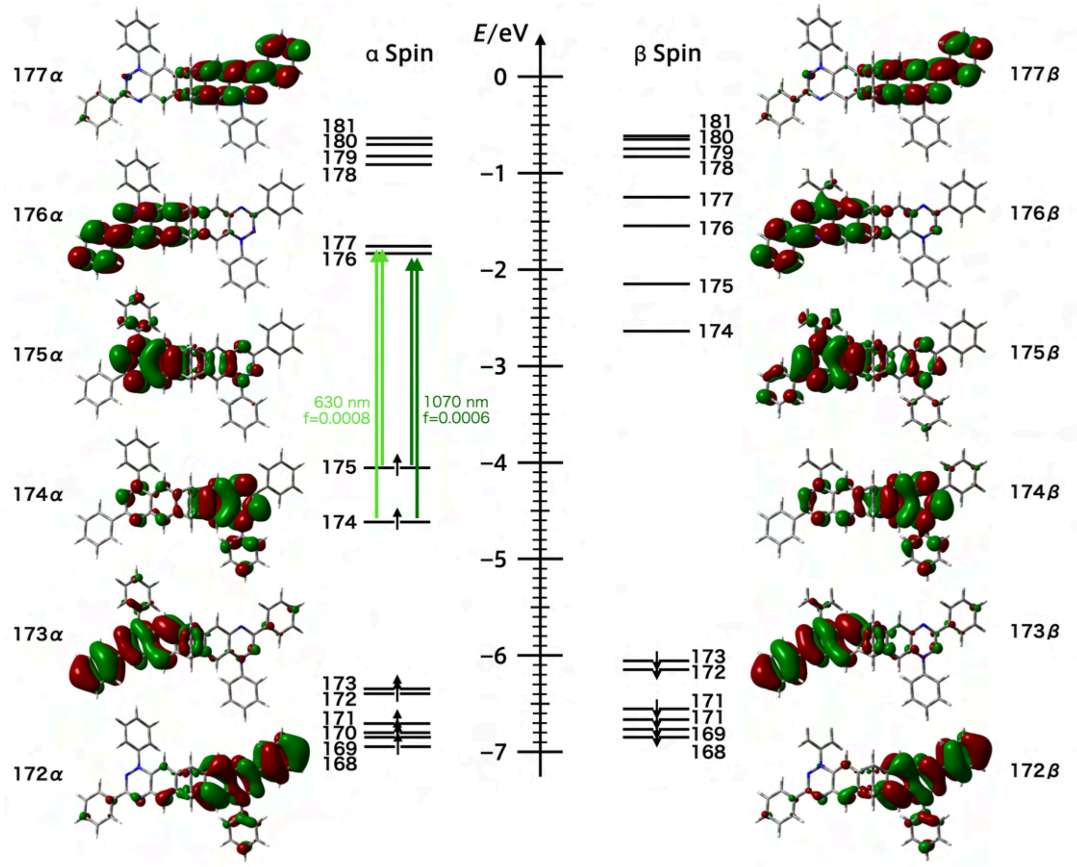

Figure S38. MO diagram of **1** at the lowest triplet excited-state geometry (UB3LYP/6-311G*).

Table S19. Optimized geometry of the lowest singlet state of **1** (UB3LYP/6-311G*-IEFPCM(toluene)).

Center Number	Atomic Number	Atomic Type	Coordinates (Angstroms) X	Y	Z
1	6	0	0.038967	-1.298766	2.077174
2	6	0	-1.192552	-0.742265	1.365695
3	6	0	-1.231651	0.668685	1.361957
4	6	0	-0.038967	1.298781	2.077165
5	6	0	0.021938	-0.701225	3.483419
6	6	0	-0.021938	0.701250	3.483414
7	6	0	1.192552	0.742274	1.365689
8	6	0	1.231651	-0.668675	1.361962
9	6	0	2.265895	-1.349146	0.746917
10	6	0	3.284141	-0.616112	0.113292
11	6	0	3.281121	0.807294	0.146205
12	6	0	2.202818	1.468367	0.773840
13	6	0	-2.265895	1.349151	0.746909
14	6	0	-3.284142	0.616112	0.113289
15	6	0	-3.281121	-0.807293	0.146210
16	6	0	-2.202817	-1.468361	0.773850
17	6	0	0.044166	-1.402352	4.680987
18	6	0	0.021902	-0.695937	5.888226
19	6	0	-0.021902	0.695979	5.888221
20	6	0	-0.044166	1.402386	4.680977
21	7	0	4.360077	-1.203568	-0.549206
22	7	0	5.423597	-0.476694	-0.995479
23	6	0	5.329808	0.848212	-0.891987
24	7	0	4.302316	1.539901	-0.388790
25	7	0	-4.360078	1.203564	-0.549212
26	7	0	-5.423598	0.476686	-0.995481
27	6	0	-5.329807	-0.848219	-0.891982
28	7	0	-4.302315	-1.539904	-0.388781
29	6	0	-4.480749	2.613438	-0.766707
30	6	0	-3.461827	3.317309	-1.411943
31	6	0	-3.606930	4.683639	-1.638466
32	6	0	-4.765110	5.345079	-1.233346
33	6	0	-5.784834	4.632459	-0.602983
34	6	0	-5.647690	3.267932	-0.368061
35	6	0	-6.496385	-1.616323	-1.409440
36	6	0	-6.518723	-3.012059	-1.292771
37	6	0	-7.602179	-3.743420	-1.770613
38	6	0	-8.678354	-3.092583	-2.372069
39	6	0	-8.662882	-1.703012	-2.493537
40	6	0	-7.581526	-0.969054	-2.017024
41	6	0	4.480746	-2.613443	-0.766694
42	6	0	5.647688	-3.267936	-0.368045
43	6	0	5.784831	-4.632464	-0.602962
44	6	0	4.765106	-5.345086	-1.233321
45	6	0	3.606926	-4.683648	-1.638444
46	6	0	3.461824	-3.317316	-1.411928
47	6	0	6.496387	1.616312	-1.409448
48	6	0	6.518725	3.012049	-1.292789
49	6	0	7.602182	3.743406	-1.770633
50	6	0	8.678359	3.092565	-2.372083
51	6	0	8.662887	1.702993	-2.493540
52	6	0	7.581530	0.969038	-2.017024
53	1	0	0.070576	-2.388362	2.077541
54	1	0	-0.070575	2.388377	2.077523
55	1	0	2.293267	-2.431092	0.759108
56	1	0	2.213617	2.552930	0.781295
57	1	0	-2.293268	2.431098	0.759091
58	1	0	-2.213616	-2.552925	0.781312
59	1	0	0.077668	-2.487823	4.682765
60	1	0	-0.077668	2.487857	4.682748
61	1	0	-2.573727	2.796567	-1.750623
62	1	0	-2.817100	5.227244	-2.145804
63	1	0	-4.875841	6.408774	-1.414730
64	1	0	-6.690773	5.140251	-0.289537
65	1	0	-6.435101	2.702331	0.114538
66	1	0	-5.678107	-3.508957	-0.825525
67	1	0	-7.606141	-4.824385	-1.672718
68	1	0	-9.522820	-3.663495	-2.744972
69	1	0	-9.495639	-1.189189	-2.963188
70	1	0	-7.566245	0.109069	-2.115228
71	1	0	6.435099	-2.702334	0.114551
72	1	0	6.690769	-5.140256	-0.289513
73	1	0	4.875836	-6.408782	-1.414701
74	1	0	2.817096	-5.227254	-2.145780
75	1	0	2.573725	-2.796575	-1.750610
76	1	0	5.678108	3.508951	-0.825548
77	1	0	7.606144	4.824372	-1.672747
78	1	0	9.522825	3.663474	-2.744988
79	1	0	9.495645	1.189167	-2.963184
80	1	0	7.566249	-0.109085	-2.115220
81	1	0	-0.038800	1.237029	6.828717
82	1	0	0.038799	-1.236981	6.828726

Table S20. Optimized geometry of the lowest triplet state of **1** (UB3LYP/6-311G*-IEFPCM(toluene)).

Center Number	Atomic Number	Atomic Type	Coordinates (Angstroms) X	Y	Z
1	6	0	0.039569	-1.298576	2.040826
2	6	0	-1.195419	-0.742372	1.334389
3	6	0	-1.235625	0.668100	1.331038
4	6	0	-0.039568	1.298588	2.040819
5	6	0	0.022023	-0.701310	3.448100
6	6	0	-0.022020	0.701330	3.448096
7	6	0	1.195418	0.742379	1.334382
8	6	0	1.235624	-0.668092	1.331039
9	6	0	2.276618	-1.347774	0.727139
10	6	0	3.301569	-0.613804	0.105111
11	6	0	3.297131	0.808251	0.138075
12	6	0	2.211542	1.468991	0.753622
13	6	0	-2.276620	1.347778	0.727135
14	6	0	-3.301572	0.613804	0.105114
15	6	0	-3.297135	-0.808250	0.138087
16	6	0	-2.211544	-1.468987	0.753635
17	6	0	0.044224	-1.402389	4.645383
18	6	0	0.021922	-0.695820	5.852854
19	6	0	-0.021916	0.695854	5.852850
20	6	0	-0.044219	1.402416	4.645375
21	7	0	4.385838	-1.201531	-0.546379
22	7	0	5.451436	-0.473050	-0.983349
23	6	0	5.355195	0.852593	-0.880964
24	7	0	4.323445	1.543134	-0.386916
25	7	0	-4.385841	1.201527	-0.546379
26	7	0	-5.451440	0.473045	-0.983345
27	6	0	-5.355202	-0.852598	-0.880947
28	7	0	-4.323451	-1.543137	-0.386898
29	6	0	-4.510689	2.611185	-0.761516
30	6	0	-3.498613	3.317711	-1.414718
31	6	0	-3.648190	4.683987	-1.638667
32	6	0	-4.804174	5.342877	-1.223290
33	6	0	-5.817262	4.627668	-0.585185
34	6	0	-5.675534	3.263226	-0.352635
35	6	0	-6.525632	-1.621172	-1.389011
36	6	0	-6.545273	-3.017143	-1.274540
37	6	0	-7.632205	-3.748943	-1.743638
38	6	0	-8.714684	-3.098337	-2.333990
39	6	0	-8.701967	-1.708578	-2.453174
40	6	0	-7.617077	-0.974152	-1.985416
41	6	0	4.510687	-2.611190	-0.761508
42	6	0	5.675537	-3.263226	-0.352630
43	6	0	5.817267	-4.627669	-0.585172
44	6	0	4.804176	-5.342884	-1.223266
45	6	0	3.648189	-4.683999	-1.638640
46	6	0	3.498609	-3.317722	-1.414698
47	6	0	6.525630	1.621164	-1.389022
48	6	0	6.545273	3.017135	-1.274553
49	6	0	7.632209	3.748932	-1.743646
50	6	0	8.714690	3.098324	-2.333990
51	6	0	8.701972	1.708564	-2.453172
52	6	0	7.617078	0.974141	-1.985419
53	1	0	0.071319	-2.388177	2.041251
54	1	0	-0.071319	2.388189	2.041237
55	1	0	2.304356	-2.429718	0.739191
56	1	0	2.221387	2.553597	0.760370
57	1	0	-2.304358	2.429723	0.739180
58	1	0	-2.221389	-2.553593	0.760389
59	1	0	0.077838	-2.487855	4.647203
60	1	0	-0.077833	2.487883	4.647188
61	1	0	-2.612535	2.799017	-1.761710
62	1	0	-2.863669	5.229535	-2.152133
63	1	0	-4.918439	6.406525	-1.402746
64	1	0	-6.721550	5.133413	-0.263750
65	1	0	-6.457820	2.695732	0.136047
66	1	0	-5.699844	-3.513900	-0.815895
67	1	0	-7.633988	-4.830079	-1.647590
68	1	0	-9.561888	-3.669618	-2.700054
69	1	0	-9.539637	-1.194910	-2.914177
70	1	0	-7.603984	0.104143	-2.081891
71	1	0	6.457824	-2.695727	0.136044
72	1	0	6.721558	-5.133409	-0.263740
73	1	0	4.918443	-6.406533	-1.402716
74	1	0	2.863665	-5.229552	-2.152097
75	1	0	2.612528	-2.799033	-1.761689
76	1	0	5.699842	3.513895	-0.815914
77	1	0	7.633993	4.830068	-1.647600
78	1	0	9.561898	3.669603	-2.700051
79	1	0	9.539643	1.194894	-2.914169
80	1	0	7.603984	-0.104154	-2.081893
81	1	0	-0.038870	1.236985	6.793302
82	1	0	0.038878	-1.236944	6.793309

Table S21. Optimized geometry of the lowest singlet state of **1** (UB3LYP/6-311G*-IEFPCM(acetone)).

Center Number	Atomic Number	Atomic Type	Coordinates (Angstroms) X	Y	Z
1	6	0	0.041161	-1.300204	2.064307
2	6	0	-1.190994	-0.744161	1.353786
3	6	0	-1.232891	0.667033	1.350297
4	6	0	-0.041161	1.300202	2.064308
5	6	0	0.023111	-0.701401	3.470443
6	6	0	-0.023112	0.701398	3.470444
7	6	0	1.190994	0.744160	1.353787
8	6	0	1.232891	-0.667034	1.350297
9	6	0	2.270325	-1.346369	0.738986
10	6	0	3.289692	-0.610746	0.110206
11	6	0	3.282285	0.812715	0.140061
12	6	0	2.201287	1.472699	0.763455
13	6	0	-2.270326	1.346369	0.738988
14	6	0	-3.289693	0.610746	0.110207
15	6	0	-3.282286	-0.812715	0.140060
16	6	0	-2.201287	-1.472700	0.763453
17	6	0	0.046635	-1.403397	4.668236
18	6	0	0.023185	-0.696304	5.875751
19	6	0	-0.023186	0.696298	5.875752
20	6	0	-0.046636	1.403393	4.668238
21	7	0	4.369160	-1.197854	-0.545261
22	7	0	5.427448	-0.469659	-1.000866
23	6	0	5.331652	0.855302	-0.900522
24	7	0	4.304473	1.546561	-0.395993
25	7	0	-4.369161	1.197854	-0.545260
26	7	0	-5.427449	0.469660	-1.000863
27	6	0	-5.331653	-0.855301	-0.900522
28	7	0	-4.304473	-1.546560	-0.395994
29	6	0	-4.496916	2.611130	-0.747090
30	6	0	-3.499781	3.317488	-1.422352
31	6	0	-3.651477	4.686067	-1.633209
32	6	0	-4.794459	5.345040	-1.182063
33	6	0	-5.791995	4.629461	-0.519415
34	6	0	-5.647549	3.262626	-0.299386
35	6	0	-6.496559	-1.624489	-1.423187
36	6	0	-6.516661	-3.021513	-1.314507
37	6	0	-7.598371	-3.753648	-1.796658
38	6	0	-8.676589	-3.102263	-2.394890
39	6	0	-8.664573	-1.711464	-2.508075
40	6	0	-7.584610	-0.977359	-2.027141
41	6	0	4.496917	-2.611129	-0.747092
42	6	0	5.647548	-3.262625	-0.299386
43	6	0	5.791995	-4.629461	-0.519415
44	6	0	4.794460	-5.345039	-1.182066
45	6	0	3.651480	-4.686065	-1.633215
46	6	0	3.499783	-3.317486	-1.422357
47	6	0	6.496558	1.624490	-1.423186
48	6	0	6.516658	3.021515	-1.314510
49	6	0	7.598369	3.753650	-1.796660
50	6	0	8.676590	3.102265	-2.394887
51	6	0	8.664576	1.711465	-2.508068
52	6	0	7.584612	0.977360	-2.027135
53	1	0	0.074690	-2.389069	2.064490
54	1	0	-0.074691	2.389067	2.064492
55	1	0	2.297711	-2.428160	0.749644
56	1	0	2.205324	2.557291	0.770762
57	1	0	-2.297712	2.428159	0.749647
58	1	0	-2.205324	-2.557292	0.770759
59	1	0	0.082424	-2.488556	4.669611
60	1	0	-0.082424	2.488552	4.669614
61	1	0	-2.622898	2.799392	-1.792707
62	1	0	-2.878676	5.233370	-2.161825
63	1	0	-4.909985	6.410151	-1.350715
64	1	0	-6.684302	5.136213	-0.168207
65	1	0	-6.416354	2.696713	0.212394
66	1	0	-5.677163	-3.522092	-0.849300
67	1	0	-7.599282	-4.834895	-1.704303
68	1	0	-9.519467	-3.673095	-2.770742
69	1	0	-9.498748	-1.196838	-2.973810
70	1	0	-7.575752	0.101365	-2.118839
71	1	0	6.416351	-2.696712	0.212398
72	1	0	6.684301	-5.136213	-0.168205
73	1	0	4.909987	-6.410149	-1.350719
74	1	0	2.878680	-5.233369	-2.161834
75	1	0	2.622902	-2.799391	-1.792715
76	1	0	5.677158	3.522093	-0.849307
77	1	0	7.599278	4.834897	-1.704308
78	1	0	9.519468	3.673097	-2.770739
79	1	0	9.498753	1.196839	-2.973799
80	1	0	7.575756	-0.101364	-2.118830
81	1	0	-0.041204	1.237265	6.816188
82	1	0	0.041203	-1.237272	6.816186

Table S22. Optimized geometry of the lowest triplet state of **1** (UB3LYP/6-311G*-IEFPCM(acetone)).

Center Number	Atomic Number	Atomic Type	Coordinates (Angstroms) X	Y	Z
1	6	0	0.041933	-1.300003	2.027599
2	6	0	-1.193888	-0.744482	1.322296
3	6	0	-1.236987	0.666207	1.319139
4	6	0	-0.041934	1.300007	2.027597
5	6	0	0.023331	-0.701485	3.434796
6	6	0	-0.023332	0.701490	3.434795
7	6	0	1.193887	0.744484	1.322295
8	6	0	1.236986	-0.666205	1.319141
9	6	0	2.281288	-1.344667	0.719177
10	6	0	3.307368	-0.608004	0.102215
11	6	0	3.298330	0.814116	0.131910
12	6	0	2.209973	1.473656	0.743150
13	6	0	-2.281289	1.344668	0.719175
14	6	0	-3.307369	0.608004	0.102214
15	6	0	-3.298331	-0.814116	0.131910
16	6	0	-2.209974	-1.473655	0.743151
17	6	0	0.046992	-1.403420	4.632300
18	6	0	0.023353	-0.696171	5.840066
19	6	0	-0.023355	0.696179	5.840065
20	6	0	-0.046994	1.403427	4.632298
21	7	0	4.395267	-1.195288	-0.541951
22	7	0	5.455178	-0.465349	-0.989315
23	6	0	5.356629	0.860380	-0.890472
24	7	0	4.325426	1.550336	-0.394459
25	7	0	-4.395268	1.195287	-0.541953
26	7	0	-5.455178	0.465348	-0.989316
27	6	0	-5.356629	-0.860381	-0.890473
28	7	0	-4.325426	-1.550337	-0.394459
29	6	0	-4.528075	2.608515	-0.739693
30	6	0	-3.539038	3.318906	-1.422701
31	6	0	-3.696122	4.687553	-1.629204
32	6	0	-4.836558	5.342692	-1.166211
33	6	0	-5.826186	4.623152	-0.496019
34	6	0	-5.676228	3.256266	-0.280168
35	6	0	-6.524798	-1.630163	-1.405016
36	6	0	-6.542789	-3.027319	-1.297402
37	6	0	-7.627391	-3.760036	-1.772015
38	6	0	-8.710700	-3.109128	-2.361584
39	6	0	-8.700825	-1.718260	-2.473673
40	6	0	-7.617940	-0.983535	-2.000217
41	6	0	4.528075	-2.608516	-0.739690
42	6	0	5.676228	-3.256266	-0.280162
43	6	0	5.826187	-4.623152	-0.496013
44	6	0	4.836560	-5.342692	-1.166207
45	6	0	3.696125	-4.687554	-1.629203
46	6	0	3.539040	-3.318907	-1.422700
47	6	0	6.524798	1.630161	-1.405017
48	6	0	6.542789	3.027318	-1.297408
49	6	0	7.627391	3.760032	-1.772022
50	6	0	8.710701	3.109123	-2.361587
51	6	0	8.700827	1.718254	-2.473670
52	6	0	7.617941	0.983531	-2.000213
53	1	0	0.075835	-2.388859	2.027817
54	1	0	-0.075836	2.388862	2.027814
55	1	0	2.309070	-2.426450	0.729666
56	1	0	2.212956	2.558283	0.749647
57	1	0	-2.309071	2.426451	0.729662
58	1	0	-2.212956	-2.558282	0.749649
59	1	0	0.083117	-2.488568	4.633718
60	1	0	-0.083119	2.488575	4.633715
61	1	0	-2.664415	2.803892	-1.802557
62	1	0	-2.929607	5.237911	-2.163773
63	1	0	-4.956308	6.407857	-1.331538
64	1	0	-6.716535	5.126869	-0.135590
65	1	0	-6.438860	2.687364	0.237512
66	1	0	-5.699356	-3.527547	-0.838978
67	1	0	-7.626615	-4.841359	-1.680581
68	1	0	-9.555844	-3.680438	-2.731573
69	1	0	-9.538942	-1.204010	-2.932694
70	1	0	-7.610786	0.095260	-2.091081
71	1	0	6.438858	-2.687364	0.237519
72	1	0	6.716535	-5.126869	-0.135581
73	1	0	4.956311	-6.407858	-1.331533
74	1	0	2.929611	-5.237913	-2.163773
75	1	0	2.664418	-2.803894	-1.802559
76	1	0	5.699355	3.527547	-0.838988
77	1	0	7.626614	4.841356	-1.680594
78	1	0	9.555846	3.680431	-2.731578
79	1	0	9.538945	1.204002	-2.932688
80	1	0	7.610788	-0.095264	-2.091073
81	1	0	-0.041543	1.237236	6.780449
82	1	0	0.041541	-1.237227	6.780451

Table S23. Optimized geometry of the lowest singlet state of **1** (UB3LYP/6-311G*-IEFPCM(DMSO)).

Center Number	Atomic Number	Atomic Type	Coordinates (Angstroms) X	Y	Z
1	6	0	0.041381	-1.300373	2.061584
2	6	0	-1.190871	-0.744350	1.351236
3	6	0	-1.233077	0.666878	1.347813
4	6	0	-0.041381	1.300369	2.061587
5	6	0	0.023205	-0.701428	3.467724
6	6	0	-0.023206	0.701423	3.467725
7	6	0	1.190871	0.744348	1.351237
8	6	0	1.233076	-0.666880	1.347812
9	6	0	2.271031	-1.346121	0.737220
10	6	0	3.290695	-0.610227	0.109241
11	6	0	3.282785	0.813238	0.138723
12	6	0	2.201312	1.473136	0.761302
13	6	0	-2.271032	1.346120	0.737223
14	6	0	-3.290695	0.610227	0.109242
15	6	0	-3.282785	-0.813238	0.138722
16	6	0	-2.201312	-1.473138	0.761299
17	6	0	0.046790	-1.403502	4.665561
18	6	0	0.023266	-0.696342	5.873116
19	6	0	-0.023266	0.696332	5.873117
20	6	0	-0.046791	1.403494	4.665564
21	7	0	4.370803	-1.197268	-0.545082
22	7	0	5.428729	-0.468910	-1.001244
23	6	0	5.332656	0.856068	-0.901258
24	7	0	4.305262	1.547221	-0.397049
25	7	0	-4.370804	1.197269	-0.545080
26	7	0	-5.428730	0.468912	-1.001242
27	6	0	-5.332657	-0.856067	-0.901258
28	7	0	-4.305262	-1.547220	-0.397053
29	6	0	-4.499607	2.610882	-0.744986
30	6	0	-3.505217	3.317865	-1.423583
31	6	0	-3.657992	4.686663	-1.632546
32	6	0	-4.799297	5.345013	-1.176159
33	6	0	-5.794033	4.628771	-0.509933
34	6	0	-5.648438	3.261715	-0.291703
35	6	0	-6.497607	-1.625459	-1.423821
36	6	0	-6.517985	-3.022498	-1.314489
37	6	0	-7.599731	-3.754831	-1.796407
38	6	0	-8.677865	-3.103638	-2.395107
39	6	0	-8.665710	-1.712842	-2.508908
40	6	0	-7.585681	-0.978598	-2.028176
41	6	0	4.499608	-2.610881	-0.744989
42	6	0	5.648437	-3.261714	-0.291704
43	6	0	5.794033	-4.628770	-0.509936
44	6	0	4.799299	-5.345011	-1.176165
45	6	0	3.657995	-4.686660	-1.632555
46	6	0	3.505220	-3.317863	-1.423590
47	6	0	6.497607	1.625461	-1.423820
48	6	0	6.517982	3.022501	-1.314490
49	6	0	7.599729	3.754834	-1.796408
50	6	0	8.677865	3.103641	-2.395104
51	6	0	8.665712	1.712845	-2.508901
52	6	0	7.585683	0.978601	-2.028170
53	1	0	0.075112	-2.389161	2.061718
54	1	0	-0.075112	2.389157	2.061722
55	1	0	2.298452	-2.427893	0.747802
56	1	0	2.204667	2.557729	0.768525
57	1	0	-2.298452	2.427892	0.747807
58	1	0	-2.204666	-2.557730	0.768519
59	1	0	0.082762	-2.488622	4.666921
60	1	0	-0.082762	2.488614	4.666926
61	1	0	-2.629524	2.800358	-1.797539
62	1	0	-2.887288	5.234642	-2.163465
63	1	0	-4.915591	6.410270	-1.343240
64	1	0	-6.684807	5.135139	-0.154375
65	1	0	-6.414858	2.695507	0.223362
66	1	0	-5.678851	-3.523287	-0.848864
67	1	0	-7.600735	-4.836001	-1.703455
68	1	0	-9.520745	-3.674557	-2.770734
69	1	0	-9.499806	-1.198343	-2.974866
70	1	0	-7.577131	0.100087	-2.120339
71	1	0	6.414855	-2.695507	0.223364
72	1	0	6.684806	-5.135138	-0.154375
73	1	0	4.915593	-6.410268	-1.343247
74	1	0	2.887293	-5.234639	-2.163477
75	1	0	2.629527	-2.800355	-1.797548
76	1	0	5.678847	3.523290	-0.848868
77	1	0	7.600731	4.836004	-1.703458
78	1	0	9.520745	3.674561	-2.770729
79	1	0	9.499810	1.198346	-2.974856
80	1	0	7.577134	-0.100085	-2.120331
81	1	0	-0.041357	1.237306	6.813539
82	1	0	0.041356	-1.237318	6.813537

Table S24. Optimized geometry of the lowest triplet state of **1** (UB3LYP/6-311G*-IEFPCM(DMSO)).

Center Number	Atomic Number	Atomic Type	Coordinates (Angstroms) X	Y	Z
1	6	0	0.042155	-1.300175	2.024526
2	6	0	-1.193784	-0.744680	1.319432
3	6	0	-1.237182	0.666042	1.316334
4	6	0	-0.042156	1.300175	2.024527
5	6	0	0.023433	-0.701514	3.431732
6	6	0	-0.023434	0.701514	3.431732
7	6	0	1.193784	0.744680	1.319433
8	6	0	1.237182	-0.666042	1.316334
9	6	0	2.282037	-1.344414	0.717169
10	6	0	3.308456	-0.607481	0.101092
11	6	0	3.298925	0.814644	0.130423
12	6	0	2.210057	1.474099	0.740764
13	6	0	-2.282037	1.344414	0.717169
14	6	0	-3.308456	0.607481	0.101092
15	6	0	-3.298925	-0.814644	0.130422
16	6	0	-2.210058	-1.474099	0.740763
17	6	0	0.047164	-1.403528	4.629279
18	6	0	0.023442	-0.696211	5.837087
19	6	0	-0.023443	0.696210	5.837087
20	6	0	-0.047165	1.403526	4.629279
21	7	0	4.397026	-1.194700	-0.541844
22	7	0	5.456617	-0.464608	-0.989692
23	6	0	5.357800	0.861138	-0.891192
24	7	0	4.326360	1.550993	-0.395567
25	7	0	-4.397026	1.194701	-0.541844
26	7	0	-5.456617	0.464609	-0.989691
27	6	0	-5.357800	-0.861137	-0.891193
28	7	0	-4.326359	-1.550993	-0.395569
29	6	0	-4.530926	2.608272	-0.737536
30	6	0	-3.544756	3.319379	-1.423886
31	6	0	-3.702972	4.688250	-1.628354
32	6	0	-4.841660	5.342679	-1.159971
33	6	0	-5.828361	4.622387	-0.486188
34	6	0	-5.677201	3.255273	-0.272281
35	6	0	-6.526067	-1.631117	-1.405515
36	6	0	-6.544565	-3.028247	-1.296743
37	6	0	-7.629257	-3.761161	-1.770993
38	6	0	-8.712298	-3.110486	-2.361419
39	6	0	-8.702049	-1.719662	-2.474638
40	6	0	-7.619049	-0.984798	-2.001501
41	6	0	4.530926	-2.608272	-0.737536
42	6	0	5.677201	-3.255273	-0.272280
43	6	0	5.828362	-4.622387	-0.486188
44	6	0	4.841662	-5.342679	-1.159972
45	6	0	3.702973	-4.688250	-1.628356
46	6	0	3.544757	-3.319378	-1.423888
47	6	0	6.526067	1.631117	-1.405515
48	6	0	6.544564	3.028248	-1.296745
49	6	0	7.629256	3.761161	-1.770996
50	6	0	8.712299	3.110485	-2.361419
51	6	0	8.702050	1.719662	-2.474635
52	6	0	7.619050	0.984798	-2.001498
53	1	0	0.076275	-2.388953	2.024685
54	1	0	-0.076276	2.388952	2.024686
55	1	0	2.309844	-2.426177	0.727586
56	1	0	2.212370	2.558725	0.747184
57	1	0	-2.309845	2.426177	0.727587
58	1	0	-2.212370	-2.558726	0.747182
59	1	0	0.083483	-2.488636	4.630680
60	1	0	-0.083484	2.488635	4.630681
61	1	0	-2.671366	2.805030	-1.807446
62	1	0	-2.938647	5.239357	-2.165229
63	1	0	-4.962216	6.407994	-1.323619
64	1	0	-6.717117	5.125653	-0.121291
65	1	0	-6.437346	2.686005	0.248687
66	1	0	-5.701632	-3.528655	-0.837609
67	1	0	-7.628760	-4.842374	-1.678562
68	1	0	-9.557485	-3.681882	-2.731090
69	1	0	-9.539939	-1.205574	-2.934195
70	1	0	-7.612019	0.093923	-2.093222
71	1	0	6.437345	-2.686005	0.248689
72	1	0	6.717117	-5.125653	-0.121289
73	1	0	4.962218	-6.407994	-1.323620
74	1	0	2.938649	-5.239357	-2.165233
75	1	0	2.671368	-2.805029	-1.807449
76	1	0	5.701630	3.528656	-0.837613
77	1	0	7.628758	4.842374	-1.678566
78	1	0	9.557485	3.681882	-2.731090
79	1	0	9.539941	1.205573	-2.934189
80	1	0	7.612021	-0.093923	-2.093217
81	1	0	-0.041709	1.237274	6.777458
82	1	0	0.041708	-1.237276	6.777457

Table S25. Calculated excitations of **1** at the lowest singlet state of **1** (UB3LYP/6-311G*-IEFPCM(toluene)).

```
Excited State   1:  1.234-A     1.5463 eV  801.83 nm  f=0.0003  <S**2>=0.131
    174A -> 175A       0.70448
    174B -> 175B       0.70449
 This state for optimization and/or second-order correction.
 Total Energy, E(TD-HF/TD-DFT) =  -2099.80533457
 Copying the excited state density for this state as the 1-particle RhoCI density.

 Excited State   2:  1.149-A     1.6177 eV  766.43 nm  f=0.1397  <S**2>=0.080
    174A -> 175A       0.70445
    174B -> 175B      -0.70444

 Excited State   3:  2.314-A     2.0289 eV  611.08 nm  f=0.0003  <S**2>=1.089
    173A -> 175A       0.10701
    174A -> 176A       0.65767
    174A -> 177A      -0.18825
    173B -> 175B       0.10703
    174B -> 176B       0.65757
    174B -> 177B      -0.18822

 Excited State   4:  2.265-A     2.0396 eV  607.90 nm  f=0.0003  <S**2>=1.032
    173A -> 175A       0.12204
    174A -> 176A      -0.64764
    174A -> 177A       0.20928
    173B -> 175B      -0.12202
    174B -> 176B       0.64774
    174B -> 177B      -0.20931

 Excited State   5:  2.367-A     2.6543 eV  467.11 nm  f=0.0045  <S**2>=1.151
    170A -> 175A       0.10773
    173A -> 175A       0.61008
    174A -> 176A      -0.11272
    174A -> 177A      -0.10747
    174A -> 178A       0.17276
    170B -> 175B       0.10778
    173B -> 175B       0.61035
    174B -> 176B      -0.11267
    174B -> 177B      -0.10745
    174B -> 178B       0.17268
```

Table S26. Calculated excitations of **1** at the lowest triplet state of **1** (UB3LYP/6-311G*-IEFPCM(toluene)).

```
Excited State   1:  3.043-A     1.9749 eV  627.80 nm  f=0.0002  <S**2>=2.065
    174A -> 177A      -0.47346
    175A -> 176A       0.84705
    172B -> 174B       0.10245
 This state for optimization and/or second-order correction.
 Total Energy, E(TD-HF/TD-DFT) =  -2099.78881734
 Copying the excited state density for this state as the 1-particle RhoCI density.

 Excited State   2:  3.039-A     2.0357 eV  609.06 nm  f=0.0027  <S**2>=2.059
    174A -> 176A      -0.61317
    175A -> 177A       0.74122
    172B -> 175B       0.10369
    173B -> 174B       0.15326

 Excited State   3:  3.083-A     2.6039 eV  476.15 nm  f=0.0042  <S**2>=2.126
    174A -> 177A      -0.12241
```

```
   174A -> 178A     -0.15142
   175A -> 176A     -0.18984
   175A -> 179A      0.19280
   170B -> 174B     -0.13660
   171B -> 175B     -0.13630
   172B -> 174B      0.77040
   173B -> 175B      0.42038

 Excited State   4:  3.083-A      2.6306 eV  471.31 nm  f=0.0323  <S**2>=2.126
   174A -> 176A      0.17523
   174A -> 179A     -0.16842
   175A -> 178A      0.31928
   167B -> 174B     -0.10589
   171B -> 174B     -0.15614
   172B -> 175B      0.47276
   173B -> 174B      0.71052

 Excited State   5:  3.048-A      2.7586 eV  449.44 nm  f=0.1051  <S**2>=2.073
   174A -> 176A      0.58852
   174A -> 179A     -0.19502
   175A -> 177A      0.58667
   175A -> 178A      0.40874
   175A -> 182A     -0.10076
   172B -> 175B     -0.13815
   173B -> 174B     -0.21093
```

Table S27. Calculated excitations of **1** at the lowest singlet state of **1** (UB3LYP/6-311G*-IEFPCM(acetone)).

```
Excited State   1:  1.232-A     1.5487 eV  800.57 nm  f=0.0003  <S**2>=0.129
   174A -> 175A      0.70439
   174B -> 175B      0.70445
 This state for optimization and/or second-order correction.
 Total Energy, E(TD-HF/TD-DFT) =  -2099.81578529
 Copying the excited state density for this state as the 1-particle RhoCI density.

 Excited State   2:  1.140-A      1.6253 eV  762.82 nm  f=0.1355  <S**2>=0.075
   174A -> 175A      0.70471
   174B -> 175B     -0.70466

 Excited State   3:  2.312-A      2.0076 eV  617.58 nm  f=0.0002  <S**2>=1.086
   174A -> 176A      0.66120
   174A -> 177A     -0.17866
   174B -> 176B      0.66143
   174B -> 177B     -0.17873

 Excited State   4:  2.264-A      2.0176 eV  614.50 nm  f=0.0005  <S**2>=1.032
   173A -> 175A     -0.11351
   174A -> 176A      0.65181
   174A -> 177A     -0.20212
   173B -> 175B      0.11354
   174B -> 176B     -0.65158
   174B -> 177B      0.20206

 Excited State   5:  2.373-A      2.6723 eV  463.96 nm  f=0.0051  <S**2>=1.158
   170A -> 175A      0.10830
   172A -> 175A      0.10326
   173A -> 175A      0.61379
   174A -> 176A     -0.11313
   174A -> 177A     -0.13582
```

```
   174A -> 178A      0.14119
   170B -> 175B      0.10829
   172B -> 175B      0.10326
   173B -> 175B      0.61373
   174B -> 176B     -0.11314
   174B -> 177B     -0.13583
   174B -> 178B      0.14121
```

Table S28. Calculated excitations of **1** at the lowest triplet state of **1** (UB3LYP/6-311G*-IEFPCM(acetone)).

```
Excited State   1:  3.041-A     1.9547 eV  634.27 nm  f=0.0002  <S**2>=2.062
   174A -> 177A      0.47809
   175A -> 176A      0.84590
 This state for optimization and/or second-order correction.
 Total Energy, E(TD-HF/TD-DFT) =  -2099.80009255
 Copying the excited state density for this state as the 1-particle RhoCI density.

 Excited State   2:  3.037-A     2.0127 eV  616.02 nm  f=0.0025  <S**2>=2.056
   174A -> 176A      0.61063
   175A -> 177A      0.74691
   173B -> 174B      0.14206

 Excited State   3:  3.085-A     2.6216 eV  472.93 nm  f=0.0045  <S**2>=2.130
   174A -> 177A     -0.15730
   174A -> 178A     -0.12558
   175A -> 176A      0.20280
   175A -> 179A     -0.16419
   175A -> 186A      0.10788
   170B -> 174B      0.12065
   171B -> 175B      0.15818
   172B -> 174B      0.77650
   173B -> 175B     -0.41543

 Excited State   4:  3.087-A     2.6510 eV  467.69 nm  f=0.0473  <S**2>=2.133
   174A -> 176A     -0.18975
   174A -> 179A      0.13775
   175A -> 178A      0.24406
   169B -> 174B     -0.10671
   171B -> 174B     -0.19323
   172B -> 175B     -0.48540
   173B -> 174B      0.72877

 Excited State   5:  3.038-A     2.7442 eV  451.80 nm  f=0.0223  <S**2>=2.057
   174A -> 176A      0.72508
   175A -> 177A     -0.64551
   173B -> 174B      0.14405
```

Table S29. Calculated excitations of **1** at the lowest singlet state of **1** (UB3LYP/6-311G*-IEFPCM(DMSO)).

```
Excited State   1:  1.231-A     1.5491 eV  800.39 nm  f=0.0003  <S**2>=0.129
   174A -> 175A      0.70441
   174B -> 175B      0.70441
 This state for optimization and/or second-order correction.
 Total Energy, E(TD-HF/TD-DFT) =  -2099.81687588
 Copying the excited state density for this state as the 1-particle RhoCI density.

 Excited State   2:  1.142-A     1.6241 eV  763.42 nm  f=0.1378  <S**2>=0.076
   174A -> 175A      0.70471
   174B -> 175B     -0.70470
```

```
 Excited State   3:  2.312-A      2.0056 eV  618.20 nm  f=0.0002  <S**2>=1.086
    174A -> 176A       0.66160
    174A -> 177A      -0.17765
    174B -> 176B       0.66171
    174B -> 177B      -0.17768

 Excited State   4:  2.264-A      2.0155 eV  615.16 nm  f=0.0006  <S**2>=1.032
    173A -> 175A      -0.11290
    174A -> 176A       0.65218
    174A -> 177A      -0.20122
    173B -> 175B       0.11292
    174B -> 176B      -0.65208
    174B -> 177B       0.20119

 Excited State   5:  2.374-A      2.6726 eV  463.92 nm  f=0.0054  <S**2>=1.159
    170A -> 175A      -0.10680
    172A -> 175A       0.10571
    173A -> 175A       0.61473
    174A -> 176A      -0.11320
    174A -> 177A      -0.13707
    174A -> 178A       0.13589
    170B -> 175B      -0.10680
    172B -> 175B       0.10572
    173B -> 175B       0.61474
    174B -> 176B      -0.11320
    174B -> 177B      -0.13707
    174B -> 178B       0.13589
```

Table S30. Calculated excitations of **1** at the lowest triplet state of **1** (UB3LYP/6-311G*-IEFPCM(DMSO)).

```
Excited State   1:  3.041-A      1.9530 eV  634.85 nm  f=0.0002  <S**2>=2.062
    174A -> 177A       0.47875
    175A -> 176A       0.84562
 This state for optimization and/or second-order correction.
 Total Energy, E(TD-HF/TD-DFT) =  -2099.80126209
 Copying the excited state density for this state as the 1-particle RhoCI density.

 Excited State   2:  3.037-A      2.0104 eV  616.71 nm  f=0.0025  <S**2>=2.056
    174A -> 176A       0.61034
    175A -> 177A       0.74741
    173B -> 174B      -0.14123

 Excited State   3:  3.085-A      2.6219 eV  472.87 nm  f=0.0048  <S**2>=2.130
    174A -> 177A      -0.15822
    174A -> 178A      -0.12118
    175A -> 176A       0.20355
    175A -> 179A      -0.15888
    175A -> 186A      -0.10824
    170B -> 174B       0.11789
    171B -> 175B      -0.16087
    172B -> 174B       0.77779
    173B -> 175B       0.41563

 Excited State   4:  3.087-A      2.6515 eV  467.60 nm  f=0.0512  <S**2>=2.133
    174A -> 176A       0.18899
    174A -> 179A      -0.13253
    175A -> 178A      -0.23255
    169B -> 174B       0.10626
    171B -> 174B      -0.19793
```

```
   172B -> 175B       0.48763
   173B -> 174B       0.73113

Excited State   5:  3.038-A      2.7424 eV  452.10 nm  f=0.0218  <S**2>=2.057
   174A -> 176A       0.72721
   175A -> 177A      -0.64515
   173B -> 174B      -0.14072
```

11. Supporting References

31. Stoll, S.; Schweiger, A. EasySpin, a comprehensive soft-ware package for spectral simulation and analysis in EPR. *J. Magn. Reson.* **2006**, *178*, 42–55.
32. Wojdyr, M. Fityk: a general-purpose peak fitting program. *J. Appl. Cryst.* **2010**, *43*, 1126–1128.
33. Sheldrick, G. M. SHELXT - Integrated space-group and crystal-structure determination. *Acta Cryst.* **2015**, *A71*, 3–8.
34. Sheldrick, G. M. Crystal structure refinement with SHELXL. *Acta Cryst.* **2015**, *C71*, 3–8.
35. Gaussian 16, Revision C.01, Frisch, M. J.; Trucks, G. W.; Schlegel, H. B.; Scuseria, G. E.; Robb, M. A.; Cheeseman, J. R.; Scalmani, G.; Barone, V.; Petersson, G. A.; Nakatsuji, H.; Li, X.; Caricato, M.; Marenich, A. V.; Bloino, J.; Janesko, B. G.; Gomperts, R.; Mennucci, B.; Hratchian, H. P.; Ortiz, J. V.; Izmaylov, A. F.; Sonnenberg, J. L.; Williams-Young, D.; Ding, F.; Lipparini, F.; Egidi, F.; Goings, J.; Peng, B.; Petrone, A.; Henderson, T.; Ranasinghe, D.; Zakrzewski, V. G.; Gao, J.; Rega, N.; Zheng, G.; Liang, W.; Hada, M.; Ehara, M.; Toyota, K.; Fukuda, R.; Hasegawa, J.; Ishida, M.; Nakajima, T.; Honda, Y.; Kitao, O.; Nakai, H.; Vreven, T.; Throssell, K.; Montgomery, J. A., Jr.; Peralta, J. E.; Ogliaro, F.; Bearpark, M. J.; Heyd, J. J.; Brothers, E. N.; Kudin, K. N.; Staroverov, V. N.; Keith, T. A.; Kobayashi, R.; Normand, J.; Raghavachari, K.; Rendell, A. P.; Burant, J. C.; Iyengar, S. S.; Tomasi, J.; Cossi, M.; Millam, J. M.; Klene, M.; Adamo, C.; Cammi, R.; Ochterski, J. W.; Martin, R. L.; Morokuma, K.; Farkas, O.; Foresman, J. B.; Fox, D. J. Gaussian, Inc., Wallingford CT, 2016.
36. Lu, T.; Chen, F. Multiwfn: A multifunctional wavefunction analyzer. *J. Comput. Chem.* **2012**, *33*, 580–592.
37. Epifanovsky, E.; Gilbert, A. T. B.; Feng, X.; Lee, J.; Mao, Y.; Mardirossian, N.; Pokhilko, P.; White, A. F.; Coons, M. P.; Dempwolff, A. L.; Gan, Z.; Hait, D.; Horn, P. R.; Jacobson, L. D.; Kaliman, I.; Kussmann, J.; Lange, A. W.; Lao, K. U.; Levine, D. S.; Liu, J.; McKenzie, S. C.; Morrison, A. F.; Nanda, K. D.; Plasser, F.; Rehn, D. R.; Vidal, M. L.; You, Z.-Q.; Zhu, Y.; Alam, B.; Albrecht, B. J.; Aldossary, A.; Alguire, E.; Andersen, J. H.; Athavale, V.; Barton, D.; Begam, K.; Behn, A.; Bellonzi, N.; Bernard, Y. A.; Berquist, E. J.; Burton, H. G. A.; Carreras, A.; Carter-Fenk, K.; Chakraborty, R.; Chien, A. D.; Closser, K. D.; Cofer-Shabica, V.; Dasgupta, S.; de Wergifosse, M.; Deng, J.; Diedenhofen, M.; Do, H.; Ehlert, S.; Fang, P.-T.; Fatehi, S.; Feng, Q.; Friedhoff, T.; Gayvert, J.; Ge, Q.; Gidofalvi, G.; Goldey, M.; Gomes, J.; González-Espinoza, C. E.; Gulania, S.; Gunina, A. O.; Hanson-Heine, M. W. D.; Harbach, P. H. P.; Hauser, A.; Herbst, M. F.; Hernández Vera, M.; Hodecker, M.; Holden, Z. C.; Houck, S.; Huang, X.; Hui, K.; Huynh, B. C.; Ivanov, M.; Jász, Á.; Ji, H.; Jiang, H.; Kaduk, B.; Kähler, S.; Khistyaev, K.; Kim, J.; Kis, G.; Klunzinger, P.; Koczor-Benda, Z.; Koh, J. H.; Kosenkov, D.; Koulias, L.; Kowalczyk, T.; Krauter, C. M.; Kue, K.; Kunitsa, A.; Kus, T.; Ladjánszki, I.; Landau, A.; Lawler, K. V; Lefrancois, D.; Lehtola, S.; Li, R. R.; Li, Y.-P.; Liang, J.; Liebenthal, M.; Lin, H.-H.; Lin, Y.-S.; Liu, F.; Liu, K.-Y.; Loipersberger, M.; Luenser, A.; Manjanath, A.; Manohar, P.; Mansoor, E.; Manzer, S. F.; Mao, S.-P.; Marenich, A. V; Markovich, T.; Mason, S.; Maurer, S. A.; McLaughlin, P. F.; Menger, M. F. S. J.; Mewes, J.-M.; Mewes, S. A.; Morgante, P.; Mullinax, J. W.; Oosterbaan, K. J.; Paran, G.; Paul, A. C.; Paul, S. K.; Pavošević, F.; Pei, Z.; Prager, S.; Proynov, E. I.; Rák, Á.; Ramos-Cordoba, E.; Rana, B.; Rask, A. E.; Rettig, A.; Richard, R. M.; Rob, F.; Rossomme, E.; Scheele, T.; Scheurer, M.; Schneider, M.; Sergueev, N.; Sharada, S. M.; Skomorowski, W.; Small, D. W.; Stein, C. J.; Su, Y.-C.; Sundstrom, E. J.; Tao, Z.; Thirman, J.; Tornai, G. J.; Tsuchimochi, T.; Tubman, N. M.; Veccham, S. P.; Vydrov, O.; Wenzel, J.; Witte, J.; Yamada, A.; Yao, K.; Yeganeh, S.; Yost, S. R.; Zech, A.; Zhang, I. Y.; Zhang, X.; Zhang, Y.; Zuev, D.; Aspuru-Guzik, A.; Bell, A. T.; Besley, N. A.; Bravaya, K. B.; Brooks, B. R.; Casanova, D.; Chai, J.-D.; Coriani, S.; Cramer, C. J.; Cserey, G.; DePrince III, A. E.; DiStasio Jr., R. A.; Dreuw, A.; Dunietz, B. D.; Furlani, T. R.; Goddard III, W. A.; Hammes-Schiffer, S.; Head-Gordon, T.; Hehre, W. J.; Hsu, C.-P.; Jagau, T.-C.; Jung, Y.; Klamt, A.; Kong, J.; Lambrecht, D. S.; Liang, W.; Mayhall, N. J.; McCurdy, C. W.; Neaton, J. B.; Ochsenfeld, C.; Parkhill, J. A.; Peverati, R.; Rassolov, V. A.; Shao, Y.; Slipchenko, L. V; Stauch, T.; Steele, R. P.; Subotnik, J. E.; Thom, A. J. W.; Tkatchenko, A.; Truhlar, D. G.; Van Voorhis, T.; Wesolowski, T. A.; Whaley, K. B.; Woodcock III, H. L.; Zimmerman, P. M.; Faraji, S.; Gill, P. M. W.; Head-Gordon, M.; Herbert, J. M.; Krylov, A. I. Software for the Frontiers of Quantum Chemistry: An Overview of Developments in the Q-Chem 5 Package. *J. Chem. Phys.* **2021**, *155*, 84801.
