# Supplementary material for: Optically Distinguishable Electronic Spin-isomers of a Stable Organic Diradical
Source: ACS Cent Sci. 2024 Apr 8;10(4):890–8. doi: 10.1021/acscentsci.4c00284 (PMC11046471; doi:10.1021/acscentsci.4c00284)
Supplement: Supplementary file 2 — oc4c00284_si_002.pdf [file oc4c00284_si_002.pdf]

oc-2024-002847.R1

Name: Peer Review Information for "Optically Distinguishable Electronic Spin-isomers of a Stable Organic Diradical"

First Round of Reviewer Comments

Reviewer: 1

Comments to the Author

1. Title and general comments; 'spin-isomers' change to 'singlet state'

Reason: electronic states are not isomers and only singlet state is optically accessible (in NIR) with good selectivity, because the singlet dominates NIR absorption. While the triplet could be in principle accessed at shorter wavelengths, the problem is that both singlet and triplet strongly absorb there and at rt, these two states are approximately in 1:1 ratio. Note that the fits to 400 nm excitation (decays) were highly constrained thus not particularly informative. Also note that "optical accessibility" is not quite practical yet, as half-lives of the excited states are in the ps (and sub-ps) range, thus suggesting very low quantum yields of luminescence (why not measured??). This system is incomparably inferior to S = 1 nitrogen defect states in diamond, as far as potential applications are concerned but it is another incremental step forward. Thus, the revised ms may be considered for publication in Centr. Sci.

2. fig. 3c and analogous fig in the SI; results of the fit does not make sense; I thought singlet absorption (area) should dominate in NIR.

3. page 3 (col1, bottom); "According to the Boltzmann distribution and experimental

DEST (3.0 kJ/mol)..." Put minus sign - it should be –3.0 kJ/mol. Check elsewhere.

4. TD-DFT computations (on the lowest singlet and triplet state) should be carried out in PCM models for solvents with different polarity and to use them to rationalize the experimental results, in particular the degree of CT (if any) in the excited state.

Reviewer: 2

Comments to the Author

This is an interesting study by Shimizu et al. on stable organic diradical. They developed a novel model of electronic spin isomers using this through-space conjugated diradical. A small singlet-triplet energy gap of [illegible]3.0 kJ/mol was determined by SQUID magnetometry and VT-EPR, affording ca. 1[illegible] 1 coexistence of two spin states at room temperature. The singlet-specific absorption band was observed in NIR experiments. Excited-state dynamics of each spin state were investigated by transient absorption spectroscopy indicating the selectively photoexcite one of the two coexisting spin states. Besides, electrochemical studies of diradical 1 were carried out using cyclic voltammetry and differential pulse voltammetry. The experiments were performed by experts and the analysis of the spectra seems to be conclusive, and the results served to sharpen insight into electronic properties of diradicals and could potentially lead to deeper studies into properties of multi-radicals, which also match well with the scope of ACS Central Science. Here are some minor comments:

1. Could the authors provide an explanation on the difference between the electronic spin-isomers mentioned this article and the magnetic bistability which was observed in many diradicals.

2. The free electrons of diradical 1 were located in different N atoms in Figure 2 and Figure 4. Is there a movement of diradical? Or maybe it is a mistake?

3. The last sentence of the last paragraph on page 3, and the line 13 on page 6, please check compound 7 and compound 6?

4. In the last sentence on page 5, ground-state bleaching band around 650 nm in Figure 5h, could authors provide an explanation why these is a ground-state bleaching band for diradical 1. However, there is no bleaching band for monomer 5. How to assign 650 nm?

Author's Response to Peer Review Comments:

Reply to Reviewer #1

Ms.ID: oc-2024-002847

Title: **“Optically distinguishable electronic spin isomers of an organic diradical”**

Authors: Daiki Shimizu, Hiakaru Sotome, Hiroshi Miyasaka, and Kenji Matsuda

Thank you for reviewing our manuscript carefully. According to your comments, we have revised our manuscript as follows:

Comment (1). Title and general comments; ‘spin-isomers’ change to ‘singlet state’

Reason: electronic states are not isomers and only singlet state is optically accessible (in NIR) with good selectivity, because the singlet dominates NIR absorption. While the triplet could be in principle accessed at shorter wavelengths, the problem is that both singlet and triplet strongly

absorb there and at rt, these two states are approximately in 1:1 ratio. Note that the fits to 400 nm excitation (decays) were highly constrained thus not particularly informative. Also note that “optical accessibility” is not quite practical yet, as half-lives of the excited states are in the ps (and sub-ps) range, thus suggesting very low quantum yields of luminescence (why not measured??). This system is incomparably inferior to S = 1 nitrogen defect states in diamond, as far as potential applications are concerned but it is another incremental step forward. Thus, the revised ms may be considered for publication in Centr. Sci.

Answer (1): Thank you for the detailed comments. As the reviewer pointed out, only one of the two co-existing spinstates can be selectively photo-excited in the presented molecular system. We added the following notes in the conclusion to clarify this point.

“The drawback of the present system is that only one of the two spin states can be selectively photoexcited, and the excitation lifetime is relatively short.”

The present system is indeed inferior to defect states in diamonds for potential applications. However, we believe this work is a major step forward, and the drawback does not detract from our idea of spin isomer. We are also currently working on designing molecular systems to realize that both spin states can be optically accessible.

>>Also note that “optical accessibility” is not quite practical yet, as half-lives of the excited states are in the ps (and sub-ps) range, thus suggesting very low quantum yields of luminescence (why not measured??).

We could not detect any emission from compound **1** due to the fast decay of the excited state. Our transient absorption study suggested that the employed radical unit has a short excited-state lifetime and is non-emissive. Since many luminescent radicals have been found in recent years, observing spin-state selective emission is also possible by extending the excited-state lifetime in future work.

Comment (2). fig. 3c and analogous fig in the SI; results of the fit does not make sense; I thought singlet absorption (area) should dominate in NIR.

Answer (2). We are sorry for the confusion. It was our mistake that the “singlet” and “triplet” subscripts in Figure 3c and Figure S18 were reversed. We corrected the Figures.

Comment (3). page 3 (col1, bottom); “According to the Boltzmann distribution and experimental DEST (3.0 kJ/mol)…” Put minus sign - it should be –3.0 kJ/mol. Check elsewhere.

Answer (3). Thank you for pointing this out. We checked throughout the manuscript and SI, and we found the same mistake on page S21 in the SI. We added minus signs at these points.

Comment (4). TD-DFT computations (on the lowest singlet and triplet state) should be carried out in PCM models for solvents with different polarity and to use them to rationalize the experimental results, in particular the degree of CT (if any) in the excited state.

Answer (4). Thank you for the suggestion. We conducted structural optimization and TD-DFT calculation, assuming solvent polarity with the IEFPCM models based on the energy-minimized structure. We selected three solvents (toluene, acetone, DMSO) which were also used for the experimental study (Figure S20). The calculation predicted that the solvent polarity has a negligible effect on the transition energy. Namely, the S_0-S_1 absorption wavelength of singlet **1** was 803 nm for vacuum (f=0.0002) and 800 nm for DMSO (f=0.0003). Similarly, the T_1-T_2 absorption wavelength of triplet **1** was 619 nm for vacuum (f=0.0002) and 635 nm for DMSO (f=0.0002). These results are consistent with the experimental behavior and our conclusion.

These results are summarized in Table S4 in the SI (page S24), and we added the following explanation in the main text.

“The non-CT character was also supported by the TD-DFT calculations assuming solvent polarity with the IEFPCM model (Table S4).”

Reply to Reviewer #2

Ms.ID: oc-2024-002847

Title: **"Optically distinguishable electronic spin isomers of an organic diradical"**

Authors: Daiki Shimizu, Hiakaru Sotome, Hiroshi Miyasaka, and Kenji Matsuda

Thank you for reviewing our manuscript carefully. According to the reviewer's comments, we have revised our manuscript as follows:

Comment (1). Could the authors provide an explanation on the difference between the electronic spin-isomers mentioned this article and the magnetic bistability which was observed in many diradicals.

Answer (1). Thank you for pointing out the important issue. The magnetic bistability of organic radicals has been explained by the structural phase transition in condensed systems. On the other hand, our ideas of spin-isomer and spin bistability are based on a single molecule. From the viewpoint, theoretically, spin states of every diradical can be regarded as spin isomers (if they are distinguishable). To clarify this point, we added the following explanation in the main text (ref. 5).

"Magnetic bistability has been observed for radical crystals, which is explained by the structural phase transition in condensed phases. On the other hand, here we use "spin bistability" as the feature that a single molecule takes two stable spin states."

Comment (2). The free electrons of diradical 1 were located in different N atoms in Figure 2 and Figure 4. Is there a movement of diradical? Or maybe it is a mistake?

Answer (2). Thank you for the comment. It was our mistake, and the structures in Figures 2 and 4 should be the same. We corrected Figure 4.

Comment (3). The last sentence of the last paragraph on page 3, and the line 13 on page 6, please check compound 7 and compound 6?

Answer (3). Thank you for pointing out the wrong compound numbers. These should be compound **5**, and we corrected them accordingly.

Comment (4). In the last sentence on page 5, ground-state bleaching band around 650 nm in Figure 5h, could authors provide an explanation why these is a ground-state bleaching band for diradical 1. However, there is no bleaching band for monomer 5. How to assign 650 nm?

Answer (4). The VT-absorption measurement in Figure 3 and the calculation results in Figure 4 confirm that compound **1** in the singlet state shows a ground-state absorption band around 670 nm. Therefore, a negative band in Figure 5h, which appears as its inverted band, can be attributed to the ground-state bleaching of compound **1**. On the other hand, in the case of monomer **5**, the oscillator strength of the corresponding transition is small, and the excited-state absorption bands appear over the entire wavelength range, so the ground-state bleaching is not seen as a clear negative signal.

To explain the difference, we added the following sentence in the main text.

“In the case of **5** and the triplet state of **1**, the ground-state bleaching is not observed as a clear negative signal due to the small transition intensity of the ground state and the excited-state absorption bands overlapping the entire wavelength range.”
